# New anti-ovarian cancer quinolone derivatives acting by modulating microRNA processing machinery†

**DOI:** 10.1039/d4md00649f

**Published:** 2024-09-27

**Authors:** Tommaso Felicetti, Nicola Di Iacovo, Maria Agnese Della Fazia, Danilo Piobbico, Stefania Pieroni, Martina Pacetti, Jialing Yu, Yilun Sun, Serena Massari, Maria Letizia Barreca, Stefano Sabatini, Oriana Tabarrini, Violetta Cecchetti, Fei Wang, Yves Pommier, Mariangela Morlando, Giuseppe Servillo, Giuseppe Manfroni

**Affiliations:** a Department of Pharmaceutical Sciences, Section of Chemistry and Pharmaceutical Technology, University of Perugia Via Del Liceo 1-06123 Perugia Italy mariangela.morlando@uniroma1.it giuseppe.manfroni@unipg.it +39 06 4991 2341 +39 075 585 5126; b Department of Medicine and Surgery, University of Perugia Piazza L. Severi 1/8-06132 Perugia Italy giuseppe.servillo@unipg.it +39 075 585 8110; c Center for Natural Products Research, Chengdu Institute of Biology, Chinese Academy of Sciences Chengdu 610041 China; d Center for Cancer Research, Developmental Therapeutics Branch & Laboratory of Molecular Pharmacology, NCI 31 Center Drive Bethesda MD 20892-4255 USA

## Abstract

MicroRNAs (miRNAs) play a crucial role in ovarian cancer (OC) pathogenesis and miRNA processing can be the object of pharmacological intervention. By exploiting our in-house quinolone library, we combined a cell-based screening with medicinal chemistry efforts, ultimately leading to derivative 33 with anti-OC activity against distinct cell lines (GI_50_ values 13.52–31.04 μM) and CC_50 Wi-38_ = 142.9 μM. Compound 33 retained anticancer activity against additional cancer cells and demonstrated a synergistic effect with cisplatin against cisplatin-resistant A2780 cells. Compound 33 bound TRBP by SPR (*K*_D_ = 4.09 μM) and thermal shift assays and its activity was TRBP-dependent, leading to modulation of siRNA and miRNA maturation. Derivative 33 exhibited augmented potency against OC cells and a stronger binding affinity for TRBP compared to enoxacin, the sole quinolone identified as a modulator of miRNA maturation. Consequently, 33 represents a promising template for developing novel anti-OC agents with a distinctive mechanism of action.

## Abstract 2

From a quinolone library and subsequent medicinal chemistry optimization, we identified the fluoroquinolone 33, which demonstrated anti-ovarian cancer activity through a mechanism involving TRBP and resulting in the modulation of miRNA maturation.

## Graphical abstract

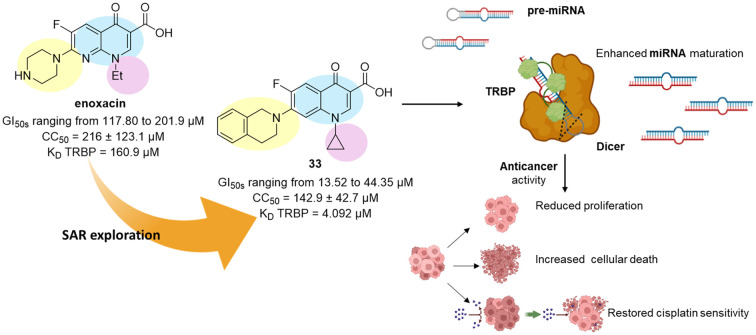

## Introduction

According to GLOBOCAN estimates, in 2020, ovarian cancer (OC) was diagnosed in 313 959 women worldwide, resulting in 207 252 deaths.^1^ OC is the leading cause of death among women diagnosed with gynecologic cancers, making it the fifth most common cause of death in women.^1–3^ Pathologically, most OCs (approximately 90%) originate from the epithelial surface, while a smaller part arises from germ or stromal cells.^4^ Malignant epithelial OCs can be classified into high-grade serous ovarian carcinoma (HGSC), endometrioid carcinoma, clear cell carcinoma, mucinous carcinoma, and low-grade serous ovarian carcinoma.^5^ HGSC is considered to be the most lethal gynecological cancer^6^ and is frequently characterized by p53 mutations (96% of cases), which lead to p53 loss of function and induce HGSC development.^7^ Unfortunately, OCs are often associated with a poor prognosis due to diagnosis at advanced stages of disease and the development of chemotherapy resistance. The standard of care is cytoreductive surgery combined with adjuvant chemotherapy, but 70% of patients experience recurrence within 2 years of primary diagnosis, and the mortality rate reaches approximately 50% of women at five years.^8,9^ The strategy of adjuvant chemotherapy is mainly defined for patients who respond to platinum-based treatments, while approaches for platinum-resistant patients still seem to be poorly defined.^10^ Indeed, for platinum-refractory disease, doxorubicin, paclitaxel, gemcitabine and topotecan are used based on toxicity, treatment cost and accessibility; however, response rates range from 10% to 15% with an overall survival of about 1 year.^11^ In addition, patients with platinum-sensitive OCs become platinum-resistant at advanced stages and acquire chemoresistance through various mechanisms.^12^ Despite new treatments (*i.e.*, olaparib monotherapy or in combination with bevacizumab) recently approved as maintenance therapy for advanced OCs,^13^ we are still far from satisfactory results and novel approaches are warranted.

In recent years, some studies have shown that microRNAs (miRNAs), small non-coding RNAs of approximately 19–22 nucleotides that are involved in fine-tuning gene expression at the post-transcriptional level, play diverse roles in OC by acting as tumor suppressors or proto-oncogenes and also by controlling drug resistance mechanisms.^14^ Attempts to target miRNAs in cancer have been considered, with some small molecules acting as inhibitors of miRNA processing and function appearing to be more successful.^15,16^ Nevertheless, analysis of Dicer expression in invasive epithelial OC specimens from 111 patients has shown that low Dicer expression is significantly associated with an advanced tumor stage.^17^ Since Dicer is a key enzyme involved in the maturation of miRNAs,^18^ its reduced expression results in low levels of miRNAs, suggesting that a strategy aimed at their enhancement may have anticancer effects. In 2008, enoxacin, a fluoroquinolone antibacterial drug, was reported as a small molecule enhancer of miRNA (SMER) processing and exhibited anticancer activity characterized by a broad spectrum of action and an innovative mechanism.^19–21^ However, the SMER property appeared to be structure-dependent and not attributable to the whole class of quinolone molecules; in fact, within a set of approved quinolones and some synthesized enoxacin derivatives, none of the compounds showed activity except ciprofloxacin, which demonstrated to be only a very weak SMER.^19^ Subsequently, enoxacin was reported to be able to inhibit several cancer cell lines with a dose that inhibits the growth of cells by 50% (GI_50_) of 125 μM.^20^ Several groups have confirmed that the anticancer activity of enoxacin was dependent on the modulation of miRNA processing by restoring miRNA expression to physiological levels.^21–25^ Mechanistic studies have shown that enoxacin is able to enhance the transactivation response element RNA-binding protein-(TRBP)-mediated loading of pre-miRNAs on Dicer, by increasing the binding between pre-miRNAs and TRBP, thereby promoting Dicer cleavage at pre-miRNAs.^19–21^ The inactivity of enoxacin as an anticancer agent in TRBP knock-out (KO) cells further confirmed that its anticancer effect is dependent on TRBP.^19–21^

Until 2021, enoxacin remained the only molecule acting as a SMER compound. Then, Fei Wang and colleagues reported the natural product gomisin M1 as an anti-hepatocellular carcinoma agent due to its ability to modulate miRNA processing, thus extending this peculiar mode of action beyond enoxacin and renewing interest in SMER molecules.^26^

**Fig. 1:**
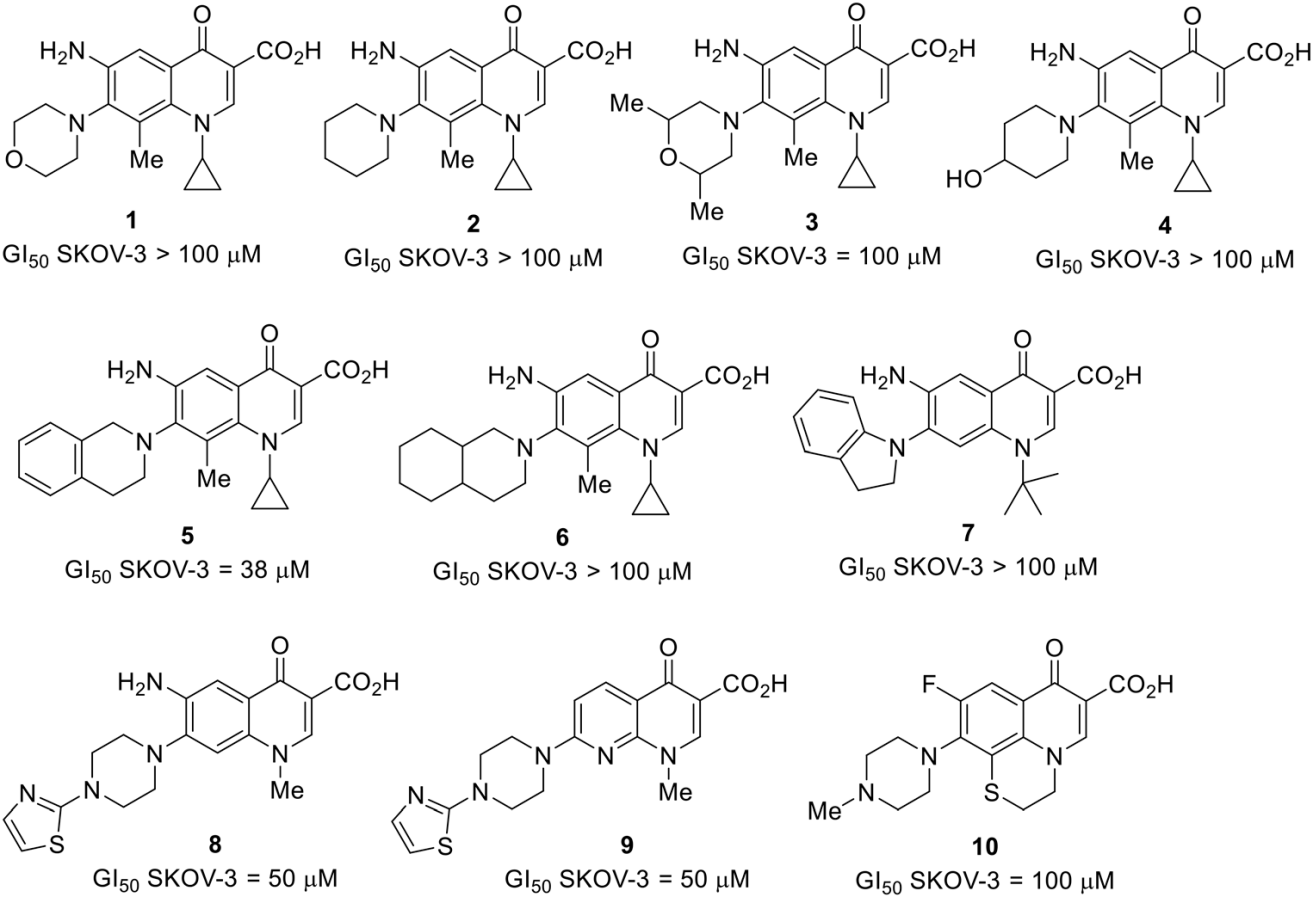
Small set of selected in-house quinolones (1–10) and their antiproliferative activity against the SKOV-3 cell line expressed as GI_50_ values.

To the best of our knowledge, the anticancer activity of enoxacin has never been evaluated against OC cells. Therefore, we were interested to evaluate whether enoxacin (Table 1 for the chemical structure) could retain its anticancer activity against the OC cell line SKOV-3, which does not express p53 at protein and mRNA levels^27^ and is resistant to tumor necrosis factor and other cytotoxic drugs (*i.e.*, diphtheria toxin, cisplatin, and adriamycin).^28–30^ As expected, enoxacin exhibited a GI_50_ value of 125 μM against the SKOV-3 cell line, thus extending its anticancer activity to OC. At this point, given the considerable number of quinolones present in our in-house library, previously synthesized as antibacterial, anti-mycobacterial, antiviral, and anticancer agents,^31–40^ we selected ten of them (compounds 1–10 in Fig. 1) for testing against proliferating SKOV-3 cells. The number of quinolones was restricted due to the inability to perform a high-throughput screening (HTS) campaign and the initial prudence in testing a multitude of quinolones, given the findings of Shan and colleagues that unambiguously demonstrated that the RNAi-enhancing activity of enoxacin was structure-dependent and not universal across all classes of quinolones.^19^ Therefore, the selection was primarily focused on 6-aminoquinolones (8 of the 10 selected), as they have been less extensively explored than 6-fluoro-quinolones. Consequently, it was deemed less probable that they were present in the library of quinolones tested by Shan *et al.*^19^ Notably, compound 10 (rufloxacin), the sole 6-fluoroquinolone in our preliminary screening, was selected due to its status as an approved drug and extensive availability in our in-house library, having been identified by some of us in previous years.^38^

**Table 1:** Antiproliferative effect of compounds against the SKOV-3 cell line and Wi-38 cells. Evaluation of GI_50_ and CC_50_ values

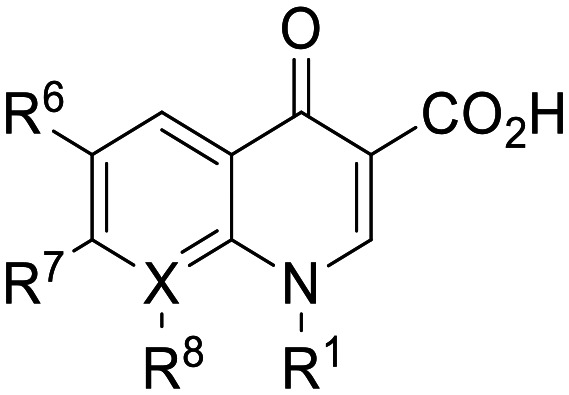							
Compd.	X	R^1^	R^6^	R^7^	R^8^	GI_50_ SKOV-3^a^ (μM)	CC_50_ Wi-38^b^ (μM)
**Enoxacin**	N	–Et	–F	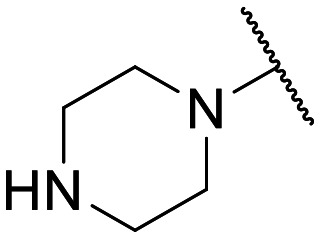	—	125	216 ± 123.1
5	C	–*c*Pr	–NH_2_	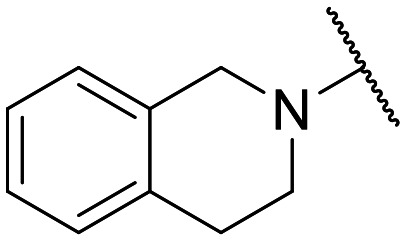	–Me	37.71 ± 3.38	38.51 ± 28.97
11	C	–Et	–NH_2_	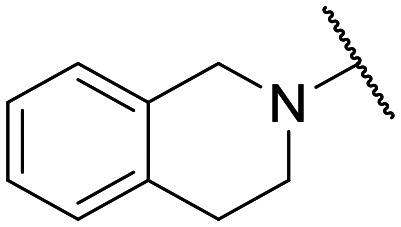	–Me	13.14 ± 1.29	53.50 ± 20.64
12	C	–*c*Pr	–NH_2_	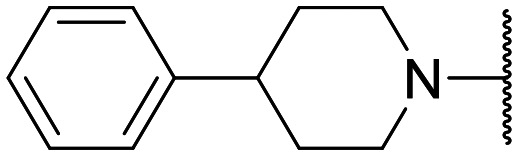	–Me	17.14 ± 2.61	52.96 ± 14.91
13	C	–*c*Pr	–NH_2_	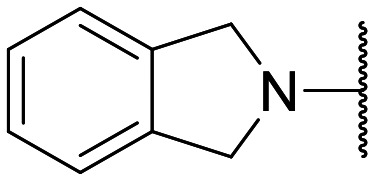	–Me	28.77 ± 4.11	NT^c^
14	C	–*c*Pr	–NH_2_	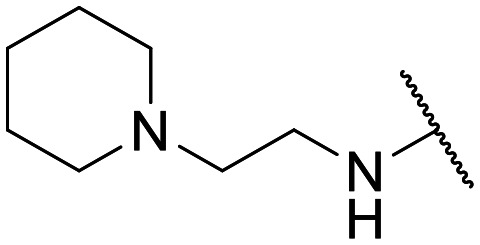	–Me	102.9 ± 21.58	NT
15	C	–*c*Pr	–NH_2_	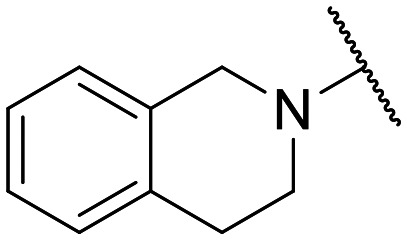	–H	13.2 ± 1.13	36.27 ± 8.86
16	C	–Et	–NH_2_	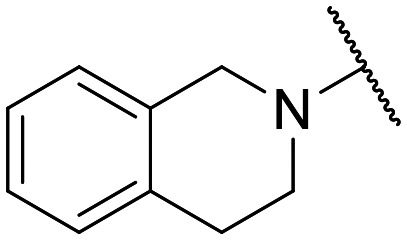	–H	5.44 ± 0.13	8.19 ± 14.3
17	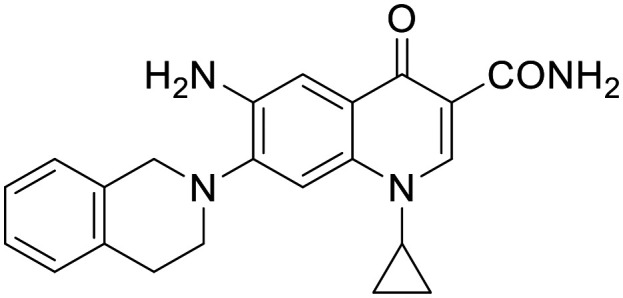					ND^d^	NT
18	C	–*c*Pr	–NH_2_	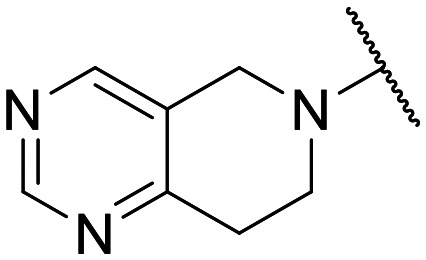	–H	152.3 ± 3.05	NT
19	C	–*c*Pr	–NH_2_	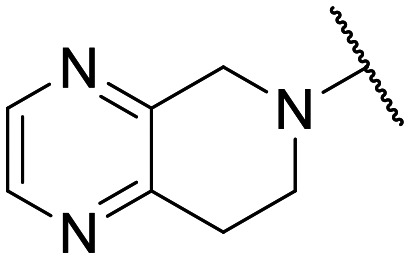	–H	104.3 ± 2.03	NT
20	C	–*c*Pr	–NH_2_	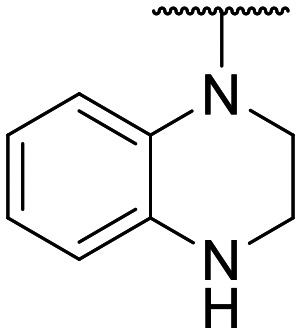	–H	65.42 ± 24.31	NT
21	C	–*c*Pr	–NH_2_	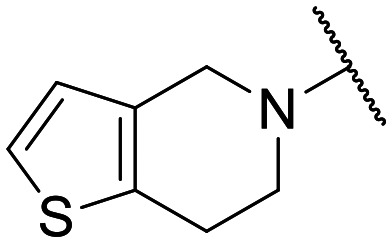	–H	23.66 ± 1.02	103.4 ± 15.45
22	C	–*c*Pr	–NH_2_	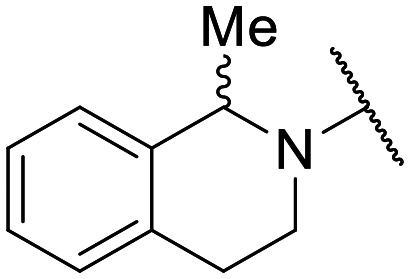	–H	18.52 ± 1.71	59.87 ± 5.95
23	C	–*c*Pr	–NH_2_	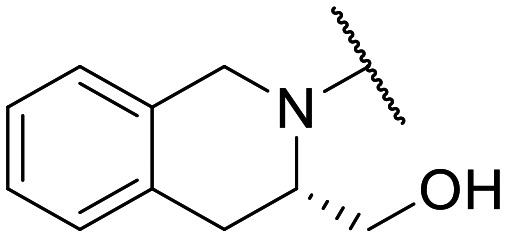	–H	46.17 ± 8.38	NT
24	C	–*c*Pr	–NH_2_	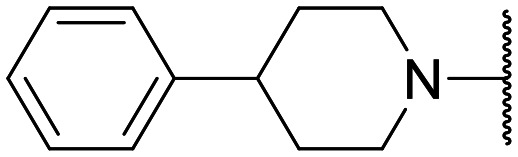	–H	9.19 ± 1.05	45 ± 13.37
25	C	–*c*Pr	–NH_2_	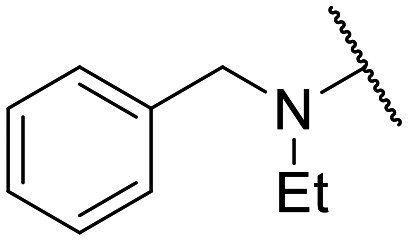	–H	15.99 ± 2.94	79.74 ± 11.79
26	C	–*c*Pr	–NH_2_	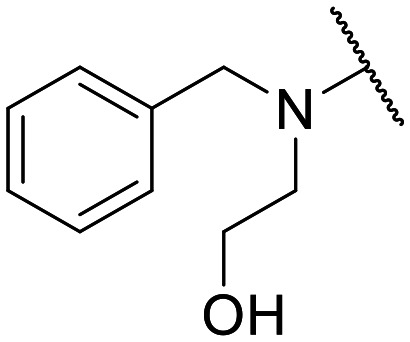	–H	33.93 ± 6.43	NT
27	N	–Et	–F	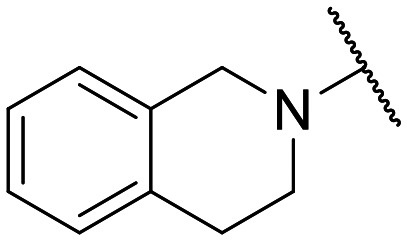	—	ND	NT
28	N	–*c*Pr	–F	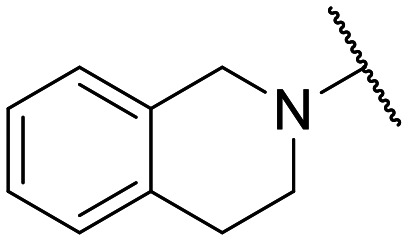	—	46.82 ± 7.44	NT
29	N	–Et	–F	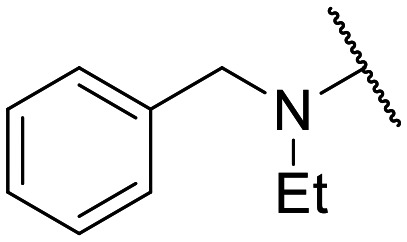	—	16.18 ± 1.03	25.50 ± 11.5
30	N	–Et	–F	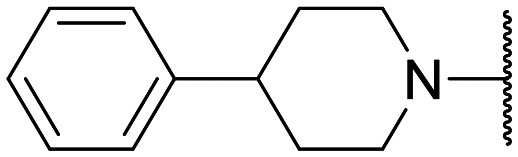	—	8.34 ± 1.28	4.65 ± 5.96
31	C	–Et	–F	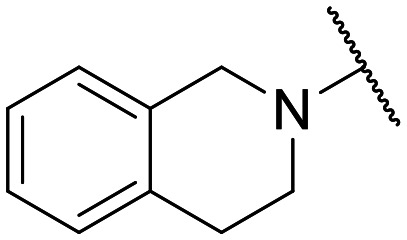	–Me	14.89 ± 3.24	56.58 ± 37.36
32	C	–*c*Pr	–F	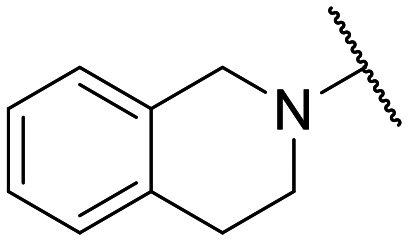	–Me	25 ± 6.04	90.73 ± 30.86
33	C	–*c*Pr	–F	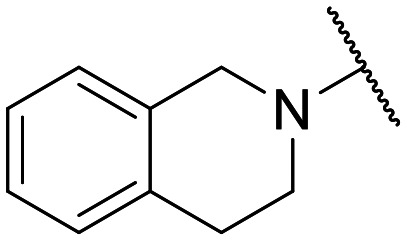	–H	13.52 ± 1.45	142.9 ± 42.7

a GI_50_: 50% cell growth inhibition.

b CC_50_: 50% cytotoxicity concentration.

c NT: not tested; CC_50_ was not determined for compounds showing GI_50_ values ≥25 μM.

d ND: activity not dose dependent.

The encouraging outcomes of this preliminary assessment prompted us to embark on a preliminary medicinal chemistry campaign around the quinolone core, with the objective of developing compounds that exhibit anticancer activity against OC cell lines and act through the modulation of miRNA maturation. The findings of this study led to the identification of novel quinolones with promising anticancer activity, which act as SMER agents by binding to TRBP.

## Results and discussion

### Preliminary screening and design of new quinolones

Preliminary screening of in-house quinolones (compounds 1–10 – Fig. 1) against the SKOV-3 cell line revealed the amino-quinolone 5, characterized by cyclopropyl, tetrahydroisoquinoline and methyl groups at C-1, C-7 and C-8 positions, respectively, as a promising anticancer compound with a GI_50_ of 38 μM (about 3-fold lower than enoxacin). Amino-quinolone derivatives 1–4, 6 and 7, which differ from 5 mainly by the different substituent at the C-7 position, were significantly less active, suggesting that non-aromatic or more polar moieties than tetrahydroisoquinoline at this position are not well-tolerated. The amino-quinolone 8 and the naphthyridone derivative 9, both sharing a thiazolyl-piperazine moiety at the C-7 position, also showed interesting GI_50_ values (50 μM). Finally, the approved drug 10 exhibited poor anticancer activity (GI_50_ = 100 μM), similar to the results obtained by Shan *et al.* who observed that ofloxacin (close analogue of 10) did not show any significant RNAi enhancer effect.^19^

Since many quinolones have been reported in the literature as anticancer agents especially targeting topoisomerases, to exclude this already known mechanism, we evaluated the ability of the amino-quinolone 5 to target human topoisomerases (hTopI and hTopII). The results of cleavage assays conducted at 100 μM (Fig. S1A†) indicated that compound 5 did not trap human topoisomerases. This observation led us to conclude that the anti-OC activity of compound 5 was likely mediated by a topoisomerase-independent mechanism of action.

Encouraged by this evidence, we considered the possibility of testing additional quinolones from our in-house library. However, we reasoned that the data from the preliminary screening strongly indicated that the tetrahydroisoquinoline, as a C-7 substituent of the 6-aminoquinolone scaffold, played a pivotal role in conferring the anticancer activity observed in compound 5. Consequently, we concluded that it was not advantageous to pursue the selection of additional 6-aminoquinolones from our in-house library, as they lacked sufficient structural similarity to facilitate the investigation of a structure–activity relationship (SAR) surrounding compound 5. Conversely, we decided to use derivative 5 as a starting hit compound and started the design of a series of chemical modifications (Table 1 for chemical structures) by replacing the cyclopropyl at C-1 with an ethyl group (derivative 11) or the tetrahydroisoquinoline at the C-7 position with three different moieties (derivatives 12–14). Subsequently, removal of the C-8 methyl group of 5 and 11 led to the synthetically more accessible C-8 des-methyl analogues 15 and 16, respectively. After evaluating the role of the C-3 carboxyl function by designing the amide derivative 17, further efforts were focused on the C-7 substituent of 15, which was replaced with different groups, yielding derivatives 18–26. The resulting best C-7 substituents were then used to design close analogues of enoxacin (derivatives 27–30). Finally, since tetrahydroisoquinoline was found as the best moiety for the C-7 position, we planned the synthesis of fluoro-quinolones 31–33. Of note, compounds 32 and 33 were previously published by some of us as comparative analogues of antibacterial amino-quinolones.^34^ However, both compounds were no longer available in our in-house library, thus we synthesized them using optimized procedures different from those previously reported.^34^

### Chemistry

**Scheme 1:**
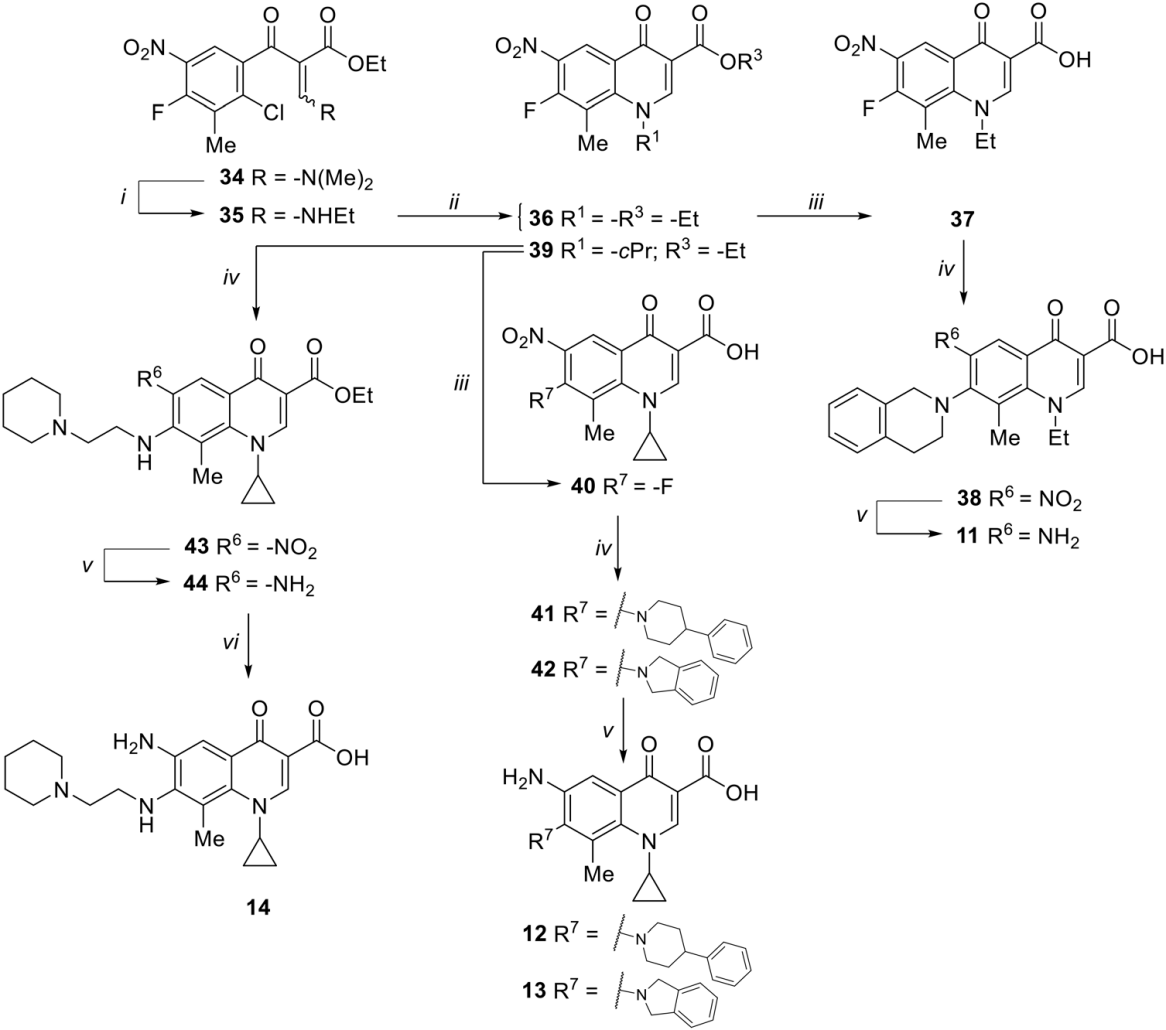
Reagents and conditions: i) ethylamine, Et_2_O/EtOH, rt, 15 min; ii) K_2_CO_3_, DMF, 100 °C, 1 h; iii) 6 N HCl, EtOH, reflux, 1 h; iv) amine, Et_3_N, dry DMF or DMSO, 80–100 °C, 1–2 h; v) RANEY®/Ni, H_2_, DMF, rt, 30 min–12 h; vi) 10% NaOH, MeOH, reflux, 1 h.

Target compounds 11–14 were synthesized as reported in Scheme 1. Reaction of the acrylate intermediate 34 (ref. 33) with ethylamine in a mixture of Et_2_O and EtOH afforded derivative 35, which was cyclized in dry DMF in the presence of K_2_CO_3_ to give the quinolone intermediate 36. Subsequent hydrolysis of 36 with a mixture of 6 N HCl and EtOH afforded the quinolone acid derivative 37, which was reacted with tetrahydroisoquinoline in dry DMF in the presence of Et_3_N as a base to give compound 38. Under H_2_ flow, RANEY®/Ni catalyzed reduction in DMF afforded the target compound 11. Similarly, acid hydrolysis of quinolone ester 39 (ref. 33) afforded the corresponding acid 40,^33^ which was reacted with 4-phenylpiperidine or isoindoline in dry DMSO in the presence of Et_3_N as a base to afford C-7 substituted quinolone derivatives 41 and 42. Subsequently, nitro group reduction afforded the target compounds 12 and 13. On the other hand, starting from the ester intermediate 39 (ref. 33) and exploiting the good nucleophilic property of 2-amino-ethylpiperidine, the nitro derivative 43 was first obtained and then reduced to 44 and hydrolyzed under basic conditions to give the target compound 14.

**Scheme 2:**
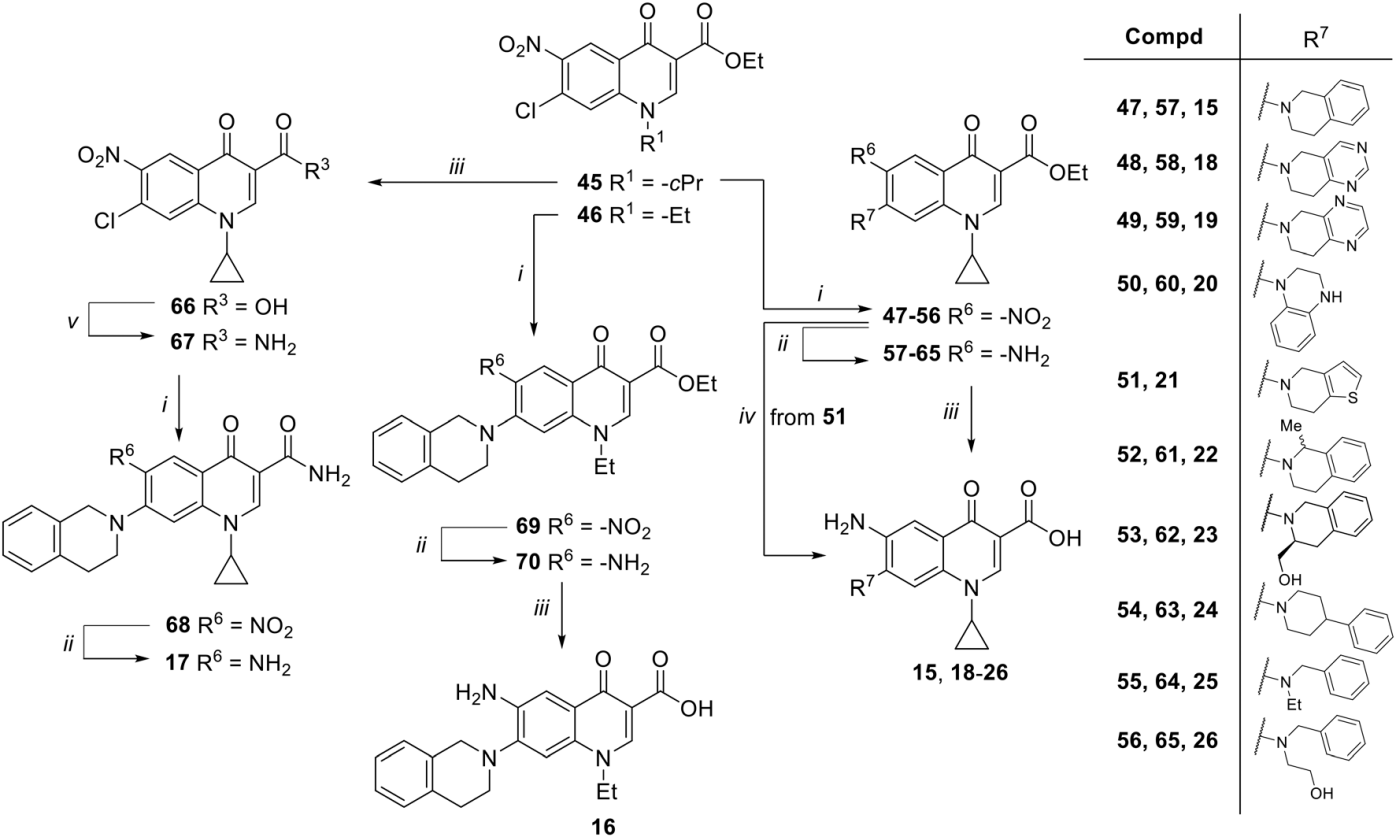
Reagents and conditions: i) amine, Et_3_N, dry DMF or DMSO, 80–110 °C, 90 min–48 h; ii) RANEY®/Ni, H_2_, DMF, rt, 1–6 h; iii) 6 N HCl, EtOH, reflux, 9 h (for 15) or 10% NaOH, MeOH, reflux, 30 min–4 h; iv) SnCl_2_, EtOH, reflux, 2 h; v) SOCl_2_, reflux, 2 h; *then*, 7 N NH_3_ in MeOH, dry DMF, rt, 3 h.

Target compounds 15–26 were synthesized as reported in Scheme 2. Reaction of nitro-quinolone derivative 45 (ref. 31) with various amines in dry DMF or DMSO in the presence of Et_3_N as a base afforded C-7 substituted quinolone analogues 47–56. Subsequent hydrogenation under a catalytic amount of RANEY®/Ni of 47–50 and 52–56 afforded amino derivatives 57–65, which were then hydrolyzed to target compounds 15, 18–20 and 22–26. On the other hand, derivative 21 was obtained directly from the corresponding nitro ester derivative 51 by reaction with SnCl_2_ in EtOH at reflux. In parallel, the ester derivative 45 was also converted to the corresponding acid to give intermediate 66, which was reacted with SOCl_2_ and then treated with 7 N NH_3_ in MeOH and dry DMF to give the amide intermediate 67. Nucleophilic substitution of 67 with 1,2,3,4-tetrahydroisoquinoline in dry DMSO in the presence of Et_3_N gave the C-7 substituted intermediate 68, which was subjected to RANEY®/Ni catalyzed reduction of the nitro group to give the target compound 17. Starting from derivative 46,^37^ nucleophilic substitution with 1,2,3,4-tetrahydroisoquinoline in dry DMSO using Et_3_N as a base afforded derivative 69, which was first reduced to amino derivative 70 and then hydrolyzed under basic conditions to give target compound 16.

**Scheme 3:**
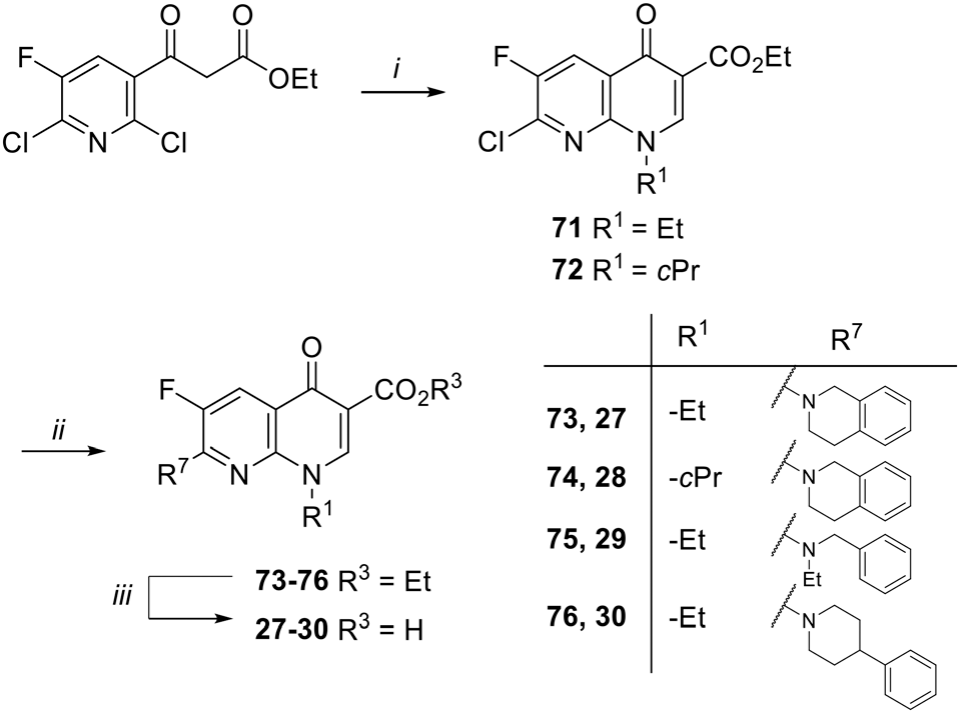
Reagents and conditions: i) DMF–DMA, AcOH, dry toluene, rt, 2 h; *then*, ethylamine or cyclopropylamine, rt, 30 min; *then*, 1.5 M Bu_4_NOH, rt, 5 min; ii) amine, Et_3_N, dry DMF, 90 °C, 2–3 h; iii) 6 N HCl, EtOH, reflux, 4–16 h or 10% NaOH, MeOH, reflux, 1 h (for 29).

Target compounds 27–30 were synthesized as reported in Scheme 3. Starting from commercially available ethyl 3-(2,6-dichloro-5-fluoropyridin-3-yl)-3-oxopropanoate, naphthyridone derivatives 71 and 72 were synthesized by a one-pot procedure involving the initial reaction with DMF–DMA, a catalytic amount of AcOH and dry toluene, followed by the addition of alkylamines (ethylamine or cyclopropyl amine) and then tetrabutylammonium hydroxide. The reaction of 71 and 72 with different amines in dry DMF and in the presence of Et_3_N afforded the ester derivatives 73–76, which were hydrolyzed to give the target compounds 27–30.

**Scheme 4:**
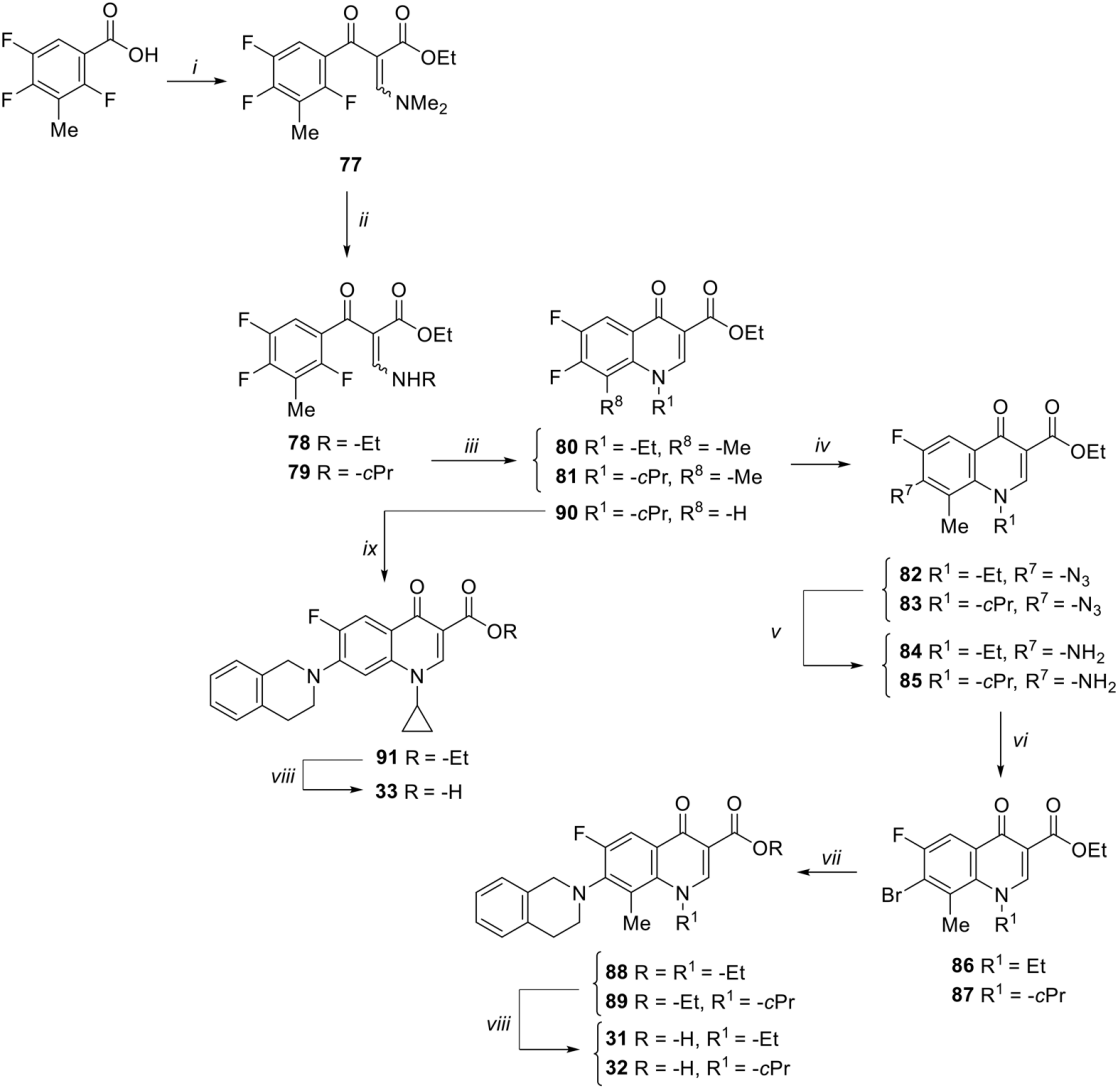
Reagents and conditions: i) C_2_O_2_Cl_2_, cat. dry DMF, dry CH_2_Cl_2_, rt, 4 h; *then*, ethyl 3-(*N*,*N*-dimethylamino)acrylate, Et_3_N, dry toluene, 90 °C, 4 h; ii) ethylamine or cyclopropylamine, Et_2_O, EtOH, rt, 1 h; iii) NaH, dry DMF, 0 °C, 3 h; iv) NaN_3_, dry DMF, 90 °C, 24 h; v) Pd/C, H_2_, DMF, rt, 3 h; vi) CuBr_2_, 5% HBr, 2 M NaNO_2_, 0 °C to rt, 30 min; vii) 1,2,3,4-tetrahydroisoquinoline, Pd_2_(dba)_3_, BINAP, Cs_2_CO_3_, dry toluene, reflux, 13 h; viii) 6 N HCl, EtOH, reflux, 1–4 h; ix) 1,2,3,4-tetrahydroisoquinoline, Et_3_N, dry DMSO, 100 °C, 4 h.

The synthesis of target compounds 31–33 is reported in Scheme 4. Although the synthetic procedure of analogue 32 has been reported previously,^34^ we encountered some problems related to the formation and handling of the difluoroborate complex envisaged in the published procedure;^34^ therefore, a different chemical synthesis was carried out to obtain both C-8 methyl derivatives 31 and 32. The new procedure involved the chlorination of commercial 2,4,5-trifluoro-3-methylbenzoic acid by oxalyl chloride, catalyzed by dry DMF, in dry CH_2_Cl_2_ to give the corresponding acyl chloride, which was immediately reacted with ethyl 3-(*N*,*N*-dimethylamino)acrylate in dry toluene to give intermediate 77. Reaction of 77 with ethylamine or cyclopropylamine in a mixture of Et_2_O and EtOH afforded derivatives 78 and 79, respectively. Cyclization of 78 and 79 was performed with NaH in dry DMF at 0 °C to give 6-fluoro quinolone intermediates 80 and 81,^41^ respectively. Since various attempts to introduce the tetrahydroisoquinoline moiety at the C-7 position of the 6-fluoro-8-methyl quinolone by nucleophilic substitution were unsatisfactory, we explored a different procedure by modifying a previously reported chemical synthesis.^42^ Accordingly, derivatives 80 and 81 were reacted with NaN_3_ in dry DMF to give derivatives 82 and 83, which were then reduced by H_2_ flow with a catalytic amount of Pd/C to give amino derivatives 84 and 85. Subsequently, a Sandmeyer reaction allowed the synthesis of bromo analogues 86 and 87, which were reacted with tetrahydroisoquinoline in a Buchwald–Hartwig reaction to give C-7 functionalized quinolone ester derivatives 88 and 89. Finally, acid hydrolysis afforded the target compounds 31 and 32.

On the other hand, the synthesis of C-8 des-methyl quinolone 33 was performed by a standard procedure starting with the nucleophilic substitution of derivative 90,^43^ with the 1,2,3,4-tetrahydroisoquinoline to give intermediate 91. Subsequent acid hydrolysis afforded the target compound 33.

### Biological results

All the compounds were first tested for their ability to inhibit SKOV-3 proliferation at scalar concentrations, which allowed the determination of GI_50_ values (Table 1). In parallel, for compounds with GI_50_ ≤ 25 μM, CC_50_ values were determined in Wi-38 cells to observe any toxic effect against non-tumor cells (Table 1) and to evaluate any potential specific effect against tumor cells.

Replacement of the cyclopropyl ring of 5 with an ethyl group resulted in 11 that showed an increase in antiproliferative activity (GI_50_ 13.14 μM *vs.* SKOV-3) coupled with a CC_50_ value of 53.50 μM on Wi-38 cells, being approximately 4-fold more toxic against tumor cells. Notably, cytotoxicity experiments of the first hit compound 5, performed for comparison, showed a poor selectivity towards OC cell lines with respect to Wi-38 cells, with a CC_50_ value comparable to its GI_50_.

Similar to 11, good results were obtained when the tetrahydroisoquinoline of 5 was replaced by a phenylpiperidine moiety (compound 12) giving a GI_50_ of 17.14 μM against SKOV-3 and a CC_50_ in Wi-38 of 52.96 μM. On the other hand, when isoindoline and the more polar aminoethyl-piperidine moiety were used to replace the tetrahydroisoquinoline of 5, we obtained compounds 13 and 14, which showed a comparable or worse profile than compound 5. Removal of the C-8 methyl group of 5 led to the more potent compound 15 (37.71 μM *vs.* 13.2 μM), which achieved a modest selective anticancer effect. On the other hand, derivative 16, the C-8 des-methyl analogue of 11, showed an improvement in anticancer potency but also an increase in non-tumor cell toxicity compared to 11. Due to synthetic accessibility, the removal of the C-8 methyl group from the amino-quinolone scaffold led to an improvement in terms of yield and reaction time. Therefore, we decided to focus our efforts on 15 and evaluate the role of the carboxyl function by replacing it with an amide group (derivative 17), which was completely inactive. We then replaced the tetrahydroisoquinoline of 15 with more polar bicyclic heterocycles, thus obtaining compounds 18–20, which were less active, suggesting that the benzene ring of the tetrahydroisoquinoline seemed to be important to retain a potent anticancer effect, while the presence of a free NH moiety was detrimental. On the other hand, the bioisosteric replacement of the benzene ring of the tetrahydroisoquinoline of 15 with a thiophene moiety yielded the tetrahydrothienopyridine derivative 21, which retained a GI_50_ comparable to that of 15, while exhibiting a significantly higher CC_50_ value (103.4 μM *vs.* 36.27 μM), thus highlighting a good propensity to act preferentially on tumor cells. Modifications of the aliphatic part of the tetrahydroisoquinoline by introducing a methyl at the C-1′ position (compound 22) or a methyl alcohol at C-3′ (compound 23) did not lead to improvements in terms of anti-proliferative activity. Interestingly, good results were obtained when less rigid moieties were introduced at the quinolone C-7 position to replace the classical tetrahydroisoquinoline. Indeed, 24 and 25 showed comparable GI_50_ values to 15 coupled with favorable CC_50s_. However, when we tried to introduce a free alcoholic group to 25 in order to improve the polarity, we obtained compound 26, which showed a 2-fold increase in GI_50_ (33.93 μM *vs.* 15.99 μM). At this point, we considered introducing the best C-7 substituents identified so far for the quinolone scaffold in place of the piperazine ring of the naphthyridone nucleus of enoxacin. However, compounds 27–30 were less active than their respective quinolone analogues and in some cases very toxic when tested in Wi-38 cells (compounds 29 and 30). Ultimately, given the favorable outcomes observed with tetrahydroisoquinoline in the C-7 position when integrating the SAR data from amino-quinolone and naphthyridone derivatives, our research shifted its focus to fluoroquinolones. To this end, we synthesized three distinct molecules incorporating this moiety (compounds 31–33). Interestingly, compound 31, the close analogue of 11, showed a similar activity to 11 both in terms of anti-proliferative activity and cytotoxicity on non-tumor cells, suggesting a similar behavior for compounds possessing NH_2_ and F groups at the C-6 position. On the other hand, the replacement of the amino group of 5 with a fluorine yielded 32, which exhibited a GI_50_ of 25 μM and a CC_50_ of 90.73 μM, making it significantly less toxic to non-tumor cells than its amino analogue. Finally, the best results were obtained when the amino group of 15 was replaced by a fluorine to yield 33, which showed a comparable GI_50_ to 15 (13.52 μM), but a significant reduction in non-tumor cell toxicity (CC_50_ on Wi-38 = 142.9 μM). Compound 33 thus emerged as a promising fluoroquinolone worthy of further biological profiling.

### Proliferation inhibitory effect of compound 33 against different OC cell lines

Based on the promising results of compound 33 regarding its anti-proliferative activity against SKOV-3 cell lines, coupled with a marked tendency to act preferentially on tumor cells, we decided to investigate whether its activity could be extended to two additional OC cell lines: OVCA433 and A2780 (Table 2).

**Table 2:** Antiproliferative effect reported as GI_50_ against different OC cell lines

Compd.	OC cell lines	
	GI_50_ OVCA433 (μM)	GI_50_ A2780 (μM)
33	31.04 ± 6.75	18.13 ± 8.25
**Enoxacin**	117.80 ± 8.89	142.33 ± 8.73

Notably, 33 exhibited anti-proliferative activity against both OC cell lines, although it showed a slight increase in GI_50_ values. However, in both cases, 33 was significantly more potent than enoxacin, highlighting its potential as a “broad-spectrum” anti-OC agent.

### Investigation on the mode of action of compound 33

As previously evaluated for compound 5, we assessed whether our best compound 33 acted by inhibiting or trapping hTopI and hTopII. As expected, 33 showed no inhibition or trapping activity of human topoisomerases when tested at 100 μM (Fig. S1B†), thus ruling out that the toxicity observed in SKOV-3 was due to topoisomerase inhibition/trapping.

**Fig. 2:**
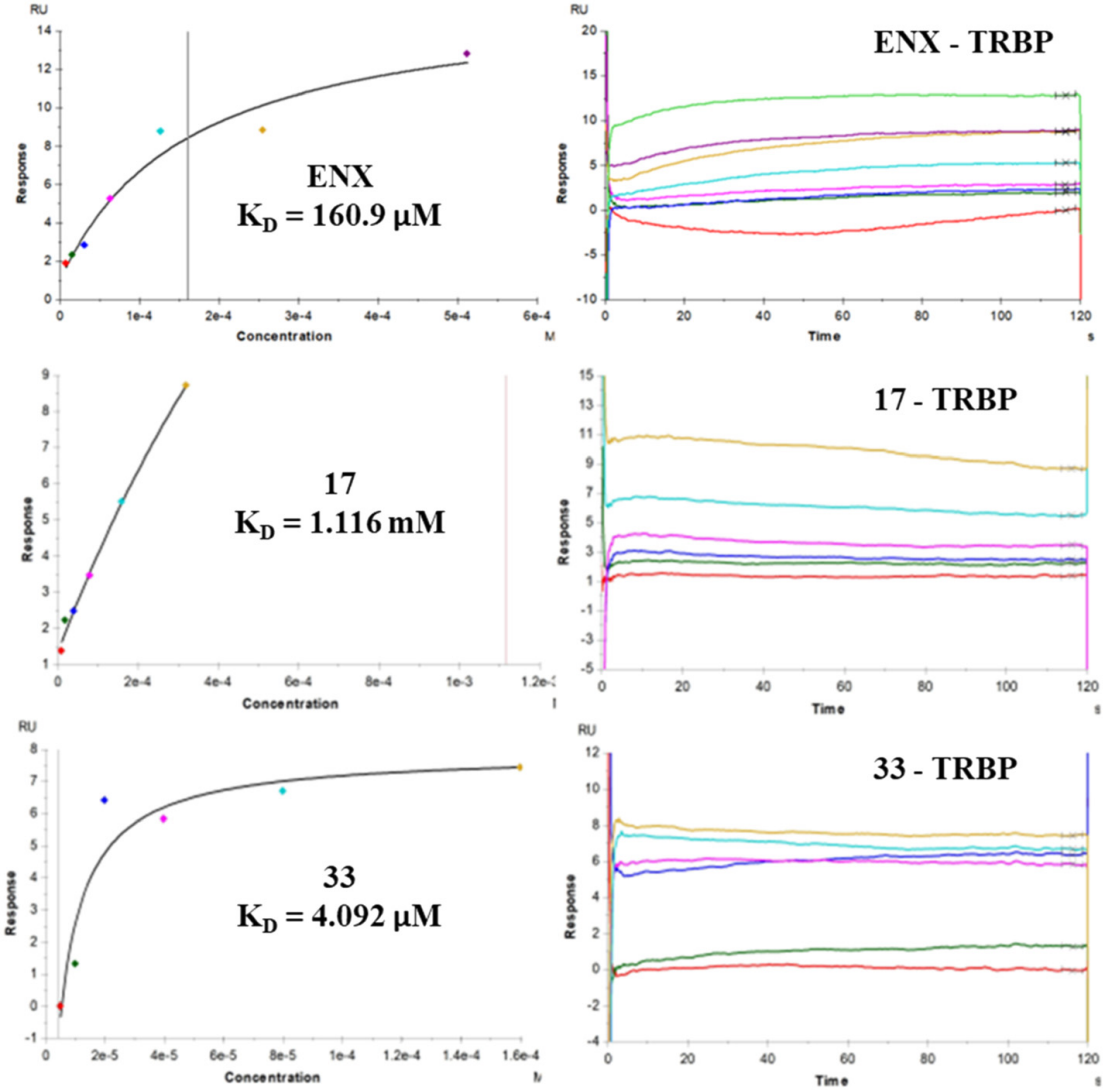
SPR analyses of enoxacin (ENX), 17 and 33 towards TRBP.

Subsequently, to evaluate whether compound 33 acted through an enoxacin-like mechanism, we performed SPR experiments on TRBP for compound 33 and the inactive amide derivative 17 as a negative control (Fig. 2 – enoxacin as a positive control). Interestingly, 33 bound TRBP with a *K*_D_ value of 4.09 μM, whereas derivative 17 showed a very high *K*_D_ value of 1.12 mM. It is also noteworthy that compound 33 exhibited a binding affinity approximately 40-fold stronger for TRBP than enoxacin.

**Fig. 3:**
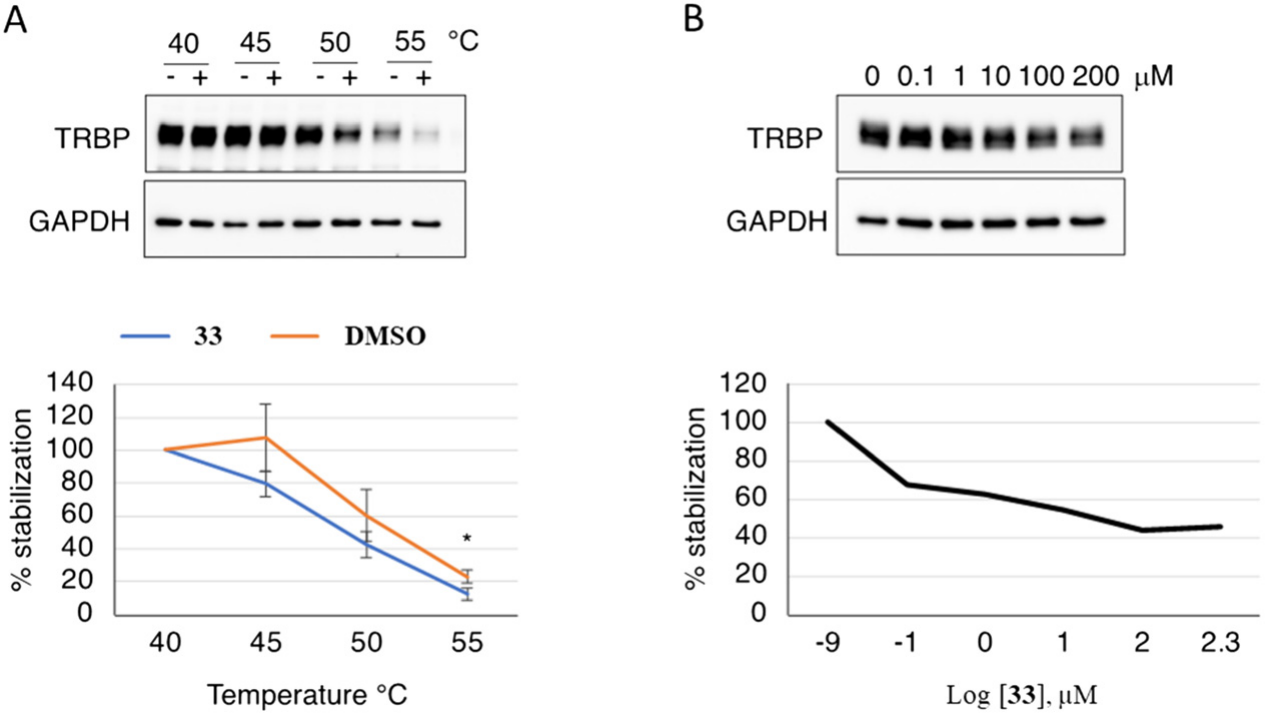
Cellular thermal shift assays of TRBP in the presence of 33. A) Representative Western blot analysis showing the destabilization effect of 33 on TRBP at different temperatures. Graph showing the quantified levels of TRBP over GAPDH from three independent experiments. B) Western blot analysis showing the concentration dependency of the destabilization effect of 33 on TRBP at 55 °C. Graph showing the quantified levels of TRBP over GAPDH. Error bars represent SE. *p*-Value was calculated using paired two-tailed Student's *t*-test (* *p* < 0.05).

A cellular thermal shift (Fig. 3A and B) further confirmed the involvement of TRBP in the mechanism of action of compound 33. In fact, treatment of TRBP with 50 μM of 33 resulted in a significant thermal shift when protein destabilization was compared with DMSO treatment (Fig. 3A). In addition, 33 was able to induce destabilization of TRBP in a concentration-dependent manner (Fig. 3B). Higher melting values are typical in thermal shift assays when a ligand binds to a protein. However, the destabilization of TRBP after treatment with compound 33 is similar to what has been observed with other TRBP binders, such as the SMER gomisin M1 (ref. 26) and the sulfonamide derivative CIB-3b, which was previously reported to inhibit miR-21 maturation.^44^

**Fig. 4:**
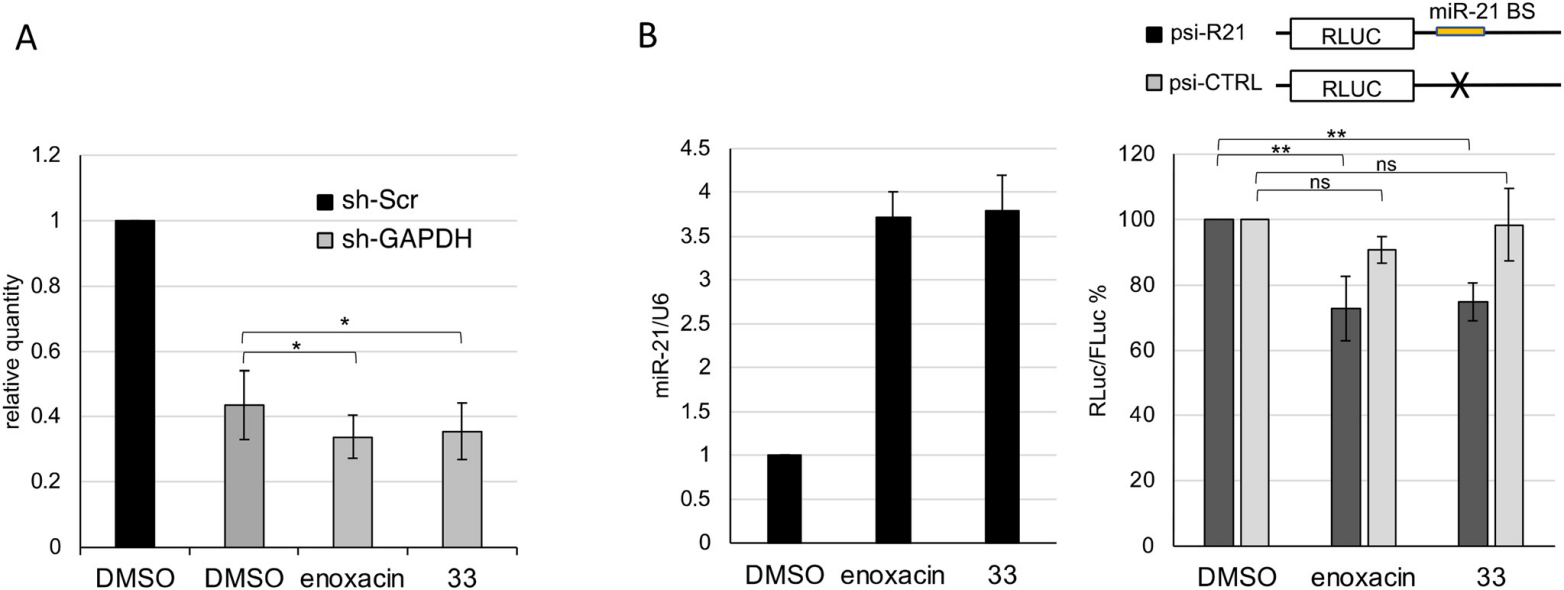
Compound 33 promotes siRNA/miRNA maturation. A) RNAi enhancing effect of compound 33 and enoxacin at 50 μM. Values are shown as relative quantity with respect to sh-Scr/DMSO treated cells set to a value of 1. ATP5O is used as a reference gene. B) Left graph: Upregulation of miR-21 in HeLa cells upon 6 hours of treatment with 33 and enoxacin at 50 μM. Values are shown as relative quantity with respect to DMSO treated cells set to a value of 1. U6 is used as a reference gene. Upper: Schematic representation of the psi-R21 and psi-CTRL constructs. “miR-21 BS” depicts the binding site of the miRNA. Lower graph: miRNA enhancing effect of compound 33 and enoxacin at 50 μM. Values are shown as the ratio between Renilla (RLuc) and firefly (FLuc) luciferase signals. Error bars represent SE of at least three independent experiments. *p*-Values were calculated using paired two-tailed Student's *t*-test (* *p* < 0.05; ** *p* < 0.01; ns: not significant).

To further confirm the role of TRBP in the mechanism of action of 33, we also examined its ability to promote shRNA to siRNA maturation (RNAi enhancing activity) (Fig. 4A). Notably, compound 33 at 50 μM exhibited a modest but significant RNAi enhancing effect by decreasing the amount of GAPDH mRNA, indirectly indicating an increase in siRNA maturation compared to DMSO. This effect was comparable to that of enoxacin tested at 50 μM. We also tested the ability of 33 to promote the maturation of endogenous miRNAs using a luciferase reporter construct carrying the miR-21-5p binding site downstream of Renilla cDNA (psi-R21). As shown in Fig. 4B, 33 produced significant positive effects on miR-21-5p biogenesis and this caused a decrease in Renilla luciferase activity. Notably, this effect parallels that obtained with the positive control enoxacin. The effect is specific since no significant variation was observed when using a control construct, psi-CTRL, which lacks the miR-21-5p binding site.

**Fig. 5:**
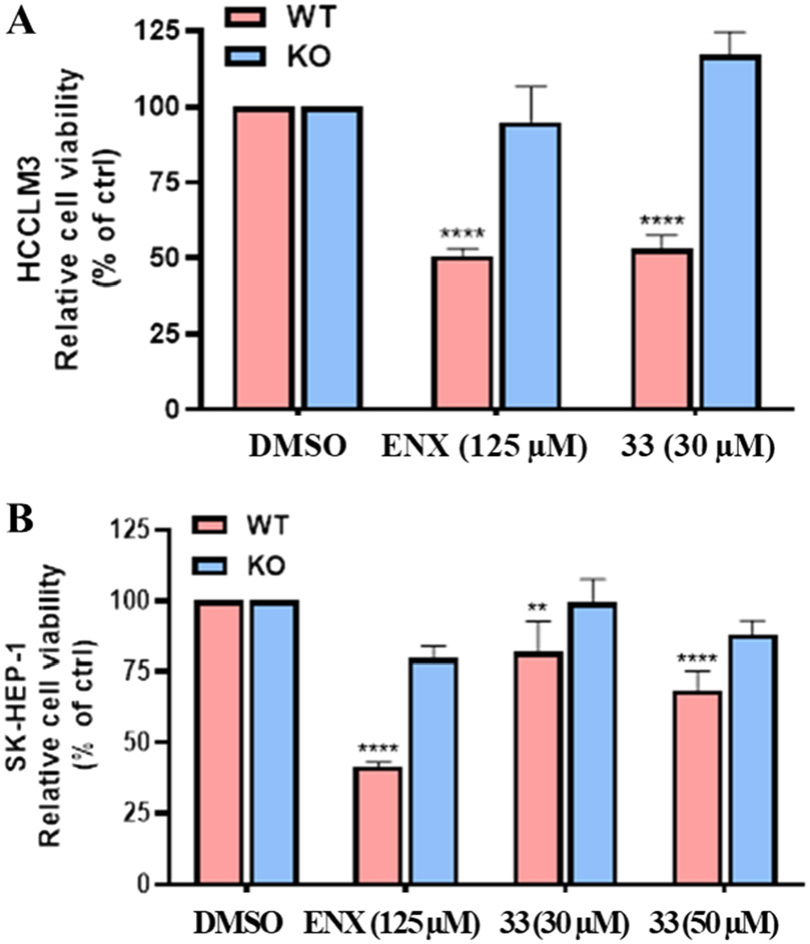
Antiproliferative effect after 48 hours of enoxacin at 125 μM and compound 33 A) against WT and KO TRBP HCCLM3 cell lines (30 μM) and B) against WT and KO TRBP SK-Hep-1 cell lines (30 and 50 μM). *T*-Test results (** *P* ≤ 0.01, **** *P* ≤ 0.001).

To confirm that the anticancer activity of 33 was directly dependent on TRBP, we evaluated its anti-proliferative effect on HCCLM3 and SK-Hep-1 wild-type (WT) and KO cells for TRBP (enoxacin was used as a control – Fig. 5). Notably, after 48 hours, 33 at 30 μM showed no antiproliferative effect on both KO TRBP cells, while it significantly reduced (about 50%) the viability of WT HCCLM3 cells. On the other hand, the anti-proliferative effect of 33 at 30 μM on WT TRBP SK-Hep-1 cells was less evident and thus, it was also tested at 50 μM on both KO and WT cells. Of note, 33 at 50 μM significantly reduced the viability of WT SK-Hep-1 cells while not affecting KO TRBP SK-Hep-1 cells. Taken together, these data confirmed that 33 exerts its anti-proliferative activity by acting on TRBP and thus likely exhibits behavior similar to that of enoxacin. Furthermore, it can be stated that compound 33 retained anticancer activity against cancer cell lines other than those of ovarian origin, including HCCLM3 (hepatocellular carcinoma) and SK-Hep-1 (liver adenocarcinoma).

As previously stated in the introduction, this ability was observed for enoxacin as well, which retained anticancer activity against a wide panel of cancer cells with GI_50_ values of approximately 125 μM.^20^ This valuable broad-spectrum anticancer activity is to be attributed to its distinctive mechanism of action, which enables it to re-regulate dysregulated miRNAs in cancer cells. As one of the objectives of the study was to identify novel quinolones with an enoxacin-like mechanism, further investigation was conducted to ascertain whether compound 33 could exert any antiproliferative effect against three additional cancer cell lines: MCF-7 (breast cancer), A549 (lung carcinoma), and HCT-116 (colon cancer). It is noteworthy that all of these cell lines can be considered SMER-sensitive, as enoxacin has previously demonstrated efficacy against them.^20^ Interestingly, treatment with 33 (30 μM) for 72 hours resulted in a significant inhibitory effect on cell proliferation in all three cell lines (Fig. S2†), indicating that compound 33 possesses broad-spectrum anticancer potential.

### Compound 33 induced A2780 cell death and sensitized cisplatin-resistant A2780 cells to cisplatin

**Fig. 6:**
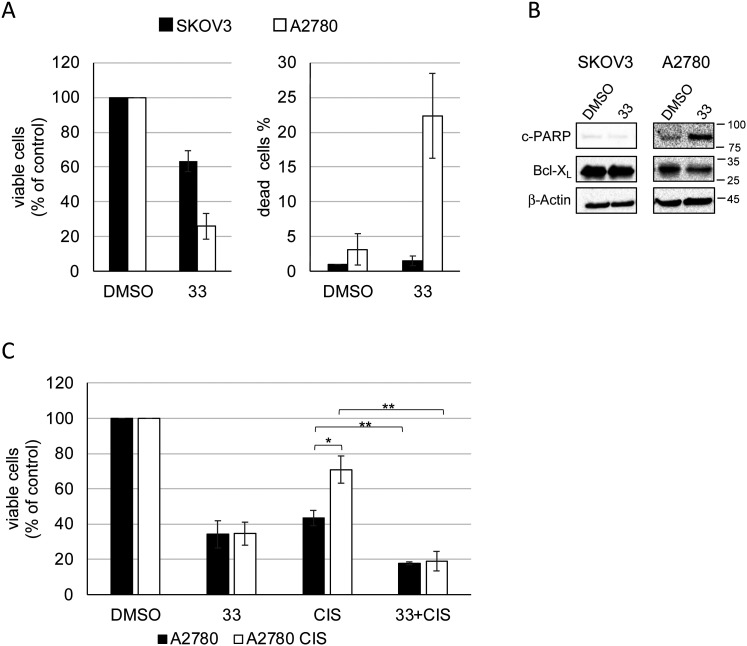
Compound 33 inhibited cell growth and induced apoptosis of A2780 cells and sensitized A2780 CIS to cisplatin. A) Left graph: Reduction of number of SKOV-3 and A2780 viable cells upon treatment with compound 33 at 30 μM for 72 hours. The number of viable cells is expressed as percentage with respect to DMSO condition set to a value of 100%; right graph: percentage of SKOV-3 and A2780 dead cells upon treatment described in “A”; B) representative Western blot analysis of protein extracts from SKOV-3 and A2780 cells treated as in “A”.  β-Actin levels are used as a control for protein loading. C) Graph showing the number of viable cells of a culture of A2780 and A2780 CIS treated with 33 (30 μM), cisplatin (CIS, 1 μM) and a combination of the two compounds (33 + CIS). The number of viable cells is expressed as percentage with respect to DMSO condition set to a value of 100%. Error bars represent SE of at least three independent experiments. *p*-Values were calculated using paired two-tailed Student's *t*-test (* *p* < 0.05; ** *p* < 0.01).

We next tested whether the growth inhibition exerted by 33 on SKOV-3 and A2780 cells was accompanied by induction of cell death. Thus, we treated both cell lines with 33 at 30 μM for 72 hours and cells were counted using trypan blue solution in order to assess cell viability. Fig. 6A shows that after treatment, the total number of cells was greatly reduced for both cell lines, while only for A2780 was the percentage of dead cells increased to a great extent. This result was confirmed by the increased levels of cleaved PARP1 (c-PARP), a marker of apoptosis,^45^ and by the downregulation of the anti-apoptotic protein Bcl-X_L_^46^ in treated A2780 cells (Fig. 6B). No changes were observed in SKOV-3 cells, where compound 33 may exert a cytostatic effect as previously shown for cisplatin treatment.^47^

In consideration of the observed capacity of compound 33 to markedly elicit A2780 cell death, we proceeded to examine the potential synergistic effects of 33 in combination with cisplatin in view of a potential future combined treatment. Cisplatin is an effective first-line treatment for OC, but resistance is common. Although about 80% of OC patients are initially sensitive to cisplatin, many develop resistance, leading to high mortality within a few years. Various cellular processes contribute to platinum drug-resistance in OC including processes regulated by miRNAs. Indeed, it has been reported that modulation of the expression of specific miRNAs can reverse cisplatin resistance in human OC cells.^48^

Therefore, cisplatin-resistant A2780 cells (A2780 CIS)^49^ were treated with 33, cisplatin and with a combination of the two compounds for 48 hours. In parallel, the same experiments were performed using the A2780 cell line. Fig. 6C illustrates that, as expected, A2780 CIS exhibited significantly greater resistance to cisplatin compared to A2780, while both cell lines showed equal sensitivity to treatment with compound 33. Notably, the combination of cisplatin and compound 33 sensitized A2780 CIS to cisplatin and produced a more pronounced inhibitory effect on A2780 proliferation, suggesting a synergistic mechanism of action.

## Conclusions

In this study, we aimed to identify new SMER compounds by exploiting our in-house library of quinolone derivatives. Preliminary evaluation of the antiproliferative activity of in-house derivatives against the OC SKOV-3 cell line led us to identify the 6-amino quinolone derivative 5 as a starting hit worthy of further exploration. Our medicinal chemistry efforts culminated in the identification of 33 as the best compound of the new series, exhibiting a GI_50_ of 13.52 μM against SKOV-3 cells, about 9-fold lower than the known SMER enoxacin and less toxic against non-tumor cells (CC_50_ on Wi-38 = 142.9 μM). Although featured by the presence of the most studied C-6 fluorine atom, like for enoxacin, compound 33 is characterized by an atypical base (tetrahydroisoquinoline moiety) at the C-7 position. Quinolone 33 demonstrated the ability to bind TRBP by both SPR and cellular thermal shift analyses, to modulate RNAi enhancing activity by promoting shRNA to siRNA maturation, and to enhance endogenous pre-miRNA processing. Furthermore, the anti-proliferative effect of 33 was also TRBP-dependent in the cell context; indeed, it lost its cytotoxic effect in TRBP KO SK-Hep-1 and HCCLM3 cells. Notably, in the A2780 cancer cell line, this anti-proliferative effect is also accompanied by the induction of the apoptotic process, as evidenced by the activation of PARP1 cleavage and the downregulation of Bcl-X_L_ protein. In addition, compound 33 sensitized A2780 CIS to cisplatin and led to a more effective cisplatin inhibitory effect on A2780 proliferation, suggesting that, by enhancing the biogenesis of specific miRNAs, 33 may impact those cellular processes that promote proliferation and sustain cisplatin resistance. Finally, compound 33 exhibited antiproliferative activity against other cancer types, including breast, lung, and colon cancers, indicating a broader applicability of its anticancer properties.

In conclusion, here, we demonstrate that enoxacin is not the sole member of the quinolone class to exert anticancer activity through the modulation of the miRNA/RNAi machinery (SMER activity). Furthermore, the novel quinolone derivative 33 exhibited enhanced potency and a more robust binding affinity for TRBP compared to enoxacin. These data provide further support for the innovative concept of targeting miRNA maturation by small molecules as a potential OC therapy. We are aware that compound 33 is not yet a viable preclinical candidate and further studies will be conducted to identify more potent and cancer-specific agents capable of interacting with TRBP. However, this represents the first study of medicinal chemistry to identify quinolone agents that act as antitumor agents through an innovative mechanism of action.

## Materials and methods

### Chemistry

Unless otherwise stated, all starting materials were commercially available. Reagents and solvents were purchased from common commercial suppliers and used as received. Organic solutions were dried over anhydrous Na_2_SO_4_ and concentrated under reduced pressure using a Büchi rotary evaporator. All reactions were routinely checked by TLC on silica gel 60_F254_ (Merck) and visualized by UV and iodine. Purifications were performed by flash column chromatography separations on Merck silica gel 60 (mesh 230–400) or BUCHI Reveleris-X2 Flash Chromatography. Yields are based on purified products and are not optimized. ^1^H NMR and ^13^C NMR spectra were recorded at 200 or 400 and 100 MHz, respectively, using Bruker Advance DRX-200 and DRX-400 instruments. Chemical shifts (*δ*) are reported in ppm relative to TMS and calibrated using residual undeuterated solvent as an internal reference. Coupling constants (*J*) are expressed in Hz. Spectra were acquired at 298 K. Data processing was performed using Bruker TopSpin 4.3.0 software, and the spectral data are consistent with the assigned structures. The purity (>95%) of the compounds was evaluated by HPLC analysis using an Agilent 1290 Infinity System instrument equipped with a DAD detector from 190 to 640 nm. Purity was determined at 254 nm using a Phenomenex AERIS Widepore C4, 4.6 mm, 100 mm (6.6 lm) at a flow rate of 0.85 mL min^−1^; acquisition time: 10 min; gradient: acetonitrile in water containing 0.1% formic acid (0–100% in 10 min); oven temperature, 30 °C. Peak retention time is given in minutes. HRMS detection was based on electrospray ionization (ESI) in positive polarity, using an Agilent 1290 Infinity System equipped with an Agilent 6540UHD Accurate Mass Q-TOF MS detector.

#### Ethyl (2*E*/*Z*)-2-(2-chloro-4-fluoro-3-methyl-5-nitrobenzoyl)-3-(ethylamino)acrylate (35)

To a solution of 34 (ref. 33) (1.00 g, 2.78 mmol) in a mixture of Et_2_O (12 mL) and EtOH (32 mL), 2 M EtNH_2_ in THF (2.26 mL, 4.46 mmol) was added dropwise, and the reaction mixture was stirred at rt for 15 min. After evaporating two-thirds of the reaction volume under vacuum, a precipitate was observed and then filtered to give a yellow solid in 95% yield (0.95 g). ^1^H NMR (200 MHz, acetone-*d*_6_): *δ* 0.91 (t, *J* = 7.1 Hz, 3H, NHCH_2_*CH*_3_), 1.29 (t, *J* = 7.2 Hz, 3H, OCH_2_*CH*_3_), 2.36 (d, *J* = 9.4 Hz, 3H, CH_3_), 3.57–3.69 (m, 2H, NH*CH*_2_CH_3_), 3.81 (q, *J* = 7.0 Hz, 2H, O*CH*_2_CH_3_), 7.71 (d, *J* = 6.0 Hz, 1H, CH), 8.23–8.32 (m, 1H, H-6), 11.00 (bs, 1H, NH).

#### Ethyl 1-ethyl-7-fluoro-8-methyl-6-nitro-4-oxo-1,4-dihydroquinoline-3-carboxylate (36)

To a solution of 35 (0.75 g, 2.10 mmol) in DMF (15 mL), K_2_CO_3_ (0.58 g, 4.20 mmol) was added portionwise and the reaction was stirred at 100 °C for 1 h. Then, the mixture was poured into ice/water and the obtained precipitate was filtered to give a yellow solid in 82% yield (0.55 g). ^1^H NMR (200 MHz, DMSO-*d*_6_): *δ* 1.20–1.29 (m, 6H, NCH_2_*CH*_3_ and OCH_2_*CH*_3_), 2.53 (d, *J* = 9.5 Hz, 3H, CH_3_), 4.17 (q, *J* = 7.2 Hz, 2H, O*CH*_2_CH_3_), 4.44 (q, *J* = 7.9 Hz, 2H, N*CH*_2_CH_3_), 8.63–8.71 (m, 2H, H-2 and H-5).

#### General procedure A (acidic hydrolysis)

To a solution of the ester derivative (1 equiv.) in EtOH (3.5 mL per mmol), 6 N HCl (3.5 mL per mmol) was added, and the reaction was stirred at reflux. Then, the mixture was allowed to cool at rt, and the obtained precipitate was filtered to give a solid that was either purified, as described below for each compound, or used as such for the next reaction.

#### 1-Ethyl-7-fluoro-8-methyl-6-nitro-4-oxo-1,4-dihydroquinoline-3-carboxylic acid (37)

General procedure A: starting from 36 (reaction time: 1 h), compound 37 was obtained as a light brown solid in 93% yield (0.42 g). ^1^H NMR (200 MHz, DMSO-*d*_6_): *δ* 1.32 (t, *J* = 7.8 Hz, 3H, NCH_2_*CH*_3_), 2.58 (d, *J* = 10.4 Hz, 3H, CH_3_), 4.60 (q, *J* = 6.4 Hz, 2H, N*CH*_2_CH_3_), 8.80 (d, *J* = 8.5 Hz, 1H, H-5), 8.98 (s, 1H, H-2), 14.14 (bs, 1H, CO_2_H).

#### General procedure B (nucleophilic substitution)

Under N_2_ atmosphere, to a solution of the compound (1 equiv.) in dry DMF or DMSO (7 mL per 1 mmol), the desired nucleophile (3–5 equiv.) and Et_3_N (3 equiv.) were added, and the reaction was stirred at 80–110 °C. Then, the mixture was poured into ice/water, pH adjusted to 3 with 2 N HCl, and the obtained precipitate was filtered to give a solid that was either purified, as described below for each compound, or used as such for the next reaction.

#### 7-(3,4-Dihydroisoquinolin-2(1*H*)-yl)-1-ethyl-8-methyl-6-nitro-4-oxo-1,4-dihydroquinoline-3-carboxylic acid (38)

General procedure B: starting from 37 and using 1,2,3,4-tetrahydroisoquinoline (5 equiv.) as a nucleophile (reaction time: 1 h; temperature: 80 °C; solvent: DMF), compound 38, after crystallization by EtOH/DMF, was obtained as a light brown solid in 32% yield (0.20 g). ^1^H NMR (200 MHz, DMSO-*d*_6_): *δ* 1.27 (t, *J* = 7.1 Hz, 3H, NCH_2_*CH*_3_), 2.49 (s, 3H, CH_3_), 2.78–2.87 (m, 2H, tetrahydroisoquinoline-CH_2_), 3.15–3.25 (m, 2H, tetrahydroisoquinoline-CH_2_), 4.30–4.38 (m, 2H, tetrahydroisoquinoline-CH_2_), 4.51 (q, *J* = 6.6 Hz, 2H, N*CH*_2_CH_3_), 7.08–7.24 (m, 4H, Ar–H), 8.52 (s, 1H, H-2), 8.93 (s, 1H, H-5), 14.42 (s, 1H, CO_2_H).

#### General procedure C (nitro reduction)

To a solution of the nitro-derivative (1 equiv.) in DMF (25 mL per mmol), RANEY®/Ni (10% w/w) was added portionwise and the reaction was stirred at rt under H_2_ flux. Then, the mixture was filtered over Celite™, and the filtrate was evaporated to dryness to give a solid that was either purified, as described below for each compound, or used as such for the next reaction.

#### 6-Amino-7-(3,4-dihydroisoquinolin-2(1*H*)-yl)-1-ethyl-8-methyl-4-oxo-1,4-dihydroquinoline-3-carboxylic acid (11)

General procedure C: starting from 38 (reaction time: 30 min), compound 11, after crystallization by EtOH/DMF, was obtained as a yellow solid in 78% yield (0.18 g). ^1^H NMR (400 MHz, DMSO-*d*_6_): *δ* 1.21 (t, *J* = 7.0 Hz, 3H, NCH_2_*CH*_3_), 2.46 (s, 3H, CH_3_), 2.81–2.84 (m, 1H, tetrahydroisoquinoline-CH_2_ ×½), 2.94–2.97 (m, 1H, tetrahydroisoquinoline-CH_2_ ×½), 3.38–3.42 (m, 2H, tetrahydroisoquinoline-CH_2_), 4.21–4.28 (m, 2H, N*CH*_2_CH_3_), 4.58–4.62 (m, 2H, tetrahydroisoquinoline-CH_2_), 5.32 (bs, 2H, NH_2_), 7.09–7.15 (m, 4H, Ar–H), 7.44 (s, 1H, H-5), 8.69 (s, 1H, H-2), 15.73 (bs, 1H, CO_2_H). ^13^C NMR (100 MHz, DMSO-*d*_6_): *δ* 15.93, 18.99, 30.75, 47.90, 49.60, 52.78, 105.16, 106.84, 125.57, 125.87, 126.07, 126.47, 126.54, 129.64, 133.96, 135.12, 135.25, 143.83, 144.78, 148.66, 167.14, 177.08. HPLC: r_t_ 11.338 min. HRMS (ESI) calculated for C_22_H_23_N_3_O_3_ [M + H]^+^ 378.1812, found 378.1813.

#### 1-Cyclopropyl-7-fluoro-8-methyl-6-nitro-4-oxo-1,4-dihydroquinoline-3-carboxylic acid (40)^33^

General procedure A: starting from 39 (ref. 33) (reaction time: 1 h), compound 40 (ref. 33) was obtained as a light brown solid in 86% yield (1.00 g). ^1^H NMR (200 MHz, DMSO-*d*_6_): *δ* 1.21–1.24 (m, 2H, cyclopropyl-CH_2_), 1.29–1.41 (m, 2H, cyclopropyl-CH_2_), 2.95 (d, *J* = 3.7 Hz, 3H, CH_3_), 4.48–4.53 (m, 1H, CH), 8.87 (d, *J* = 8.4 Hz, 1H, H-5), 8.96 (s, 1H, H-2), 14.18 (bs, 1H, CO_2_H).

#### 1-Cyclopropyl-8-methyl-6-nitro-4-oxo-7-(4-phenylpiperidin-1-yl)-1,4-dihydroquinoline-3-carboxylic acid (41)

General procedure B: starting from 40 (ref. 33) and using 4-phenylpiperidine (4 equiv.) as a nucleophile (reaction time: 2 h; temperature: 100 °C; solvent: DMSO), compound 41, after purification by flash chromatography column eluting with CHCl_3_/MeOH 99 : 1, was obtained as a brown solid in 30% yield (0.30 g). ^1^H NMR (400 MHz, DMSO-*d*_6_): *δ* 0.83–0.86 (m, 2H, cyclopropyl-CH_2_), 1.15–1.20 (m, 2H, cyclopropyl-CH_2_), 1.80–1.85 (m, 4H, piperidine-CH_2_ ×2), 2.71–2.74 (m, 4H, piperidine-CH and CH_3_), 3.11–3.15 (m, 2H, piperidine-NCH_2_), 3.20–3.26 (m, 2H, piperidine-NCH_2_), 4.30–4.35 (m, 1H, CH), 7.17–7.20 (m, 1H, H-4′), 7.27–7.35 (m, 4H, Ar–H), 8.42 (s, 1H, H-2), 8.85 (s, 1H, H-5), 14.41 (bs, 1H, CO_2_H).

#### 1-Cyclopropyl-7-(1,3-dihydro-2*H*-isoindol-2-yl)-8-methyl-6-nitro-4-oxo-1,4-dihydroquinoline-3-carboxylic acid (42)

General procedure B: starting from 40 (ref. 33) and using isoindoline (4 equiv.) as a nucleophile (reaction time: 90 min; temperature: 100 °C; solvent: DMSO), compound 42, after crystallization by EtOH/DMF, was obtained as a brown solid in 74% yield (0.73 g). ^1^H NMR (400 MHz, DMSO-*d*_6_): *δ* 0.90–0.94 (m, 2H, cyclopropyl-CH_2_), 1.18–1.21 (m, 2H, cyclopropyl-CH_2_), 2.69 (s, 3H, CH_3_), 4.32–4.36 (m, 1H, CH), 4.57 (m, 4H, isoindoline-CH_2_ ×2), 7.27–7.31 (m, 2H, Ar–H), 7.32–7.36 (m, 2H, Ar–H), 8.48 (s, 1H, H-2), 8.86 (s, 1H, H-5), 14.83 (bs, 1H, CO_2_H).

#### 6-Amino-1-cyclopropyl-8-methyl-4-oxo-7-(4-phenylpiperidin-1-yl)-1,4-dihydroquinoline-3-carboxylic acid (12)

General procedure C: starting from 41 (reaction time: 12 h), compound 12, after purification by flash chromatography column eluting with CHCl_3_/MeOH 98 : 2, was obtained as a brown solid in 20% yield (0.08 g). ^1^H NMR (400 MHz, DMSO-*d*_6_): *δ* 0.77–0.86 (m, 2H, cyclopropyl-CH_2_), 1.12–1.17 (m, 2H, cyclopropyl-CH_2_), 1.73–1.79 (m, 2H, piperidine-CH_2_), 1.87–1.96 (m, 2H, piperidine-CH_2_), 2.56 (s, 3H, CH_3_), 2.67 (t, *J* = 11.4 Hz, 1H, piperidine-CH), 3.10–3.17 (m, 2H, piperidine-NCH_2_), 3.22–3.37 (m, 2H, piperidine-NCH_2_), 4.25 (s, 1H, CH), 5.40 (s, 2H, NH_2_), 7.17 (t, *J* = 7.1 Hz, 1H, H-4′), 7.28 (t, *J* = 7.4 Hz, 2H, H-3′ and H-5′), 7.31–7.39 (m, 3H, H-5, H-2′ and H-6′), 8.59 (s, 1H, H-2), 15.68 (s, 1H, CO_2_H). ^13^C NMR (100 MHz, DMSO-*d*_6_): *δ* 10.76, 19.24, 34.46, 41.02, 42.48, 49.98, 104.08, 106.43, 124.39, 125.41, 126.44, 127.39, 128.67, 135.70, 144.41, 145.17, 146.93, 148.98, 166.91, 176.96. HPLC: r_t_ 10.398 min. HRMS (ESI) calculated for C_25_H_27_N_3_O_3_ [M + H]^+^ 418.2130, found 418.21288.

#### 6-Amino-1-cyclopropyl-7-(1,3-dihydro-2*H*-isoindol-2-yl)-8-methyl-4-oxo-1,4-dihydroquinoline-3-carboxylic acid (13)

General procedure C: starting from 42 (reaction time: 12 h), compound 13, after purification by chromatography (Reveleris-X2) eluting with CHCl_3_/MeOH 98 : 2 to 95 : 5, was obtained as a brown solid in 14% yield (0.05 g). ^1^H NMR (400 MHz, DMSO-*d*_6_): *δ* 0.85–0.91 (m, 2H, cyclopropyl-CH_2_), 1.13–1.19 (m, 2H, cyclopropyl-CH_2_), 2.53 (s, 3H, CH_3_), 4.18–4.29 (m, 1H, CH), 4.57 (s, 4H, isoindoline-CH_2_ ×2), 5.60 (s, 2H, NH_2_), 7.25–7.28 (m, 2H, Ar–H), 7.31–7.35 (m, 2H, Ar–H), 7.40 (s, 1H, H-5), 8.59 (s, 1H, H-2), 15.60 (s, 1H, CO_2_H). ^13^C NMR (100 MHz, DMSO-*d*_6_): *δ* 10.92, 18.12, 41.30, 55.70, 104.49, 106.14, 123.03, 125.70, 127.17, 128.82, 134.82, 139.60, 140.55, 146.57, 149.02, 166.87, 177.23. HPLC: r_t_ 5.385 min. HRMS (ESI) calculated for C_22_H_21_N_3_O_3_ [M + H]^+^ 376.1661, found 376.16651.

#### Ethyl 1-cyclopropyl-8-methyl-6-nitro-4-oxo-7-[(2-piperidin-1-ylethyl)amino]-1,4-dihydroquinoline-3-carboxylate (43)

General procedure B: starting from 39 (ref. 33) and using 1-(2-aminoethyl)piperidine (3 equiv.) as a nucleophile (reaction time: 2 h; temperature: 100 °C; solvent: DMSO), compound 43, after crystallization by EtOH/DMF, was obtained as an orange solid in 86% yield (0.34 g). ^1^H NMR (400 MHz, DMSO-*d*_6_): *δ* 0.87–0.91 (m, 2H, cyclopropyl-CH_2_), 1.10–1.16 (m, 2H, cyclopropyl-CH_2_), 1.35–1.39 (m, 5H, piperidine-CH_2_ and OCH_2_*CH*_3_), 1.50–1.55 (m, 4H, piperidine-CH_2_ ×2), 2.33–2.36 (m, 4H, piperidine-NCH_2_ ×2), 2.49 (t, *J* = 5.9 Hz, 2H, –*CH*_2_N), 2.55 (s, 3H, CH_3_), 3.30 (q, *J* = 5.5 Hz, 2H, NH*CH*_2_–), 3.86–3.89 (m, 1H, CH), 4.35 (q, 2H, O*CH*_2_CH_3_), 7.74 (t, *J* = 4.3 Hz, 1H, N*H*CH_2_–), 8.42 (s, 1H, H-5), 8.57 (s, 1H, H-2).

#### Ethyl 6-amino-1-cyclopropyl-8-methyl-4-oxo-7-[(2-piperidin-1-ylethyl)amino]-1,4-dihydroquinoline-3-carboxylate (44)

General procedure C: starting from 43 (reaction time: 2 h), compound 44, after purification by flash column chromatography eluting with CHCl_3_/MeOH 95 : 5, was obtained as an orange solid in 43% yield (0.08 g). ^1^H NMR (200 MHz, CDCl_3_): *δ* 0.85–0.88 (m, 2H, cyclopropyl-CH_2_), 1.08–1.13 (m, 2H, cyclopropyl-CH_2_), 1.36–1.59 (m, 9H, piperidine-CH_2_ ×3 and OCH_2_*CH*_3_), 2.38–2.41 (m, 6H, piperidine-NCH_2_ ×2 and NH*CH*_2_–), 2.66 (s, 3H, CH_3_), 3.21 (t, *J* = 5.0 Hz, 2H, –*CH*_2_N), 3.91–3.97 (m, 3H, CH and NH_2_), 4.35 (q, *J* = 7.1 Hz, 2H, O*CH*_2_CH_3_), 7.60 (s, 1H, H-5), 8.58 (s, 1H, H-2).

#### General procedure D (basic hydrolysis)

To a solution of the ester derivative (1 equiv.) in MeOH (5 mL per mmol), a 10% aqueous solution of NaOH was added, and the reaction was stirred at reflux. Then, the mixture was concentrated under vacuum, poured into ice/water, and pH was adjusted with 2 N HCl. The acid derivative was collected by filtration or extraction with EtOAc and then purified as described below for each compound.

#### 6-Amino-1-cyclopropyl-8-methyl-4-oxo-7-[(2-piperidin-1-ylethyl)amino]-1,4-dihydroquinoline-3-carboxylic acid (14)

General procedure D: starting from 44 (reaction time: 1 h; work-up: extraction at pH = 7), compound 14, after crystallization by Et_2_O/EtOH, was obtained as a yellow solid in 65% yield (0.04 g). ^1^H NMR (400 MHz, DMSO-*d*_6_): *δ* 0.75–0.87 (m, 2H, cyclopropyl-CH_2_), 1.11–1.25 (m, 2H, cyclopropyl-CH_2_), 1.31–1.39 (m, 2H, piperidine-CH_2_), 1.41–1.52 (m, 4H, piperidine-CH_2_ ×2), 2.26–2.40 (m, 6H, N*CH*_2_– and piperidine-NCH_2_ ×2), 2.61 (s, 3H, CH_3_), 3.16–3.22 (m, 2H, –*CH*_2_NH), 4.26–4.32 (m, 1H, CH), 4.60 (t, *J* = 6.1 Hz, 1H, NH), 5.55 (s, 2H, NH_2_), 7.33 (s, 1H, H-2), 8.57 (s, 1H, H-5), 15.98 (s, 1H, CO_2_H). ^13^C NMR (100 MHz, DMSO-*d*_6_): *δ* 10.84, 18.37, 24.25, 25.81, 41.15, 44.17, 54.38, 58.61, 104.72, 106.04, 117.52, 121.72, 136.57, 140.97, 144.53, 148.70, 167.30, 176.45. HPLC: r_t_ 2.187 min. HRMS (ESI) calculated for C_21_H_28_N_4_O_3_ [M + H]^+^ 385.2239, found 385.22379.

#### Ethyl 1-cyclopropyl-7-(3,4-dihydroisoquinolin-2(1*H*)-yl)-6-nitro-4-oxo-1,4-dihydroquinoline-3-carboxylate (47)

General procedure B: starting from 45 (ref. 31) and using 1,2,3,4-tetrahydroisoquinoline (5 equiv.) as a nucleophile (reaction time: 90 min; temperature: 100 °C; solvent: DMSO), compound 47 was obtained as a yellow solid in 78% yield (0.61 g). ^1^H NMR (200 MHz, DMSO-*d*_6_): *δ* 0.79–0.95 (m, 2H, cyclopropyl-CH_2_), 0.97–1.10 (m, 5H, cyclopropyl-CH_2_ and OCH_2_*CH*_3_), 2.60–2.80 (m, 2H, tetrahydroisoquinoline-CH_2_), 3.21 (t, *J* = 5.4 Hz, 2H, tetrahydroisoquinoline-CH_2_), 3.30–3.50 (m, 1H, CH), 3.97 (q, *J* = 7.1 Hz, 2H, O*CH*_2_CH_3_), 4.21 (s, 2H, tetrahydroisoquinoline-CH_2_), 6.80–7.05 (m, 4H, Ar–H), 7.26 (s, 1H, H-8), 8.22 (s, 1H, H-2), 8.29 (s, 1H, H-5).

#### Ethyl 1-cyclopropyl-7-(7,8-dihydropyrido[4,3-*d*]pyrimidin-6(5*H*)-yl)-6-nitro-4-oxo-1,4-dihydroquinoline-3-carboxylate (48)

General procedure B: starting from 45 (ref. 31) and using 5,6,7,8-tetrahydropyrido[4,3-*d*]pyrimidine (3 equiv.) as a nucleophile (reaction time: 24 h; temperature: 80 °C; solvent: DMSO), compound 48, after purification by flash chromatography column eluting with CHCl_3_/MeOH 97 : 3, was obtained as a light brown solid in 61% yield (0.57 g). ^1^H NMR (200 MHz, DMSO-*d*_6_): *δ* 0.99–1.15 (m, 2H, cyclopropyl-CH_2_), 1.23–1.27 (m, 5H, cyclopropyl-CH_2_, and OCH_2_*CH*_3_), 3.00 (t, *J* = 5.1 Hz, 2H, 5,6,7,8-tetrahydropyrido[4,3-*d*]pyrimidine-CH_2_), 3.60–3.65 (m, 3H, CH and 5,6,7,8-tetrahydropyrido[4,3-*d*]pyrimidine-CH_2_), 4.16 (q, *J* = 7.1 Hz, 2H, O*CH*_2_CH_3_), 4.47 (s, 2H, 5,6,7,8-tetrahydropyrido[4,3-*d*]pyrimidine-CH_2_), 7.57 (s, 1H, H-8), 8.40 (s, 1H, H-2), 8.51 (s, 1H, H-5), 8.65 (s, 1H, Ar–H), 8.94 (s, 1H, Ar–H).

#### Ethyl 1-cyclopropyl-7-(7,8-dihydropyrido[3,4-*b*]pyrazin-6(5*H*)-yl)-6-nitro-4-oxo-1,4-dihydroquinoline-3-carboxylate (49)

General procedure B: starting from 45 (ref. 31) and using 5,6,7,8-tetrahydropyrido[3,4-*b*]pyrazine (3 equiv.) as a nucleophile (reaction time: 18 h; temperature: 100 °C; solvent: DMF), compound 49, after purification by flash chromatography column eluting with CHCl_3_/MeOH 90 : 10, was obtained as a light brown solid in 78% yield (0.90 g). ^1^H NMR (200 MHz, DMSO-*d*_6_): *δ* 1.00–1.30 (m, 7H, cyclopropyl-CH_2_ ×2 and OCH_2_*CH*_3_), 3.00–3.10 (m, 2H, tetrahydropyrido[3,4-*b*]pyrazine-CH_2_), 3.48–3.70 (m, 3H, tetrahydropyrido[3,4-*b*]pyrazine-CH_2_ and CH), 4.20 (q, *J* = 7.0 Hz, 2H, O*CH*_2_CH_3_), 4.50 (s, 2H, tetrahydropyrido[3,4-*b*]pyrazine-CH_2_), 7.60 (s, 1H, H-8), 8.28–8.55 (m, 4H, H-2, H-5 and Ar–H).

#### Ethyl 1-cyclopropyl-7-(3,4-dihydroquinoxalin-1(2*H*)-yl)-6-nitro-4-oxo-1,4-dihydroquinoline-3-carboxylate (50)

General procedure B: starting from 45 (ref. 31) and using 1,2,3,4-tetrahydroquinoxaline (3 equiv.) as a nucleophile (reaction time: 10 h; temperature: 100 °C; solvent: DMSO), compound 50, after purification by flash chromatography column eluting with CHCl_3_/acetone 98 : 2 to 95 : 5, was obtained as a light brown solid in 34% yield (0.37 g). ^1^H NMR (400 MHz, DMSO-*d*_6_): *δ* 1.06–1.10 (m, 4H, cyclopropyl-CH_2_ ×2), 1.24 (t, *J* = 7.1 Hz, 3H, OCH_2_*CH*_3_), 3.30–3.33 (m, 2H, tetrahydroquinoxaline-CH_2_), 3.53–3.61 (m, 3H, tetrahydroquinoxaline-CH_2_ and cyclopropyl-CH), 4.17 (q, *J* = 7.1 Hz, 2H, O*CH*_2_CH_3_), 6.04 (s, 1H, NH), 6.31 (t, *J* = 7.8 Hz, 1H, Ar–H), 6.55–6.60 (m, 2H, Ar–H), 6.68 (t, *J* = 7.5 Hz, 1H, Ar–H), 7.82 (s, 1H, H-8), 8.42 (s, 1H, H-2), 8.52 (s, 1H, H-5).

#### Ethyl 1-cyclopropyl-7-(6,7-dihydrothieno[3,2-*c*]pyridin-5(4*H*)-yl)-6-nitro-4-oxo-1,4-dihydroquinoline-3-carboxylate (51)

General procedure B: starting from 45 (ref. 31) and using 4,5,6,7-tetrahydrothieno[3,2-*c*]pyridine (3 equiv.) as a nucleophile (reaction time: 3 h; temperature: 100 °C; solvent: DMSO), compound 51 was obtained as a yellow solid in 94% yield (1.14 g). ^1^H NMR (400 MHz, DMSO-*d*_6_): *δ* 1.05–1.09 (m, 2H, cyclopropyl-CH_2_), 1.21–1.29 (m, 5H, cyclopropyl-CH_2_ and OCH_2_*CH*_3_), 2.92–2.96 (m, 2H, tetrahydrothieno[3,2-*c*]pyridine-CH_2_), 3.53 (t, *J* = 5.2 Hz, 2H, tetrahydrothieno[3,2-*c*]pyridine-CH_2_), 3.58–3.62 (m, 1H, CH), 4.17 (q, *J* = 7.1 Hz, 2H, O*CH*_2_CH_3_), 4.31–4.35 (m, 2H, tetrahydrothieno[3,2-*c*]pyridine-CH_2_), 6.91 (d, *J* = 5.2 Hz, 1H, Ar–H), 7.35 (d, *J* = 5.1 Hz, 1H, Ar–H), 7.53 (s, 1H, H-8), 8.40 (s, 1H, H-2), 8.49 (s, 1H, H-5).

#### Ethyl 1-cyclopropyl-7-(1-methyl-3,4-dihydroisoquinolin-2(1*H*)-yl)-6-nitro-4-oxo-1,4-dihydroquinoline-3-carboxylate (52)

General procedure B: starting from 45 (ref. 31) and using 1-methyl-1,2,3,4-tetrahydroisoquinoline (3 equiv.) as a nucleophile (reaction time: 4 h; temperature: 110 °C; solvent: DMSO), compound 52 was obtained as a brown solid in 72% yield (0.48 g). ^1^H NMR (400 MHz, DMSO-*d*_6_): *δ* 0.93–0.96 (m, 2H, cyclopropyl-CH_2_), 1.11–1.23 (m, 5H, cyclopropyl-CH_2_ and OCH_2_*CH*_3_), 1.47 (d, *J* = 15.0 Hz, 3H, CH_3_), 2.75–2.81 (m, 1H, tetrahydroisoquinoline-CH_2_ ×½), 2.93–2.97 (m, 1H, tetrahydroisoquinoline-CH_2_ ×½), 3.36–3.41 (m, 1H, tetrahydroisoquinoline-CH_2_ ×½), 3.58–3.62 (m, 2H, cyclopropyl-CH and tetrahydroisoquinoline-CH_2_ ×½), 4.17 (q, *J* = 7.0 Hz, 2H, O*CH*_2_CH_3_), 4.78 (q, *J* = 6.3 Hz, 1H, tetrahydroisoquinoline-NCH), 7.15–7.23 (m, 4H, Ar–H), 7.58 (s, 1H, H-8), 8.39 (s, 1H, H-2), 8.43 (s, 1H, H-5).

#### Ethyl 1-cyclopropyl-7-[(3*S*)-3-(hydroxymethyl)-3,4-dihydroisoquinolin-2(1*H*)-yl]-6-nitro-4-oxo-1,4-dihydroquinoline-3-carboxylate (53)

General procedure B: starting from 45 (ref. 31) and using (3*S*)-1,2,3,4-tetrahydroisoquinolin-3-ylmethanol (3 equiv.) as a nucleophile (reaction time: 34 h; temperature: 110 °C; solvent: DMSO), compound 57, after purification by flash chromatography column eluting with CHCl_3_/MeOH 99 : 1 to 98 : 2, was obtained as a yellow solid in 69% yield (0.48 g). ^1^H NMR (400 MHz, DMSO-*d*_6_): *δ* 1.05–1.09 (m, 2H, cyclopropyl-CH_2_), 1.19–1.28 (m, 5H, cyclopropyl-CH_2_ and OCH_2_*CH*_3_), 2.71–2.73 (m, 1H, tetrahydroisoquinoline-CH), 3.16–3.21 (m, 1H, ½ *CH*_2_OH), 3.42–3.46 (m, 2H, ½ *CH*_2_OH and cyclopropyl-CH), 3.59–3.63 (m, 2H, tetrahydroisoquinoline-CH_2_), 4.18 (q, *J* = 7.1 Hz, 2H, O*CH*_2_CH_3_), 4.31 (d, *J* = 16.2 Hz, 1H, tetrahydroisoquinoline-CH_2_ ×½), 4.62 (d, *J* = 16.1 Hz, 1H, tetrahydroisoquinoline-CH_2_ ×½), 4.75 (t, *J* = 5.2 Hz, 1H, OH), 7.18–7.23 (m, 4H, Ar–H), 7.58 (s, 1H, H-8), 8.42 (s, 1H, H-2), 8.49 (s, 1H, H-5).

#### Ethyl 1-cyclopropyl-6-nitro-4-oxo-7-(4-phenylpiperidin-1-yl)-1,4-dihydroquinoline-3-carboxylate (54)

General procedure B: starting from 45 (ref. 31) and using 4-phenylpiperidine (3 equiv.) as a nucleophile (reaction time: 2 h; temperature: 100 °C; solvent: DMSO), compound 54 was obtained as a yellow solid in 90% yield (0.62 g). ^1^H NMR (400 MHz, DMSO-*d*_6_): *δ* 1.07–1.11 (m, 2H, cyclopropyl-CH_2_), 1.18–1.28 (m, 5H, cyclopropyl-CH_2_ and OCH_2_*CH*_3_), 1.73–1.88 (m, 4H, piperidine-CH_2_ ×2), 2.71–2.73 (m, 1H, piperidine-CH), 3.08 (t, *J* = 10.9 Hz, 2H, piperidine-NCH_2_), 3.46 (d, *J* = 13.5 Hz, 2H, piperidine-NCH_2_), 3.60–3.63 (m, 1H, CH), 4.18 (q, *J* = 7.1 Hz, 2H, O*CH*_2_CH_3_), 7.15–7.20 (m, 1H, Ar–H), 7.25–7.31 (m, 4H, Ar–H), 7.50 (s, 1H, H-8), 8.41 (s, 1H, H-2), 8.48 (s, 1H, H-5).

#### Ethyl 7-[benzyl(ethyl)amino]-1-cyclopropyl-6-nitro-4-oxo-1,4-dihydroquinoline-3-carboxylate (55)

General procedure B: starting from 45 (ref. 31) and using *N*-benzylethanamine (3 equiv.) as a nucleophile (reaction time: 48 h; temperature: 100 °C; solvent: DMSO), compound 55 was obtained as a yellow solid in 90% yield (0.90 g). ^1^H NMR (400 MHz, DMSO-*d*_6_): *δ* 0.78–0.82 (m, 2H, cyclopropyl-CH_2_), 1.03–1.04 (m, 2H, cyclopropyl-CH_2_), 1.12 (t, *J* = 6.3 Hz, 3H, NCH_2_*CH*_3_), 1.21 (t, *J* = 6.8 Hz, 3H, OCH_2_*CH*_3_), 3.22 (q, *J* = 6.7 Hz, 2H, N*CH*_2_CH_3_), 3.43–3.46 (m, 1H, CH), 4.15 (q, *J* = 6.8 Hz, 2H, O*CH*_2_CH_3_), 4.56 (s, 2H, CH_2_), 7.19–7.34 (m, 6H, Ar–H), 8.33 (s, 1H, H-2), 8.37 (s, 1H, H-5).

#### Ethyl 7-[benzyl(2-hydroxyethyl)amino]-1-cyclopropyl-6-nitro-4-oxo-1,4-dihydroquinoline-3-carboxylate (56)

General procedure B: starting from 45 (ref. 31) and using 2-(benzylamino)ethanol (3 equiv.) as a nucleophile (reaction time: 24 h; temperature: 100 °C; solvent: DMSO), compound 56, after crystallization by EtOH/DMF, was obtained as a yellow solid in 50% yield (0.65 g). ^1^H NMR (400 MHz, DMSO-*d*_6_): *δ* 0.77–0.81 (m, 2H, cyclopropyl-CH_2_), 1.04–1.08 (m, 2H, cyclopropyl-CH_2_), 1.22 (t, *J* = 7.1 Hz, 3H, OCH_2_*CH*_3_), 3.24 (t, *J* = 5.7 Hz, 2H, N*CH*_2_CH_2_OH), 3.41–3.45 (m, 1H, CH), 3.57 (q, *J* = 5.4 Hz, 2H, NCH_2_*CH*_2_OH), 4.16 (q, *J* = 7.1 Hz, 2H, O*CH*_2_CH_3_), 4.68–4.72 (m, 2H, CH and OH), 7.18–7.22 (m, 1H, Ar–H), 7.27–7.31 (m, 2H, Ar–H), 7.36–7.37 (m, 2H, Ar–H), 7.41 (s, 1H, H-8), 8.34 (s, 1H, H-2), 8.38 (s, 1H, H-5).

#### Ethyl 6-amino-1-cyclopropyl-7-(3,4-dihydroisoquinolin-2(1*H*)-yl)-4-oxo-1,4-dihydroquinoline-3-carboxylate (57)

General procedure C: starting from 47 (reaction time: 90 min), compound 57, after crystallization by EtOH, was obtained as a white solid in 85% yield (0.48 g). ^1^H NMR (400 MHz, DMSO-*d*_6_): *δ* 0.91–0.97 (m, 2H, cyclopropyl-CH_2_), 1.03–1.15 (m, 2H, cyclopropyl-CH_2_), 1.24 (t, *J* = 7.0 Hz, 3H, OCH_2_*CH*_3_), 2.91–3.02 (m, 2H, tetrahydroisoquinoline-CH_2_), 3.23–3.30 (m, 2H, tetrahydroisoquinoline-CH_2_), 3.45–3.55 (m, 1H, CH), 4.14 (q, *J* = 7.0 Hz, 2H, O*CH*_2_CH_3_), 4.22 (s, 2H, tetrahydroisoquinoline-CH_2_), 5.16 (s, 2H, NH_2_), 7.09–7.22 (m, 4H, Ar–H), 7.40 (s, 1H, H-8), 7.50 (s, 1H, H-5), 8.25 (s, 1H, H-2).

#### Ethyl 6-amino-1-cyclopropyl-7-(7,8-dihydropyrido[4,3-*d*]pyrimidin-6(5*H*)-yl)-4-oxo-1,4-dihydroquinoline-3-carboxylate (58)

General procedure C: starting from 48 (reaction time: 3 h), compound 58, after crystallization by Et_2_O/EtOH, was obtained as a white solid in 66% yield (0.24 g). ^1^H NMR (200 MHz, DMSO-*d*_6_): *δ* 0.85–0.99 (m, 2H, cyclopropyl-CH_2_), 1.00–1.23 (m, 5H, cyclopropyl-CH_2_ and OCH_2_*CH*_3_), 2.90–3.10 (m, 2H, tetrahydropyrido[4,3-*d*]pyrimidine-CH_2_), 3.30–3.40 (m, 2H, tetrahydropyrido[4,3-*d*]pyrimidine-CH_2_), 3.50–3.60 (m, 1H, CH), 4.00–4.35 (m, 4H, O*CH*_2_CH_3_ and tetrahydropyrido[4,3-*d*]pyrimidine-CH_2_), 5.27 (s, 2H, NH_2_), 7.44 (s, 1H, H-8), 7.48 (s, 1H, H-5), 8.26 (s, 1H, H-2), 8.60 (s, 1H, Ar–H), 8.93 (s, 1H, Ar–H).

#### Ethyl 6-amino-1-cyclopropyl-7-(7,8-dihydropyrido[3,4-*b*]pyrazin-6(5*H*)-yl)-4-oxo-1,4-dihydroquinoline-3-carboxylate (59)

General procedure C: starting from 49 (reaction time: 2 h), compound 59, after purification by flash chromatography column eluting with CHCl_3_/MeOH 94 : 6, was obtained as a whitish solid in 40% yield (0.12 g). ^1^H NMR (200 MHz, DMSO-*d*_6_): *δ* 0.90–1.05 (m, 2H, cyclopropyl-CH_2_), 1.07–1.27 (m, 5H, cyclopropyl-CH_2_ and OCH_2_*CH*_3_), 3.00–3.15 (m, 2H, tetrahydropyrido[3,4-*b*]pyrazine-CH_2_), 3.39 (t, *J* = 5.3 Hz, 2H, tetrahydropyrido[3,4-*b*]pyrazine-CH_2_), 3.45–3.55 (m, 1H, CH), 4.12 (q, *J* = 7.1 Hz, 2H, O*CH*_2_CH_3_), 4.29 (s, 2H, tetrahydropyrido[3,4-*b*]pyrazine-CH_2_), 5.23 (s, 2H, NH_2_), 7.45 (s, 1H, H-8), 7.49 (s, 1H, H-5), 8.25 (s, 1H, H-2), 8.45 (d, *J* = 2.5 Hz, 1H, Ar–H), 8.49 (d, *J* = 2.5 Hz, 1H, Ar–H).

#### Ethyl 6-amino-1-cyclopropyl-7-(3,4-dihydroquinoxalin-1(2*H*)-yl)-4-oxo-1,4-dihydroquinoline-3-carboxylate (60)

General procedure C: starting from 50 (reaction time: 4 h), compound 60, after purification by flash chromatography column eluting with CHCl_3_/MeOH 99 : 1, was obtained as a whitish solid in 32% yield (0.06 g). ^1^H NMR (400 MHz, DMSO-*d*_6_): *δ* 0.94–1.06 (m, 4H, cyclopropyl-CH_2_ ×2), 1.22 (t, *J* = 7.0 Hz, 3H, OCH_2_*CH*_3_), 3.38–3.41 (m, 2H, dihydroquinoxaline-CH_2_), 3.47–3.50 (m, 3H, dihydroquinoxaline-CH_2_ and cyclopropyl-CH), 4.15 (q, *J* = 6.9 Hz, 2H, O*CH*_2_CH_3_), 5.17 (s, 2H, NH_2_), 5.74 (s, 1H, NH), 6.09 (d, *J* = 7.8 Hz, 1H, Ar–H), 6.29 (t, *J* = 5.5 Hz, 1H, Ar–H), 6.50–6.55 (m, 2H, Ar–H), 7.49 (s, 1H, H-5), 7.55 (s, 1H, H-8), 8.28 (s, 1H, H-2).

#### Ethyl 6-amino-1-cyclopropyl-7-(1-methyl-3,4-dihydroisoquinolin-2(1*H*)-yl)-4-oxo-1,4-dihydroquinoline-3-carboxylate (61)

General procedure C: starting from 52 (reaction time: 1 h), compound 61, after crystallization by EtOH, was obtained as a brown solid in 75% yield (0.35 g). ^1^H NMR (400 MHz, DMSO-*d*_6_): *δ* 0.73–0.82 (m, 2H, cyclopropyl-CH_2_), 0.93–1.05 (m, 2H, cyclopropyl-CH_2_), 1.22 (t, *J* = 7.2 Hz, 3H, OCH_2_*CH*_3_), 1.28 (d, *J* = 6.5 Hz, 3H, CH_3_), 2.73–2.79 (m, 2H, tetrahydroisoquinoline-CH_2_), 3.25–3.27 (m, 1H, tetrahydroisoquinoline-CH_2_ ×½), 3.35–3.38 (m, 1H, tetrahydroisoquinoline-CH_2_ ×½), 3.46–3.49 (m, 1H, CH), 4.14 (q, *J* = 7.0 Hz, 2H, O*CH*_2_CH_3_), 4.65 (q, *J* = 6.4 Hz, 1H, tetrahydroisoquinoline-CH), 5.19 (s, 2H, NH_2_), 7.09–7.23 (m, 4H, Ar–H), 7.38 (s, 1H, H-5), 7.43 (s, 1H, H-8), 8.23 (s, 1H, H-2).

#### Ethyl 6-amino-1-cyclopropyl-7-[(3*S*)-3-(hydroxymethyl)-3,4-dihydroisoquinolin-2(1*H*)-yl]-4-oxo-1,4-dihydroquinoline-3-carboxylate (62)

General procedure C: starting from 53 (reaction time: 2 h), compound 62 was obtained as a brown solid in 90% yield (0.42 g). ^1^H NMR (400 MHz, DMSO-*d*_6_): *δ* 0.71–0.78 (m, 2H, cyclopropyl-CH_2_), 0.86–1.01 (m, 2H, cyclopropyl-CH_2_), 1.21 (t, *J* = 6.6 Hz, 3H, OCH_2_*CH*_3_), 2.61–2.66 (m, 1H, tetrahydroisoquinoline-CH_2_ ×½), 2.97–3.00 (m, 1H, tetrahydroisoquinoline-CH_2_ ×½), 3.82–3.89 (m, 2H, tetrahydroisoquinoline-CH and cyclopropyl-CH), 3.55–3.58 (m, 1H, CH_2_OH ×½), 3.71–3.75 (m, 1H, CH_2_OH ×½), 4.08–4.14 (m, 3H, O*CH*_2_CH_3_ and tetrahydroisoquinoline-CH_2_ ×½), 4.52–4.56 (m, 1H, tetrahydroisoquinoline-CH_2_ ×½), 4.83 (bs, 1H, OH), 5.26 (s, 2H, NH_2_), 7.07–7.21 (m, 4H, Ar–H), 7.37–7.41 (m, 2H, H-5 and H-8), 8.21 (s, 1H, H-2).

#### Ethyl 6-amino-1-cyclopropyl-4-oxo-7-(4-phenylpiperidin-1-yl)-1,4-dihydroquinoline-3-carboxylate (63)

General procedure C: starting from 54 (reaction time: 2 h), compound 63, after crystallization by EtOH, was obtained as a white solid in 72% yield (0.37 g). ^1^H NMR (400 MHz, DMSO-*d*_6_): *δ* 1.02–1.04 (m, 2H, cyclopropyl-CH_2_), 1.17–1.25 (m, 5H, cyclopropyl-CH_2_ and OCH_2_*CH*_3_), 1.84–1.99 (m, 4H, piperidine-CH_2_ ×2), 2.65–2.73 (m, 3H, piperidine-NCH_2_ and -CH), 3.39–3.42 (m, 2H, piperidine-NCH_2_), 3.55–3.59 (m, 1H, CH), 4.15 (q, *J* = 7.1 Hz, 2H, O*CH*_2_CH_3_), 5.14 (s, 2H, NH_2_), 7.15–7.20 (m, 1H, Ar–H), 7.29–7.30 (m, 4H, Ar–H), 7.43 (s, 1H, H-5), 7.45 (s, 1H, H-8), 8.28 (s, 1H, H-2).

#### Ethyl 6-amino-7-[benzyl(ethyl)amino]-1-cyclopropyl-4-oxo-1,4-dihydroquinoline-3-carboxylate (64)

General procedure C: starting from 55 (reaction time: 90 min), compound 64 was obtained as an orange solid in 57% yield (0.20 g). ^1^H NMR (400 MHz, DMSO-*d*_6_): *δ* 0.80–0.86 (m, 2H, cyclopropyl-CH_2_), 1.02 (t, *J* = 6.9 Hz, 3H, NCH_2_*CH*_3_), 1.12–1.17 (m, 2H, cyclopropyl-CH_2_), 1.21 (t, *J* = 7.1 Hz, 3H, OCH_2_*CH*_3_), 3.05 (q, *J* = 7.1 Hz, 2H, N*CH*_2_CH_3_), 3.44–3.46 (m, 1H, CH), 4.13 (q, *J* = 7.1 Hz, 2H, O*CH*_2_CH_3_), 4.25 (s, 2H, CH_2_), 5.23 (s, 2H, NH_2_), 7.12–7.17 (m, 1H, Ar–H), 7.20–7.24 (m, 2H, Ar–H), 7.29–7.31 (m, 2H, Ar–H), 7.35 (s, 1H, H-5), 7.40 (s, 1H, H-8), 8.23 (s, 1H, H-2).

#### Ethyl 6-amino-7-[benzyl(2-hydroxyethyl)amino]-1-cyclopropyl-4-oxo-1,4-dihydroquinoline-3-carboxylate (65)

General procedure C: starting from 56 (reaction time: 1 h), compound 65 after crystallization by EtOH, was obtained as a yellow solid in 53% yield (0.32 g). ^1^H NMR (400 MHz, DMSO-*d*_6_): *δ* 0.72–0.77 (m, 2H, cyclopropyl-CH_2_), 0.99–1.04 (m, 2H, cyclopropyl-CH_2_), 1.20 (t, *J* = 7.1 Hz, 3H, OCH_2_*CH*_3_), 3.07 (t, *J* = 5.3 Hz, 2H, N*CH*_2_CH_2_OH), 3.39–3.43 (m, 1H, CH), 3.62 (q, *J* = 5.1 Hz, 2H, NCH_2_*CH*_2_OH), 4.12 (q, *J* = 7.1 Hz, 2H, O*CH*_2_CH_3_), 4.36 (s, 2H, CH_2_), 4.92 (t, *J* = 4.9 Hz, 1H, OH), 5.55 (s, 2H, NH_2_), 7.09–7.13 (m, 1H, Ar–H), 7.17–7.22 (m, 2H, Ar–H), 7.28–7.30 (m, 2H, Ar–H), 7.34 (s, 1H, H-5), 7.35 (s, 1H, H-8), 8.19 (s, 1H, H-2).

#### 6-Amino-1-cyclopropyl-7-(3,4-dihydroisoquinolin-2(1*H*)-yl)-4-oxo-1,4-dihydroquinoline-3-carboxylic acid (15)

General procedure A: starting from 57 (reaction time: 9 h), compound 15, after crystallization by EtOH/DMF, was obtained as a pale yellow solid in 82% yield (0.30 g). ^1^H NMR (400 MHz, DMSO-*d*_6_): *δ* 0.98–1.04 (m, 2H, cyclopropyl-CH_2_), 1.15–1.25 (m, 2H, cyclopropyl-CH_2_), 2.90–3.00 (m, 2H, tetrahydroisoquinoline-CH_2_), 3.36 (t, *J* = 5.4 Hz, 2H, tetrahydroisoquinoline-CH_2_), 3.65–3.75 (m, 1H, CH), 4.29 (s, 2H, tetrahydroisoquinoline-CH_2_), 5.44 (s, 2H, NH_2_), 7.10–7.19 (m, 4H, Ar–H), 7.50 (s, 1H, H-8), 7.55 (s, 1H, H-5), 8.48 (s, 1H, H-2), 15.98 (s, 1H, CO_2_H). ^13^C NMR (100 MHz, DMSO-*d*_6_): *δ* 7.76, 28.56, 36.22, 47.85, 52.59, 106.25, 106.95, 107.87, 122.03, 126.25, 126.81 (2C), 129.17, 134.29, 134.53, 134.67, 142.60, 145.02, 145.71, 167.22, 176.73. HPLC: r_t_ 5.4300 min. HRMS-ESI *m*/*z* [M + H]^+^ calcd. for C_22_H_21_N_3_O_3_: 376.1661, found: 376.1657.

#### 6-Amino-1-cyclopropyl-7-(7,8-dihydropyrido[4,3-*d*]pyrimidin-6(5*H*)-yl)-4-oxo-1,4-dihydroquinoline-3-carboxylic acid (18)

General procedure D: starting from 58 (reaction time: 1 h; work-up: filtration at pH = 5), compound 18, after purification by flash column chromatography eluting with CHCl_3_/MeOH 93 : 7, was obtained as a pale yellow solid in 80% yield (0.14 g). ^1^H NMR (400 MHz, DMSO-*d*_6_): *δ* 1.00–1.30 (m, 4H, cyclopropyl-CH_2_ ×2), 2.98–3.18 (m, 2H, tetrahydropyrido[4,3-*d*]pyrimidine-CH_2_), 3.40–3.50 (m, 2H, tetrahydropyrido[4,3-*d*]pyrimidine-CH_2_), 3.70–3.78 (m, 1H, CH), 4.29 (s, 2H, tetrahydropyrido[4,3-*d*]pyrimidine-CH_2_), 5.50 (s, 2H, NH_2_), 7.50 (s, 1H, H-8), 7.62 (s, 1H, H-5), 8.50 (s, 1H, H-2), 8.60 (s, 1H, Ar–H), 9.00 (s, 1H, Ar–H), 15.00 (s, 1H, CO_2_H). ^13^C NMR (100 MHz, DMSO-*d*_6_): *δ* 7.84, 31.71, 35.99, 47.31, 49.10, 106.54, 107.36, 108.21, 122.61, 128.70, 134.26, 142.63, 145.18, 145.20, 155.08, 156.95, 163.63, 167.05, 176.92. HPLC r_t_: 3.5000 min. HRMS-ESI *m*/*z* [M + H]^+^ calcd. for C_20_H_19_N_5_O_3_, 378.1566, found: 378.1560.

#### 6-Amino-1-cyclopropyl-7-(7,8-dihydropyrido[3,4-*b*]pyrazin-6(5*H*)-yl)-4-oxo-1,4-dihydroquinoline-3-carboxylic acid (19)

General procedure D: starting from 59 (reaction time: 90 min; work-up: filtration at pH = 5), compound 19, after crystallization by EtOH/DMF, was obtained as a pale yellow solid in 93% yield (0.09 g). ^1^H NMR (400 MHz, DMSO-*d*_6_): *δ* 1.00–1.30 (m, 4H, cyclopropyl-CH_2_ ×2), 3.19 (t, *J* = 5.3 Hz, 2H, tetrahydropyrido[3,4-*b*]pyrazine-CH_2_), 3.50 (t, *J* = 5.3 Hz, 2H, tetrahydropyrido[3,4-*b*]pyrazine-CH_2_), 3.70–3.80 (m, 1H, CH), 4.41 (s, 2H, tetrahydropyrido[3,4-*b*]pyrazine-CH_2_), 5.56 (s, 2H, NH_2_), 7.57 (s, 1H, H-8), 7.68 (s, 1H, H-5), 8.45–8.52 (m, 3H, Ar–H), 15.86 (s, 1H, CO_2_H). ^13^C NMR (100 MHz, DMSO-*d*_6_): *δ* 7.83, 31.33, 36.06, 47.39, 53.89, 106.25, 107.13, 108.05, 122.31, 134.17, 142.28, 142.65, 143.28, 144.83, 145.18, 150.34, 151.20, 167.20, 176.76. HPLC r_t_: 3.7700 min. HRMS-ESI *m*/*z* [M + H]^+^ calcd for C_20_H_19_N_5_O_3_, 378.1566, found: 378.1562.

#### 6-Amino-1-cyclopropyl-7-(3,4-dihydroquinoxalin-1(2*H*)-yl)-4-oxo-1,4-dihydroquinoline-3-carboxylic acid (20)

General procedure D: starting from 60 (reaction time: 2 h; work-up: extraction at pH = 3), compound 20, after crystallization by EtOH/DMF, was obtained as a yellow solid in 25% yield (0.06 g). ^1^H NMR (400 MHz, DMSO-*d*_6_): *δ* 0.97–1.12 (m, 4H, cyclopropyl-CH_2_ ×2), 3.37–3.41 (m, 2H, dihydroquinoxaline CH_2_), 3.51–3.58 (s, 2H, dihydroquinoxaline CH_2_), 3.63–3.69 (m, 1H, CH), 5.52 (s, 2H, NH_2_), 5.84 (s, 1H, NH), 6.16 (d, *J* = 7.7 Hz, 1H, Ar–H), 6.32 (t, *J* = 6.8 Hz, 1H, Ar–H), 6.53 (d, *J* = 6.5 Hz, 1H, Ar–H), 6.59 (t, *J* = 7.4 Hz, 1H, Ar-H), 7.55 (s, 1H, H-5), 7.76 (s, 1H, H-8), 8.47 (s, 1H, H-2), 15.74 (s, 1H, CO_2_H). ^13^C NMR (100 MHz, DMSO-*d*_6_): *δ* 7.76, 35.96, 47.51 (2C), 106.09, 107.26, 114.40, 114.63, 116.37, 116.80, 121.28, 123.44, 130.27, 133.62, 137.12, 140.97, 143.90, 145.32, 167.06, 176.86. HPLC: r_t_ 5.782 min. HRMS (ESI) calculated for C_21_H_20_N_4_O_3_ [M + H]^+^ 377.1613, found 377.16151.

#### 6-Amino-1-cyclopropyl-7-(6,7-dihydrothieno[3,2-*c*]pyridin-5(4*H*)-yl)-4-oxo-1,4-dihydroquinoline-3-carboxylic acid (21)

Under N_2_ atmosphere, to a solution of 51 (0.22 g, 0.49 mmol) in EtOH (20 mL), SnCl_2_ ×2 H_2_O (0.55 g, 2.45 mmol) was added portionwise and the reaction was stirred at reflux for 2 h. Then, EtOH was evaporated under vacuum and the resulting mixture was poured into ice/water to give a precipitate that was collected by filtration. After purification by flash chromatography column eluting with CHCl_3_/MeOH 99 : 1, compound 21 was obtained as a yellow solid in 21% yield. ^1^H NMR (400 MHz, DMSO-*d*_6_): *δ* 1.03–1.09 (m, 2H, cyclopropyl-CH_2_), 1.12–1.18 (m, 2H, cyclopropyl-CH_2_), 2.91–2.97 (m, 2H, dihydrothienopyridine CH_2_), 3.39 (t, *J* = 5.4 Hz, 2H, dihydrothienopyridine CH_2_), 3.61–3.75 (m, 1H, CH), 4.23 (s, 2H, dihydrothienopyridine CH_2_), 5.45 (s, 2H, NH_2_), 6.92 (d, *J* = 5.1 Hz, 1H, Ar–H), 7.34 (d, *J* = 5.1 Hz, 1H, Ar–H), 7.52 (s, 1H, H-5), 7.60 (s, 1H, H-8), 8.46 (s, 1H, H-2), 15.86 (s, 1H, CO_2_H). ^13^C NMR (100 MHz, DMSO-*d*_6_): *δ* 7.71, 25.06, 35.91, 47.72, 50.09, 106.17, 106.98, 107.97, 121.98, 123.74, 125.76, 133.48, 133.62, 134.21, 142.58, 145.00, 145.36, 167.16, 176.66. HPLC: r_t_ 4.900 min. HRMS (ESI) calculated for C_20_H_19_N_3_O_3_S [M + H]^+^ 382.1225, found 382.12217.

#### 6-Amino-1-cyclopropyl-7-(1-methyl-3,4-dihydroisoquinolin-2(1*H*)-yl)-4-oxo-1,4-dihydroquinoline-3-carboxylic acid (22)

General procedure D: starting from 61 (reaction time: 2 h; work-up: extraction at pH = 3), compound 22, after crystallization by EtOH/DMF, was obtained as a yellow solid in 63% yield (0.18 g). ^1^H NMR (400 MHz, DMSO-*d*_6_): *δ* 0.81–0.87 (m, 2H, cyclopropyl-CH_2_), 0.99–1.15 (m, 2H, cyclopropyl-CH_2_), 1.31 (d, *J* = 6.7 Hz, 3H, CH_3_), 2.78 (t, *J* = 7.0 Hz, 2H, tetrahydroisoquinoline-CH_2_), 3.31–3.40 (m, 1H, CH), 3.53–3.61 (m, 2H, tetrahydroisoquinoline-CH_2_), 4.73 (q, *J* = 6.6 Hz, 1H, tetrahydroisoquinoline-NCH), 5.44 (s, 2H, NH_2_), 7.11 (t, *J* = 8.9 Hz, 1H, Ar–H), 7.14–7.21 (m, 2H, Ar–H), 7.21–7.26 (m, 1H, Ar–H), 7.50 (s, 1H, H-5), 7.53 (s, 1H, H-8), 8.43 (s, 1H, H-2), 15.83 (s, 1H, CO_2_H). ^13^C NMR (100 MHz, DMSO-*d*_6_): *δ* 7.54, 21.38, 27.55, 35.75, 42.28, 54.14, 106.12, 106.69, 110.43, 122.13, 126.30, 126.53, 127.26, 129.19, 133.91, 134.10, 139.85, 143.03, 144.87, 145.02, 167.14, 176.71. HPLC: r_t_ 9.269 min. HRMS (ESI) calculated for C_23_H_23_N_3_O_3_ [M + H]^+^ 390.1817, found 390.18118.

#### 6-Amino-1-cyclopropyl-7-[(3*S*)-3-(hydroxymethyl)-3,4-dihydroisoquinolin-2(1*H*)-yl]-4-oxo-1,4-dihydroquinoline-3-carboxylic acid (23)

General procedure D: starting from 62 (reaction time: 2 h; work-up: extraction at pH = 3), compound 23, after crystallization by EtOH/DMF, was obtained as a yellow solid in 21% yield (0.06 g). ^1^H NMR (400 MHz, DMSO-*d*_6_): *δ* 0.77–0.86 (m, 2H, cyclopropyl-CH_2_), 0.97–1.15 (m, 2H, cyclopropyl-CH_2_), 2.65 (d, *J* = 16.9 Hz, 1H, tetrahydroisoquinoline-CH_2_ ×½), 3.04 (dd, *J* = 17.0, 6.4 Hz, 1H, tetrahydroisoquinoline-CH_2_ ×½), 3.36–3.47 (m, 1H, tetrahydroisoquinoline-CH), 3.49–3.66 (m, 2H, tetrahydroisoquinoline-NCH_2_), 3.81–3.87 (m, 1H, CH), 4.17 (d, *J* = 16.8 Hz, 1H, *CH*_2_OH ×½), 4.60 (d, *J* = 16.8 Hz, 1H, *CH*_2_OH ×½), 4.89 (t, *J* = 5.4 Hz, 1H, OH), 5.56 (s, 2H, NH_2_), 7.07–7.24 (m, 4H, Ar–H), 7.48 (s, 1H, H-5), 7.52 (s, 1H, H-8), 8.41 (s, 1H, H-2), 15.88 (s, 1H, CO_2_H). ^13^C NMR (100 MHz, DMSO-*d*_6_): *δ* 7.48, 7.59, 27.59, 35.71, 47.62, 54.79, 60.57, 106.08, 106.65, 109.23, 121.76, 126.24, 126.33, 126.86, 129.61, 133.56, 134.01, 134.07, 142.72, 144.92, 145.36, 167.18, 176.58. HPLC: r_t_ 7.109 min. HRMS (ESI) calculated for C_23_H_23_N_3_O_4_ [M + H]^+^ 406.1767, found 406.17645.

#### 6-Amino-1-cyclopropyl-4-oxo-7-(4-phenylpiperidin-1-yl)-1,4-dihydroquinoline-3-carboxylic acid (24)

General procedure D: starting from 63 (reaction time: 1 h; work-up: extraction at pH = 3), compound 24, after crystallization by EtOH/DMF, was obtained as a yellow solid in 33% yield (0.16 g). ^1^H NMR (400 MHz, DMSO-*d*_6_): *δ* 1.08–1.15 (m, 2H, cyclopropyl-CH_2_), 1.22–1.29 (m, 2H, cyclopropyl-CH_2_), 1.83–1.89 (m, 2H, piperidine-CH_2_), 1.92–2.03 (m, 2H, piperidine-CH_2_), 2.64–2.82 (m, 3H, piperidine-NCH_2_ and -CH), 3.43–3.51 (m, 2H, piperidine-NCH_2_), 3.74–3.83 (m, 1H, CH), 5.44 (s, 2H, NH_2_), 7.15–7.20 (m, 1H, H-4′), 7.30 (d, *J* = 4.3 Hz, 4H, H-2′, H-3′, H-5′ and H-6′), 7.50 (s, 1H, H-5), 7.58 (s, 1H, H-8), 8.48 (s, 1H, H-2), 15.89 (s, 1H, CO_2_H). ^13^C NMR (100 MHz, DMSO-*d*_6_): *δ* 7.79, 33.42, 35.99, 42.01, 51.07, 106.15, 106.52, 107.45, 121.85, 126.54, 127.20, 128.78, 134.28, 142.59, 144.90, 146.51, 146.76, 167.21, 176.65. HPLC: r_t_ 5.672 min. HRMS (ESI) calculated for C_24_H_25_N_3_O_3_ [M + H]^+^ 404.1974, found 404.1971.

#### 6-Amino-7-[benzyl(ethyl)amino]-1-cyclopropyl-4-oxo-1,4-dihydroquinoline-3-carboxylic acid (25)

General procedure D: starting from 64 (reaction time: 2 h; work-up: extraction at pH = 3), compound 25, after crystallization by EtOH/DMF, was obtained as a pale yellow solid in 70% yield (0.12 g). ^1^H NMR (400 MHz, DMSO-*d*_6_): *δ* 0.87–0.93 (m, 2H, cyclopropyl-CH_2_), 1.05 (t, *J* = 7.0 Hz, 3H, NCH_2_*CH*_3_), 1.16–1.25 (m, 2H, cyclopropyl-CH_2_), 3.12 (q, *J* = 6.9 Hz, 2H, N*CH*_2_CH_3_), 3.58–3.73 (m, 1H, CH), 4.33 (s, 2H, NCH_2_), 5.51 (s, 2H, NH_2_), 7.14 (t, *J* = 7.2 Hz, 1H, H-4′), 7.22 (t, *J* = 7.4 Hz, 2H, H-3′ and H-5′), 7.30 (d, *J* = 7.1 Hz, 2H, H-2′ and H-6′), 7.45–7.49 (m, 2H, H-5 and H-8), 8.42 (s, 1H, H-2), 15.83 (s, 1H, CO_2_H). ^13^C NMR (100 MHz, DMSO-*d*_6_): *δ* 7.82, 11.93, 35.94, 45.74, 54.12, 105.82, 106.68, 110.57, 121.98, 127.43, 128.63, 128.69, 133.74, 138.27, 143.47, 143.68, 145.18, 167.33, 176.68. HPLC: r_t_ 5.114 min. HRMS (ESI) calculated for C_22_H_23_N_3_O_3_ [M + H]^+^ 378.1817, found 378.18165.

#### 6-Amino-7-[benzyl(2-hydroxyethyl)amino]-1-cyclopropyl-4-oxo-1,4-dihydroquinoline-3-carboxylic acid (26)

General procedure D: starting from 65 (reaction time: 2 h; work-up: extraction at pH = 3), compound 26, after crystallization by EtOH/DMF, was obtained as a yellow solid in 58% yield (0.14 g). ^1^H NMR (400 MHz, DMSO-*d*_6_): *δ* 0.81–0.88 (s, 2H, cyclopropyl-CH_2_), 1.16 (q, *J* = 6.5 Hz, 2H, cyclopropyl-CH_2_), 3.15 (t, *J* = 5.1 Hz, 2H, *CH*_2_CH_2_OH), 3.53–3.62 (m, 1H, CH), 3.63–3.69 (m, 2H, CH_2_*CH*_2_OH), 4.45 (s, 2H, CH_2_), 5.07 (t, *J* = 4.6 Hz, 1H, OH), 5.91 (s, 2H, NH_2_), 7.11 (t, *J* = 7.2 Hz, 1H, H-4′), 7.20 (t, *J* = 7.5 Hz, 2H, H-3′ and H-5′), 7.31 (d, *J* = 7.4 Hz, 2H, H-2′ and H-6′), 7.39 (s, 1H, H-5), 7.48 (s, 1H, H-8), 8.38 (s, 1H, H-2), 15.88 (s, 1H, CO_2_H). ^13^C NMR (100 MHz, DMSO-*d*_6_): *δ* 7.75, 35.85, 53.75, 54.65, 58.54, 105.91, 106.17, 109.63, 121.61, 127.22, 128.48 (2C), 133.41, 138.51, 143.31, 143.73, 144.68, 167.20, 176.45. HPLC: r_t_ 6.778 min. HRMS (ESI) calculated for C_22_H_23_N_3_O_4_ [M + H]^+^ 394.1767, found 394.17666.

#### 7-Chloro-1-cyclopropyl-6-nitro-4-oxo-1,4-dihydroquinoline-3-carboxylic acid (66)

General procedure A: starting from 45 (ref. 31) (reaction time: 3 h), compound 66 was obtained as a yellow solid in 95% yield (1.30 g). ^1^H NMR (400 MHz, DMSO-*d*_6_): *δ* 1.11–1.19 (m, 2H, cyclopropyl-CH_2_), 1.25–1.29 (m, 2H, cyclopropyl-CH_2_), 3.81–3.69 (m, 1H, CH), 8.51 (s, 1H, H-8), 8.76 (s, 1H, H-5), 8.87 (s, 1H, H-2), 13.94 (bs, 1H, CO_2_H).

#### 7-Chloro-1-cyclopropyl-6-nitro-4-oxo-1,4-dihydroquinoline-3-carboxamide (67)

SOCl_2_ (5 mL) was added to acidic derivative 66 (0.50 g, 1.62 mmol) and the reaction mixture was stirred at reflux for 2 h. Then, the excess of SOCl_2_ was removed under vacuum and the resulting yellow oil was dissolved in dry DMF (10 mL) and added dropwise at 0 °C to a solution of 7 N NH_3_ in MeOH. The reaction mixture was allowed to warm at rt and stirred for 3 h and then it was poured into ice/water to give a precipitate that was collected by filtration. After trituration with a mixture of Et_2_O/EtOH, compound 67 was obtained as a yellow solid in 92% yield. ^1^H NMR (400 MHz, DMSO-*d*_6_): *δ* 1.10–1.13 (m, 2H, cyclopropyl-CH_2_), 1.24–1.29 (m, 2H, cyclopropyl-CH_2_), 3.73–3.76 (m, 1H, CH), 7.67–7.68 (m, 1H, NH_2_ ×½), 8.37 (s, 1H, H-8), 8.68 (s, 1H, H-5), 8.82 (s, 1H, H-2), 78.81–8.83 (m, 1H, NH_2_ ×½).

#### 1-Cyclopropyl-7-(3,4-dihydroisoquinolin-2(1*H*)-yl)-6-nitro-4-oxo-1,4-dihydroquinoline-3-carboxamide (68)

General procedure B: starting from 67 and using 1,2,3,4-tetrahydroisoquinoline (3 equiv.) as a nucleophile (reaction time: 3 h; temperature: 90 °C; solvent: DMSO), compound 68 was obtained as a yellow solid in 96% yield (0.19 g). ^1^H NMR (400 MHz, DMSO-*d*_6_): *δ* 1.03–1.09 (m, 2H, cyclopropyl-CH_2_), 1.26–1.28 (m, 2H, cyclopropyl-CH_2_), 2.87–2.96 (m, 2H, tetrahydroisoquinoline-CH_2_), 3.43 (t, *J* = 7.3 Hz, 2H, tetrahydroisoquinoline-CH_2_), 3.70–3.74 (m, 1H, CH), 1.91–1.97 (m, 2H, tetrahydroisoquinoline-CH_2_), 7.17–7.21 (m, 3H, Ar–H), 7.22–7.25 (m, 1H, Ar–H), 7.53–7.54 (m, 1H, NH_2_ ×½), 7.59 (s, 1H, H-8), 8.58–8.61 (m, 2H, H-2 and H-5), 8.96–8.97 (m, 1H, NH_2_ ×½).

#### 6-Amino-1-cyclopropyl-7-(3,4-dihydroisoquinolin-2(1*H*)-yl)-4-oxo-1,4-dihydroquinoline-3-carboxamide (17)

General procedure C: starting from 68 (reaction time: 5 h), compound 17, after purification by chromatography column (Reveleris-X2) eluting with CHCl_3_/MeOH 97 : 3 to 95 : 5, was obtained as a yellow solid in 25% yield (0.07 g). ^1^H NMR (400 MHz, DMSO-*d*_6_): *δ* 0.91–1.01 (m, 2H, cyclopropyl-CH_2_), 1.12–1.16 (m, 2H, cyclopropyl-CH_2_), 2.95 (t, *J* = 5.5 Hz, 2H, tetrahydroisoquinoline-CH_2_), 3.33 (t, *J* = 5.7 Hz, 2H, tetrahydroisoquinoline-CH_2_), 3.52–3.62 (m, 1H, CH), 4.24 (s, 2H, tetrahydroisoquinoline-CH_2_), 5.20 (s, 2H, NH_2_), 7.13–7.17 (m, 4H, Ar–H), 7.30 (d, *J* = 4.8 Hz, 1H, CONH_2_ ×½), 7.51–7.54 (m, 2H, H-5 and H-8), 8.46 (s, 1H, H-2), 9.43 (d, *J* = 4.9 Hz, 1H, CONH_2_ ×½). ^13^C NMR (100 MHz, DMSO-*d*_6_): *δ* 7.67, 28.63, 34.97, 47.97, 52.66, 107.33, 108.23, 109.72, 124.07, 126.14, 126.70, 126.77, 129.12, 133.68, 134.52, 134.83, 141.36, 144.39, 144.81, 166.56, 174.96. HPLC: r_t_ 4.557 min. HRMS (ESI) calculated for C_22_H_22_N_4_O_2_ [M + Na]^+^ 397.16407, found 397.16376.

#### Ethyl 7-(3,4-dihydroisoquinolin-2(1*H*)-yl)-1-ethyl-6-nitro-4-oxo-1,4-dihydroquinoline-3-carboxylate (69)

General procedure B: starting from 46 (ref. 37) and using 1,2,3,4-tetrahydroisoquinoline (3 equiv.) as nucleophile (reaction time: 2 h; temperature: 90 °C; solvent: DMSO), compound 69 was obtained as a yellow solid in 88% yield (0.50 g). ^1^H NMR (200 MHz, DMSO-*d*_6_): *δ* 1.18–1.45 (m, 6H, OCH_2_*CH*_3_ and NCH_2_*CH*_3_), 2.89*–*3.02 (m, 2H, tetrahydroisoquinoline-CH_2_), 3.43 (t, *J* = 5.6 Hz, 2H, tetrahydroisoquinoline-CH_2_), 4.26 (q, *J* = 6.9 Hz, 2H, O*CH*_2_CH_3_), 4.27–4.53 (m, 4H, NCH_2_*CH*_3_ and tetrahydroisoquinoline-CH_2_), 7.00–7.25 (m, 5H, Ar-H), 8.50 (s, 1H, H-2), 8.70 (s, 1H, H-5).

#### Ethyl 6-amino-7-(3,4-dihydroisoquinolin-2(1*H*)-yl)-1-ethyl-4-oxo-1,4-dihydroquinoline-3-carboxylate (70)

General procedure C: starting from 69 (reaction time: 2 h), compound 70, after trituration by EtOH, was obtained as a white solid in 70% yield (0.47 g). ^1^H NMR (400 MHz, DMSO-*d*_6_): *δ* 1.00–1.30 (m, 6H, OCH_2_*CH*_3_ and NCH_2_*CH*_3_), 2.92–3.01 (m, 2H, tetrahydroisoquinoline-CH_2_), 3.25–3.32 (m, 2H, tetrahydroisoquinoline-CH_2_), 4.20–4.30 (m, 6H, N*CH*_2_CH_3_, O*CH*_2_CH_3_ and tetrahydroisoquinoline-CH_2_), 5.18 (s, 2H, NH_2_), 7.05–7.25 (m, 4H, Ar–H), 7.45 (s, 1H, H-8), 7.48 (s, 1H, H-5), 8.27 (s, 1H, H-2).

#### 6-Amino-7-(3,4-dihydroisoquinolin-2(1*H*)-yl)-1-ethyl-4-oxo-1,4-dihydroquinoline-3-carboxylic acid (16)

General procedure D: starting from 70 (reaction time: 4 h; work-up: extraction at pH = 5), compound 16, after crystallization by EtOH/DMF, was obtained as a pale yellow solid in 76% yield (0.30 g). ^1^H NMR (400 MHz, DMSO-*d*_6_): *δ* 1.36 (t, *J* = 7.0 Hz, 3H, NCH_2_*CH*_3_), 3.02 (t, *J* = 5.4 Hz, 2H, tetrahydroisoquinoline-CH_2_), 3.36 (t, *J* = 5.4 Hz, 2H, tetrahydroisoquinoline-CH_2_), 4.31 (s, 2H, tetrahydroisoquinoline-CH_2_), 4.52 (q, *J* = 7.0 Hz, 2H, N*CH*_2_CH_3_), 5.47 (s, 2H, NH_2_), 7.10–7.20 (m, 4H, Ar–H), 7.30 (s, 1H, H-8), 7.59 (s, 1H, H-5), 8.77 (s, 1H, H-2), 16.04 (s, 1H, CO_2_H). ^13^C NMR (100 MHz, DMSO-*d*_6_): *δ* 14.98, 28.89, 48.13, 49.25, 52.53, 106.52, 107.16, 107.41, 122.69, 126.18, 126.75, 126.79, 129.16, 132.19, 134.45, 134.75, 142.52, 145.51, 146.03, 167.41, 176.48. HPLC: r_t_ 5.2700 min. HRMS (ESI) calculated for C_21_H_21_N_3_O_3_ [M + H]^+^ 364.1661, found 364.1659.

#### Ethyl 7-chloro-1-ethyl-6-fluoro-4-oxo-1,4-dihydro-1,8-naphthyridine-3-carboxylate (71)

To a solution of the commercially available ethyl 3-(2,6-dichloro-5-fluoropyridin-3-yl)-3-oxopropanoate (0.5 g, 2.0 mmol) in toluene (3 mL), DMF–DMA (0.3 mL, 2.2 mmol) and acetic acid (30 μL, 0.5 mmol) were added and the reaction mixture was stirred at rt for 2 h. After the disappearance of the starting material as checked by TLC (cyclohexane/EtOAc 60 : 40), 2 M EtNH_2_ in THF (1.1 mL, 2.2 mmol) was added and the reaction mixture was stirred for additional 30 min at rt. Then, 3 mL of aq. 10% citric acid were added, and the two phases were separated; the organic phase was washed with water and aq. 1.5 M BuN_4_OH (1.5 mL, 2.2 mmol) was added. The reaction mixture was stirred for 5 min until the formation of a precipitate, then it was concentrated under vacuum to half volume and neutralized by 2 N HCl and the precipitate was filtered under vacuum to afford compound 71 as a white solid in 63% yield. ^1^H NMR (200 MHz, DMSO-*d*_6_): *δ* 1.19–1.38 (m, 6H, OCH_2_*CH*_3_, and NCH_2_*CH*_3_), 4.22 (q, *J* = 7.1 Hz, 2H, O*CH*_2_CH_3_), 4.40 (q, *J* = 7.1 Hz, 2H, N*CH*_2_CH_3_), 8.43 (d, *J* = 7.9 Hz, 1H, H-5), 8.85 (s, 1H, H-2).

#### Ethyl 7-chloro-1-cyclopropyl-6-fluoro-4-oxo-1,4-dihydro-1,8-naphthyridine-3-carboxylate (72)

Following the same procedure used to prepare compound 71 and replacing EtNH_2_ with cyclopropyl amine, ethyl ester intermediate 72, after purification by flash chromatography column eluting with CH_2_Cl_2_/MeOH 95 : 5, was obtained in 53% yield (0.72 g). ^1^H NMR (200 MHz, DMSO-*d*_6_): *δ* 0.93–1.06 (m, 2H, cyclopropyl-CH_2_), 1.09–1.16 (m, 2H, cyclopropyl-CH_2_), 1.25 (t, *J* = 7.1 Hz, 3H, OCH_2_*CH*_3_), 3.61–3.64 (m, 1H, CH), 4.20 (q, *J* = 7.1 Hz, 2H, O*CH*_2_CH_3_), 8.39 (d, *J* = 7.8 Hz, 1H, H-5), 8.53 (s, 1H, H-2).

#### Ethyl 7-(3,4-dihydroisoquinolin-2(1*H*)-yl)-1-ethyl-6-fluoro-4-oxo-1,4-dihydro-1,8-naphthyridine-3-carboxylate (73)

General procedure B: starting from 71 and using 1,2,3,4-tetrahydroisoquinoline (3 equiv.) as a nucleophile (reaction time: 2 h; temperature: 90 °C; solvent: DMF), compound 73 was obtained as a white solid in 83% yield (0.23 g). ^1^H NMR (200 MHz, DMSO-*d*_6_): *δ* 1.21–1.36 (m, 6H, OCH_2_*CH*_3_, and NCH_2_*CH*_3_), 2.96 (t, *J* = 5.8 Hz, 2H, tetrahydroisoquinoline-CH_2_), 3.96 (t, *J* = 5.8 Hz, 2H, tetrahydroisoquinoline-CH_2_), 4.17 (q, *J* = 7.0 Hz, 2H, O*CH*_2_CH_3_), 4.40 (q, *J* = 7.1 Hz, 2H, N*CH*_2_CH_3_), 4.87 (s, 2H, tetrahydroisoquinoline-CH_2_), 7.10–7.30 (m, 4H, Ar–H), 7.91 (d, *J* = 13.6 Hz, 1H, H-5), 8.61 (s, 1H, H-2).

#### Ethyl 1-cyclopropyl-7-(3,4-dihydroisoquinolin-2(1*H*)-yl)-6-fluoro-4-oxo-1,4-dihydro-1,8-naphthyridine-3-carboxylate (74)

General procedure B: starting from 72 and using 1,2,3,4-tetrahydroisoquinoline (3 equiv.) as a nucleophile (reaction time: 3 h; temperature: 90 °C; solvent: DMF), compound 74 was obtained as a white solid in 94% yield (0.62 g). ^1^H NMR (400 MHz, DMSO-*d*_6_): *δ* 0.98–1.06 (m, 2H, cyclopropyl-CH_2_), 1.14–1.16 (m, 2H, cyclopropyl-CH_2_), 1.22 (t, *J* = 7.0 Hz, 3H, OCH_2_*CH*_3_), 2.95–2.99 (m, 2H, tetrahydroisoquinoline-CH_2_), 3.51–3.58 (m, 1H, CH), 3.99 (t, *J* = 7.2 Hz, 2H, tetrahydroisoquinoline-CH_2_), 4.15 (q, *J* = 7.0 Hz, 2H, O*CH*_2_CH_3_), 4.24 (s, 2H, tetrahydroisoquinoline-CH_2_), 7.10–7.19 (m, 3H, Ar–H), 7.21–7.23 (m, 1H, Ar–H), 7.86 (d, *J* = 13.6 Hz, 1H, H-5), 8.34 (s, 1H, H-2).

#### Ethyl 7-[benzyl(ethyl)amino]-1-ethyl-6-fluoro-4-oxo-1,4-dihydro-1,8-naphthyridine-3-carboxylate (75)

General procedure B: starting from 71 and using *N*-benzylethanamine (3 equiv.) as a nucleophile (reaction time: 3 h; temperature: 90 °C; solvent: DMF), compound 75 was obtained as a white solid in 74% yield (0.40 g). ^1^H NMR (400 MHz, DMSO-*d*_6_): *δ* 1.13 (t, *J* = 7.0 Hz, 3H, NCH_2_*CH*_3_), 1.24–1.29 (m, 6H, NCH_2_*CH*_3_ and OCH_2_*CH*_3_), 3.69 (t, *J* = 6.3 Hz, 2H, N*CH*_2_CH_3_), 4.20 (t, *J* = 7.0 Hz, N*CH*_2_CH_3_ and O*CH*_2_CH_3_), 4.90 (s, 2H, CH_2_), 7.24–7.36 (m, 5H, Ar–H), 7.90 (d, *J* = 14.0 Hz, 1H, H-5), 8.58 (s, 1H, H-2).

#### Ethyl 1-ethyl-6-fluoro-4-oxo-7-(4-phenylpiperidin-1-yl)-1,4-dihydro-1,8-naphthyridine-3-carboxylate (76)

General procedure B: starting from 71 and using 4-phenylpiperidine (3 equiv.) as a nucleophile (reaction time: 3 h; temperature: 90 °C; solvent: DMF), compound 76 was obtained as a whitish solid in 78% yield (0.45 g). ^1^H NMR (400 MHz, DMSO-*d*_6_): *δ* 1.28 (t, *J* = 6.3 Hz, 2H, OCH_2_*CH*_3_), 1.33–1.39 (m, 2H, NCH_2_*CH*_3_), 1.75–1.77 (m, 2H, piperidine-CH_2_), 1.89–1.91 (m, 2H, piperidine-CH_2_), 2.88–2.919 (m, 1H, piperidine-CH), 3.20 (t, *J* = 2.7 Hz, 2H, piperidine-NCH_2_), 4.20–4.23 (m, 2H, O*CH*_2_CH_3_), 4.35–4.39 (m, 2H, N*CH*_2_CH_3_), 4.51–4.55 (m, 2H, piperidine-NCH_2_), 7.20–7.23 (m, 1H, Ar-H), 7.28–7.35 (m, 4H, Ar-H), 7.93 (d, *J* = 13.9 Hz, 1H, H-5), 8.66 (s, 1H, H-2).

#### 7-(3,4-Dihydroisoquinolin-2(1*H*)-yl)-1-ethyl-6-fluoro-4-oxo-1,4-dihydro-1,8-naphthyridine-3-carboxylic acid (27)

General procedure A: starting from 73 (reaction time: 4 h), compound 27, after crystallization by DMF, was obtained as a pale yellow solid in 40% yield (0.07 g). ^1^H NMR (400 MHz, DMSO-*d*_6_): *δ* 1.40 (t, *J* = 6.9 Hz, 3H, NCH_2_*CH*_3_), 3.00 (t, *J* = 5.9 Hz, 2H, tetrahydroisoquinoline-CH_2_), 4.05 (t, *J* = 5.9 Hz, 2H, tetrahydroisoquinoline-CH_2_), 4.53 (q, *J* = 6.9 Hz, 2H, N*CH*_2_CH_3_), 4.97 (s, 2H, tetrahydroisoquinoline-CH_2_), 7.10–7.30 (m, 4H, Ar-H), 8.07 (d, *J* = 13.4 Hz, 1H, H-5), 8.97 (s, 1H, H-2), 15.37 (s, 1H, CO_2_H). ^13^C NMR (100 MHz, DMSO-*d*_6_): *δ* 15.17, 28.50, 45.49 (d, *J*_C–F_ = 8.0 Hz), 47.69, 48.78 (d, *J*_C–F_ = 7.0 Hz), 108.46, 112.74 (d, *J*_C–F_ = 3.0 Hz), 119.56 (d, *J*_C–F_ = 22.0 Hz), 126.71, 126.90, 127.21, 128.80, 133.96, 135.08, 145.39, 147.22 (d, *J*_C–F_ = 257.0 Hz), 148.09, 150.47 (d, *J*_C–F_ = 10.0 Hz), 166.36, 176.76. HPLC r_t_: 6.0900 min. HRMS-ESI *m*/*z* [M + H]^+^ calcd for C_20_H_18_FN_3_O_3_, 368.1410, found: 368.1405.

#### 1-Cyclopropyl-7-(3,4-dihydroisoquinolin-2(1*H*)-yl)-6-fluoro-4-oxo-1,4-dihydro-1,8-naphthyridine-3-carboxylic acid (28)

General procedure A: starting from 74 (reaction time: 6 h), compound 28, after crystallization by EtOH/DMF, was obtained as a white solid in 67% yield (0.25 g). ^1^H NMR (400 MHz, DMSO-*d*_6_): *δ* 1.01–1.10 (m, 2H, cyclopropyl-CH_2_), 1.18–1.22 (m, 2H, cyclopropyl-CH_2_), 2.99 (t, *J* = 5.8 Hz, 2H, tetrahydroisoquinoline-CH_2_), 3.62–3.76 (m, 1H, CH), 4.05 (t, *J* = 5.9 Hz, 2H, tetrahydroisoquinoline-CH_2_), 4.98 (s, 2H, tetrahydroisoquinoline-CH_2_), 7.15–7.21 (m, 3H, Ar–H), 7.21–7.27 (m, 1H, Ar–H), 8.00 (d, *J* = 13.5 Hz, 1H, H-5), 8.55 (s, 1H, H-2), 15.18 (s, 1H, CO_2_H). ^13^C NMR (100 MHz, DMSO-*d*_6_): *δ* 7.24, 28.41, 35.39, 45.35 (d, *J*_C–F_ = 9.1 Hz), 48.72 (d, *J*_C–F_ = 4.0 Hz), 107.95, 112.31, 119.44 (d, *J*_C–F_ = 21.2 Hz), 126.62, 126.85, 127.13, 128.80, 133.98, 133.00, 147.18, 147.19 (d, *J*_C–F_ = 260.6 Hz), 147.48, 150.13 (d, *J*_C–F_ = 9.1 Hz), 166.15, 176.80. HPLC: r_t_ 10.251 min. HRMS (ESI) calculated for C_21_H_18_FN_3_O_3_ [M + H]^+^ 380.1410, found 380.14075.

#### 7-[Benzyl(ethyl)amino]-1-ethyl-6-fluoro-4-oxo-1,4-dihydro-1,8-naphthyridine-3-carboxylic acid (29)

General procedure D: starting from 75 (reaction time: 1 h; work-up: filtration at pH = 3), compound 29, after crystallization by EtOH, was obtained as a yellow solid in 77% yield (0.21 g). ^1^H NMR (400 MHz, DMSO-*d*_6_): *δ* 1.16–1.18 (m, 3H, NCH_2_*CH*_3_), 1.28 (t, *J* = 7.7 Hz, 3H, NCH_2_*CH*_3_), 3.74–3.77 (m, 2H, N*CH*_2_CH_3_), 1.33–1.38 (m, 2H, N*CH*_2_CH_3_), 4.96 (s, 2H, CH_2_), 7.25–7.37 (m, 5H, Ar–H), 8.05 (d, *J* = 13.7 Hz, 1H, H-5), 8.91 (s, 1H, H-2). ^13^C NMR (100 MHz, DMSO-*d*_6_): *δ* 14.07, 15.17, 46.22 (d, *J*_C–F_ = 8.0 Hz), 47.46, 53.32, 108.49, 112.34, 119.61 (d, *J*_C–F_ = 22.0 Hz), 126.95, 127.53, 129.13, 138.40, 145.51 (d, *J*_C–F_ = 22.0 Hz), 146.83 (d, *J*_C–F_ = 239.0 Hz), 148.02, 160.15 (d, *J*_C–F_ = 8.0 Hz), 166.47, 176.77. HPLC: r_t_ 6.778 min. HRMS (ESI) calculated for C_20_H_20_FN_3_O_3_ [M + H]^+^ 370.1575, found 370.15737.

#### 1-Ethyl-6-fluoro-4-oxo-7-(4-phenylpiperidin-1-yl)-1,4-dihydro-1,8-naphthyridine-3-carboxylic acid (30)

General procedure A: starting from 76 (reaction time: 16 h), compound 30, after crystallization by EtOH/DMF, was obtained as a white solid in 76% yield (0.29 g). ^1^H NMR (400 MHz, DMSO-*d*_6_): *δ* 1.41 (t, *J* = 7.0 Hz, 3H, NCH_2_*CH*_3_), 1.75–1.82 (m, 2H, piperidine-CH_2_), 1.92–1.96 (m, 2H, piperidine-CH_2_), 2.94 (t, *J* = 12.0 Hz, 1H, piperidine-CH), 3.28 (t, *J* = 12.7 Hz, 2H, piperidine-NCH_2_), 4.50 (q, *J* = 6.7 Hz, 2H, N*CH*_2_CH_3_), 4.64 (d, *J* = 13.1 Hz, 2H, piperidine-NCH_2_), 7.20 (t, *J* = 6.6 Hz, 1H, Ar–H), 7.25–7.32 (m, 4H, Ar–H), 8.05 (d, *J* = 13.7 Hz, 1H, H-5), 8.97 (s, 1H, H-2). ^13^C NMR (100 MHz, DMSO-*d*_6_): *δ* 15.28, 33.42, 42.26, 47.77, 48.13 (d, *J*_C–F_ = 8.1 Hz), 108.55, 112.96, 119.89 (d, *J*_C–F_ = 22.2 Hz), 126.81, 127.33, 128.99, 145.61, 146.28, 147.10 (d, *J*_C–F_ = 226.2 Hz), 150.48, 160.56, 166.42 (d, *J*_C–F_ = 14.0 Hz), 176.88. HPLC: r_t_ 7.332 min. HRMS (ESI) calculated for C_22_H_22_FN_3_O_3_ [M + H]^+^ 396.1723, found 396.17333.

#### Ethyl (2*E/Z*)-3-(dimethylamino)-2-(2,4,5-trifluoro-3-methylbenzoyl)acrylate (77)

Under N_2_ atmosphere, to a mixture of 2,4,5-trifluoro-3-methylbenzoic acid (6.80 g, 35.8 mmol) in dry CH_2_Cl_2_ (120 mL), oxalyl chloride (3.7 mL, 42.9 mmol) and cat. dry DMF were added, and the reaction mixture was stirred at rt for 4 h. The solvent was removed under vacuum and the oil residue was dissolved in dry toluene (100 mL); the resulting solution was added dropwise, under N_2_ atmosphere, to a mixture of ethyl *N*,*N*-dimethylaminoacrylate (6.7 mL, 46.5 mmol) and Et_3_N (7.46 mL, 53.7 mmol) in dry toluene (50 mL) and the reaction mixture was heated at 90 °C for 4 h. After cooling, the reaction mixture was filtered, and the filtrate was evaporated to give compound 77 as a pale brown solid in 70% yield. ^1^H NMR (200 MHz, CDCl_3_): *δ* 0.98 (t, *J* = 7.1 Hz. 3H, OCH_2_*CH*_3_), 2.21 (t, *J* = 1.8 Hz, 3H, CH_3_), 2.60–3.40 (m, 6H, N(CH_3_)_2_), 4.00 (q, *J* = 7.1 Hz, 2H, O*CH*_2_CH_3_), 7.20–7.35 (m, 1H, Ar–H), 7.78 (s, 1H, CH).

#### Ethyl (2*E/Z*)-3-(ethylamino)-2-(2,4,5-trifluoro-3-methylbenzoyl)acrylate (78)

Following the same procedure used to prepare compound 35 and starting from 77, intermediate 78 was obtained in 90% yield (0.51 g). ^1^H NMR (200 MHz, CDCl_3_): *δ* 1.05 (t, *J* = 7.1 Hz, 3H, OCH_2_*CH*_3_), 1.26 (t, *J* = 7.2 Hz, 3H, NCH_2_*CH*_3_), 2.25 (t, *J* = 1.8 Hz, 3H, CH_3_), 3.50 (q, *J* = 7.2 Hz, 2H, N*CH*_2_CH_3_), 4.00 (q, *J* = 7.1 Hz. 3H, O*CH*_2_CH_3_), 7.20–7.35 (m, 1H, Ar–H), 7.78 (d, *J* = 14.1 Hz, 1H, CH), 10.80 (bs, 1H, NH).

#### Ethyl (2*E/Z*)-3-(cyclopropylamino)-2-(2,4,5-trifluoro-3-methylbenzoyl)acrylate (79)

Following the same procedure used to prepare compound 35, replacing ethylamine with cyclopropyl amine and starting from 77, intermediate 79 was obtained in 99% yield (1.85 g). ^1^H NMR (200 MHz, CDCl_3_): *δ* 0.81–0.99 (m, 4H, cycloprpyl-CH_2_ ×2), 1.06 (t, *J* = 7.1 Hz, 3H, OCH_2_*CH*_3_), 2.18–2.23 (m, 3H, CH_3_), 2.93–3.03 (m, 1H, CH), 4.06 (t, *J* = 7.1 Hz, 2H, O*CH*_2_CH_3_), 6.95–7.10 (m, 1H, Ar–H), 8.14–8.17 (m, 1H, CH), 10.81–10.87 (m, 1H, NH).

#### Ethyl 1-ethyl-6,7-difluoro-8-methyl-4-oxo-1,4-dihydroquinoline-3-carboxylate (80)

Under N_2_ atmosphere, to a mixture of 60% NaH (1.71 g, 43.0 mmol) in dry DMF (20 mL) cooled at 0 °C, a solution of acrylate intermediate 78 (4.5 g, 14.0 mmol) in dry DMF (50 mL) was added dropwise. After stirring at 0 °C for 3 h, the reaction mixture was poured into ice/water, acidified with 2 N HCl (pH = 4–5) and the precipitate was filtered under vacuum to afford compound 80 as a white solid in 90% yield. ^1^H NMR (200 MHz, DMSO-*d*_6_): *δ* 1.22–1.30 (m, 6H, OCH_2_*CH*_3_ and NCH_2_*CH*_3_), 2.59 (d, *J* = 3.0 Hz, 3H, CH_3_), 4.21 (q, *J* = 7.1 Hz, 2H, O*CH*_2_CH_3_), 4.49 (q, *J* = 7.3 Hz, 2H, N*CH*_2_CH_3_), 7.92–8.02 (m, 1H, H-5), 8.62 (s, 1H, H-2).

#### Ethyl 1-cyclopropyl-6,7-difluoro-8-methyl-4-oxo-1,4-dihydroquinoline-3-carboxylate (81)

Following the same procedure used to prepare compound 80 and starting from 79, intermediate 81 was obtained as a white solid in 97% yield (1.65 g). ^1^H NMR (200 MHz, DMSO-*d*_6_): *δ* 0.93–0.97 (m, 2H, cyclopropyl-CH_2_), 1.07–1.15 (m, 5H, cyclopropyl-CH_2_ and OCH_2_*CH*_3_), 2.77 (d, *J* = 3.4 Hz, 3H, CH_3_), 3.31–3.36 (m, 1H, CH), 4.21 (q, *J* = 7.0 Hz, 2H, O*CH*_2_CH_3_), 7.87–7.95 (m, 1H, H-5), 8.59 (s, 1H, H-2).

#### Ethyl 7-azido-1-ethyl-6-fluoro-8-methyl-4-oxo-1,4-dihydroquinoline-3-carboxylate (82)

To solution of intermediate 80 (3.00 g, 10.1 mmol) in DMF, NaN_3_ (2.60 g, 40.4 mmol) was added, and the reaction mixture was heated at 90 °C for 24 h. After cooling, the mixture was poured into ice/water, acidified with 2 N HCl (pH = 5) and the precipitate was filtered under vacuum to afford compound 82 as a brown solid in 73% yield. ^1^H NMR (200 MHz, DMSO-*d*_6_): *δ* 1.00–1.35 (m, 6H, OCH_2_*CH*_3_ and NCH_2_*CH*_3_), 2.55 (s, 3H, CH_3_), 4.15 (q, *J* = 7.1 Hz, 2H, O*CH*_2_CH_3_), 4.40 (q, *J* = 7.3 Hz, 2H, N*CH*_2_CH_3_), 7.86 (d, *J* = 11.4 Hz, 1H, H-5), 8.63 (s, 1H, H-2).

#### Ethyl 7-azido-1-cyclopropyl-6-fluoro-8-methyl-4-oxo-1,4-dihydroquinoline-3-carboxylate (83)

Following the same procedure used to prepare compound 82 and starting from 81, intermediate 83 was obtained as a light brown solid in 74% yield (2.85 g). ^1^H NMR (200 MHz, DMSO-*d*_6_): *δ* 0.85–0.89 (m, 2H, cyclopropyl-CH_2_), 1.08–1.14 (m, 2H, cyclopropyl-CH_2_), 1.26 (t, *J* = 7.1 Hz, 3H, OCH_2_*CH*_3_), 2.69 (s, 3H, CH_3_), 4.12–4.23 (m, 3H, CH and O*CH*_2_CH_3_), 7.79 (d, *J* = 11.8 Hz, 1H, H-5), 8.58 (s, 1H, H-2).

#### Ethyl 7-amino-1-ethyl-6-fluoro-8-methyl-4-oxo-1,4-dihydroquinoline-3-carboxylate (84)

A solution of azido intermediate 82 (2.40 g, 7.5 mmol) in a mixture MeOH/THF (1 : 1) (300 mL) was hydrogenated over a catalytic amount of Pd/C (10% w/w) at rt for 3 h. Then, the reaction mixture was filtered over celite and the filtrate was evaporated to give, after purification by flash column chromatography eluting CHCl_3_/MeOH 95 : 5, compound 84 as a white solid in 53% yield. ^1^H NMR (200 MHz, DMSO-*d*_6_): *δ* 1.05–1.30 (m, 6H, OCH_2_*CH*_3_ and NCH_2_*CH*_3_), 2.25 (s, 3H, CH_3_), 4.10–4.40 (m, 4H, O*CH*_2_CH_3_, and N*CH*_2_CH_3_), 6.00 (s, 2H, NH_2_), 7.61 (d, *J* = 11.6 Hz, 1H, H-5), 8.50 (s, 1H, H-2).

#### Ethyl 7-amino-1-cyclopropyl-6-fluoro-8-methyl-4-oxo-1,4-dihydroquinoline-3-carboxylate (85)

Following the same procedure used to prepare compound 84 and starting from 83, derivative 85 was obtained as a yellow solid in 70% yield (1.15 g). ^1^H NMR (200 MHz, DMSO-*d*_6_): *δ* 0.72–0.80 (m, 2H, cyclopropyl-CH_2_), 1.08–1.14 (m, 2H, cyclopropyl-CH_2_), 1.23 (t, *J* = 6.9 Hz, 3H, OCH_2_*CH*_3_), 2.49 (s, 3H, CH_3_), 4.15–4.21 (m, 3H, CH and O*CH*_2_CH_3_), 5.96 (bs, 2H, NH_2_), 7.56 (d, *J* = 11.3 Hz, 1H, H-5), 8.50 (s, 1H, H-2).

#### Ethyl 7-bromo-1-ethyl-6-fluoro-8-methyl-4-oxo-1,4-dihydroquinoline-3-carboxylate (86)

To a mixture of amino intermediate 84 (1.30 g, 4.4 mmol) and CuBr_2_ (4.90 g, 22.0 mmol) in aq. 5% HBr (50 mL) cooled at 0 °C, aq. 2 M NaNO_2_ (4.40 mL, 8.8 mmol) was added dropwise, and the reaction mixture was stirred at rt for 30 min. Then, the mixture was poured into ice/water and the precipitate was filtered to give compound 86 as a pale brown solid in 82% yield. ^1^H NMR (200 MHz, DMSO-*d*_6_): *δ* 1.15–1.30 (m, 6H, OCH_2_*CH*_3_ and NCH_2_*CH*_3_), 2.75 (s, 3H, CH_3_), 4.23 (q, *J* = 7.1 Hz, 2H, O*CH*_2_CH_3_), 4.40 (q, *J* = 7.3 Hz, 2H, N*CH*_2_CH_3_), 7.85 (d, *J* = 11.6 Hz, 1H, H-5), 8.70 (s, 1H, H-2).

#### Ethyl 7-bromo-1-cyclopropyl-6-fluoro-8-methyl-4-oxo-1,4-dihydroquinoline-3-carboxylate (87)

Following the same procedure used to prepare compound 86 and starting from 85, derivative 87 was obtained as a yellow solid in 67% yield (0.43 g). ^1^H NMR (200 MHz, DMSO-*d*_6_): *δ* 0.76–0.77 (m, 2H, cyclopropyl-CH_2_), 1.06–1.15 (m, 2H, cyclopropyl-CH_2_), 1.23 (t, *J* = 7.1 Hz, 3H, OCH_2_*CH*_3_), 2.89 (s, 3H, CH_3_), 4.13–4.27 (m, 3H, CH and O*CH*_2_CH_3_), 7.80 (d, *J* = 8.4 Hz, 1H, H-5), 8.62 (s, 1H, H-2).

#### Ethyl 7-(3,4-dihydroisoquinolin-2(1*H*)-yl)-1-ethyl-6-fluoro-8-methyl-4-oxo-1,4-dihydroquinoline-3-carboxylate (88)

Under inert atmosphere, a mixture of intermediate 86 (0.33 g, 0.93 mmol), Pd_2_(dba)_3_ (0.19 g, 0.23 mmol), *rac*-BINAP (0.39 g, 0.64 mmol), 1,2,3,4-tetrahydroisoquinoline (0.13 mL, 0.10 mmol), and Cs_2_CO_3_ (0.55 g, 1.70 mmol) in dry toluene (30 mL) was degassed for 30 min at rt and then refluxed for 13 h. After cooling, the reaction mixture was concentrated and poured into ice/water, the aqueous mixture was extracted with EtOAc (×3), the collected organic layers were then washed with brine, dried, and evaporated under vacuum to give a yellow solid. After crystallization by EtOH/DMF, compound 88 was obtained as a grey solid in 61% yield. ^1^H NMR (200 MHz, DMSO-*d*_6_): *δ* 1.10–1.30 (m, 6H, OCH_2_*CH*_3_ and NCH_2_*CH*_3_), 2.54 (s, 3H, CH_3_), 2.85–3.00 (m, 2H, tetrahydroisoquinoline-CH_2_), 3.45–3.55 (m, 2H, tetrahydroisoquinoline-CH_2_), 4.21 (q, *J* = 6.5 Hz, 2H, O*CH*_2_CH_3_), 4.25–4.55 (m, 4H, N*CH*_2_CH_3_ and tetrahydroisoquinoline-CH_2_), 6.98–7.29 (m, 4H, Ar–H), 7.75 (d, *J* = 12.6 Hz, 1H, H-5), 8.60 (s, 1H, H-2).

#### Ethyl 1-cyclopropyl-7-(3,4-dihydroisoquinolin-2(1*H*)-yl)-6-fluoro-8-methyl-4-oxo-1,4-dihydroquinoline-3-carboxylate (89)

Following the same procedure used to prepare compound 88 and starting from 87, derivative 89, after purification by flash column chromatography eluting CHCl_3_/MeOH 99 : 1, was obtained as an orange solid in 63% yield (0.60 g). ^1^H NMR (200 MHz, DMSO-*d*_6_): *δ* 0.75–0.80 (m, 2H, cyclopropyl-CH_2_), 1.00–1.30 (m, 5H, cyclopropyl-CH_2_ and OCH_2_*CH*_3_), 2.70 (s, 3H, CH_3_), 2.95 (t, *J* = 5.8 Hz, 2H, tetrahydroisoquinoline-CH_2_), 3.50 (t, *J* = 5.8 Hz, 2H, tetrahydroisoquinoline-CH_2_), 4.05–4.26 (m, 3H, CH and O*CH*_2_CH_3_), 4.30 (s, 2H, tetrahydroisoquinoline-CH_2_), 7.05–7.25 (m, 4H, Ar–H), 7.70 (d, *J* = 11.2 Hz, 1H, H-5), 8.60 (s, 1H, H-2).

#### 7-(3,4-Dihydroisoquinolin-2(1*H*)-yl)-1-ethyl-6-fluoro-8-methyl-4-oxo-1,4-dihydroquinoline-3-carboxylic acid (31)

General procedure A: starting from 88 (reaction time: 3 h), compound 31, after crystallization by EtOH/DMF, was obtained as a pale yellow solid in 70% yield (0.07 g). ^1^H NMR (400 MHz, DMSO-*d*_6_): *δ* 1.27 (t, *J* = 7.0 Hz, 3H, NCH_2_*CH*_3_), 2.56 (s, 3H, CH_3_), 2.80–3.00 (m, 2H, tetrahydroisoquinoline-CH_2_), 3.50–3.68 (m, 2H, tetrahydroisoquinoline-CH_2_), 4.39 (s, 2H, tetrahydroisoquinoline-CH_2_), 4.58 (q, *J* = 7.0 Hz, 2H, N*CH*_2_CH_3_), 7.00–7.20 (m, 4H, Ar–H), 7.98 (d, *J* = 12.2 Hz, 1H, H-5), 8.97 (s, 1H, H-2), 15.05 (s, 1H, CO_2_H). ^13^C NMR (100 MHz, DMSO-*d*_6_): *δ* 16.03, 19.05, 29.63, 49.48 (d, *J*_C–F_ = 5.0 Hz), 52.79, 52.92 (d, *J*_C–F_ = 5.0 Hz), 107.83, 109.65 (d, *J*_C–F_ = 23.0 Hz), 123.58 (d, *J*_C–F_ = 8.0 Hz), 126.17, 126.39, 126.61 (d, *J*_C–F_ = 4.0 Hz), 126.67, 129.53, 134.42, 134.91, 139.71, 145.19 (d, *J*_C–F_ = 12.0 Hz), 151.84, 156.68 (d, *J*_C–F_ = 249.0 Hz), 166.29, 177.11 (d, *J*_C–F_ = 2.0 Hz). HPLC r_t_: 6.2500 min. HRMS-ESI *m*/*z* [M + H]^+^ calcd for C_22_H_21_FN_2_O_3_, 381.1614, found: 381.1606.

#### 1-Cyclopropyl-7-(3,4-dihydroisoquinolin-2(1*H*)-yl)-6-fluoro-8-methyl-4-oxo-1,4-dihydroquinoline-3-carboxylic acid (32)

General procedure A: starting from 89 (reaction time: 1 h), compound 32, after crystallization by EtOH/DMF, was obtained as a yellow solid in 74% yield (0.12 g). ^1^H NMR (400 MHz, DMSO-*d*_6_): *δ* 0.85–0.92 (m, 2H, cyclopropyl-CH_2_), 1.16–1.23 (m, 2H, cyclopropyl-CH_2_), 2.71 (s, 3H, CH_3_), 2.93 (t, *J* = 5.2 Hz, 2H, tetrahydroisoquinoline-CH_2_), 3.52 (t, *J* = 5.2 Hz, 2H, tetrahydroisoquinoline-CH_2_), 4.25–4.35 (m, 1H, CH), 4.49 (s, 2H, tetrahydroisoquinoline-CH_2_), 7.10–7.20 (m, 4H, Ar-H), 7.80 (d, *J* = 12.2 Hz, 1H, H-5), 8.88 (s, 1H, H-2), 15.00 (s, 1H, CO_2_H). ^13^C NMR (100 MHz, DMSO-*d*_6_): *δ* 10.95, 19.05, 29.67, 41.53, 49.57 (d, *J*_C–F_ = 5.0 Hz), 52.90 (d, *J*_C–F_ = 5.0 Hz), 107.43, 109.32 (d, *J*_C–F_ = 23.0 Hz), 122.95 (d, *J*_C–F_ = 8.0 Hz), 126.17, 126.47, 126.67, 127.23 (d, *J*_C–F_ = 4.0 Hz), 129.52, 134.44, 134.91, 141.01, 145.20 (d, *J*_C–F_ = 12.0 Hz), 152.54, 156.61 (d, *J*_C–F_ = 248.5 Hz), 166.11, 177.20 (d, *J*_C–F_ = 2.0 Hz). HPLC r_t_: 6.5300 min. HRMS-ESI *m*/*z* [M + H]^+^ calcd for C_23_H_21_FN_2_O_3_, 393.1614, found: 393.1609.

#### Ethyl 1-cyclopropyl-7-(3,4-dihydroisoquinolin-2(1*H*)-yl)-6-fluoro-4-oxo-1,4-dihydroquinoline-3-carboxylate (91)

General procedure B: starting from 90 (ref. 43) and using 1,2,3,4-tetrahydroisoquinoline (3 equiv.) as a nucleophile (reaction time: 4 h; temperature: 100 °C; solvent: DMSO), compound 91 was obtained as a pale yellow solid in 67% yield (0.23 g). ^1^H NMR (400 MHz, DMSO-*d*_6_): *δ* 1.00–1.05 (m, 2H, cyclopropyl-CH_2_), 1.19–1.21 (m, 2H, cyclopropyl-CH_2_), 1.26 (t, *J* = 7.0 Hz, 3H, OCH_2_*CH*_3_), 2.91–2.97 (m, 2H, tetrahydroisoquinoline-CH_2_), 3.59–3.63 (m, 3H, tetrahydroisoquinoline-CH_2_ and cyclopropyl-CH), 4.19 (q, *J* = 7.0 Hz, 2H, O*CH*_2_CH_3_), 4.51 (s, 2H, tetrahydroisoquinoline-CH_2_), 7.18–7.26 (m, 4H, Ar–H), 7.48 (d, *J* = 6.7 Hz, 1H, H-8), 7.77 (d, *J* = 13.5 Hz, 1H, H-5), 8.40 (s, 1H, H-2).

#### 1-Cyclopropyl-7-(3,4-dihydroisoquinolin-2(1*H*)-yl)-6-fluoro-4-oxo-1,4-dihydroquinoline-3-carboxylic acid (33)

General procedure A: starting from 91 (reaction time: 4 h), compound 33, after crystallization by EtOH/DMF, was obtained as a yellow solid in 49% yield (0.10 g). ^1^H NMR (400 MHz, DMSO-*d*_6_): *δ* 1.00–1.08 (m, 2H, cyclopropyl-CH_2_), 1.18–1.25 (m, 2H, cyclopropyl-CH_2_), 2.94–2.96 (m, 2H, tetrahydroisoquinoline-CH_2_), 3.68 (t, *J* = 5.2 Hz, 2H, tetrahydroisoquinoline-CH_2_), 3.71–3.75 (m, 1H, CH), 4.56 (s, 2H, tetrahydroisoquinoline-CH_2_), 7.17–7.25 (m, 4H, Ar–H), 7.55 (d, *J* = 7.3 Hz, 1H, H-8), 7.85 (d, *J* = 13.5 Hz, 1H, H-5), 8.58 (s, 1H, H-2), 15.21 (s, 1H, CO_2_H). ^13^C NMR (100 MHz, DMSO-*d*_6_): *δ* 7.90, 29.40, 36.18, 48.25 (d, *J*_C–F_ = 7.0 Hz), 51.32, 106.02, 107.01, 111.38 (d, *J*_C–F_ = 23.0 Hz), 118.29 (d, *J*_C–F_ = 7.0 Hz), 126.52, 126.85, 127.00, 129.00, 133.85, 134.54, 139.65, 144.87 (d, *J*_C–F_ = 10.0 Hz), 148.27, 153.03 (d, *J*_C–F_ = 247.0 Hz), 166.37, 176.67. HPLC r_t_: 6.9470 min. HRMS-ESI *m*/*z* [M + H]^+^ calcd for C_22_H_19_FN_2_O_3_, 379.14527, found: 379.1464.

### Cell handling and treatment

SKOV-3, A2780 and A2780 CIS were cultured and maintained, as previously described,^50^ at 37 °C in RPMI 1640 medium (Euroclone) supplemented with 10% fetal bovine serum (FBS; Euroclone), l-glutamine and penicillin/streptomycin (SIGMA-ALDRICH). OVCA433, HEK-293T, HeLa, HCCLM3, SK-Hep-1, MCF-7, A549 and HCT-116 cells were maintained in DMEM medium (Euroclone) containing 10% FBS (Euroclone), l-glutamine and penicillin/streptomycin (SIGMA-ALDRICH). Wi-38 cells were cultured in DMEM medium (Euroclone) supplemented with 10% FBS (Euroclone), l-glutamine and penicillin/streptomycin (SIGMA-ALDRICH), and 1% non-essential amino acids (NEAA; Euroclone). The cultures were maintained in a constant temperature incubator at 37 °C with 5% CO_2_.

For the evaluation of GI_50_ and CC_50_, cells were counted and plated in equal numbers, amounting to 100 000 cells in 35 mm culture dish. Once cell adhesion had been promoted, the compounds were added to the culture medium. After 72 hours of treatment, the cells were counted, and the data analyzed by linear regression. Each experiment was carried out three times in triplicate and three separate cell counts were made for each sample.

HEK-293T_shSCR and HEK-293T_shGAPDH were treated with 50 μM of enoxacin and 33 for 48 hours before RNA extraction and expression analysis.

HeLa cells were transfected with 10 ng of psi-R21 or psi-CTRL using Lipofectamine™ 2000 (Thermo Fisher Scientific) and after 48 hours were treated with either enoxacin or 33 at 20 μM and 50 μM. After 6 hours Renilla luciferase (RLuc) and firefly luciferase (FLuc) activities were measured using the Dual-Luciferase® Reporter Assay System (Promega), following the manufacturer's instructions, and GloMax® Discover Microplate Reader (Promega).

HCCLM3 and SK-Hep-1 cells were seeded into 96-well plates at a density of 10 000 cells per well and allowed to adhere overnight. Subsequently, the cells were treated with the following substances: 0.1% DMSO as the negative control, various concentrations of the test compounds, and 125 μM enoxacin as the positive control. After 48 h of incubation, the original culture medium was removed, and the cells were washed twice with PBS. Then, 100 μL of DMEM culture medium containing 10% CCK-8 (Bimake, USA) was added to each well. The plates were incubated for 30 minutes at 37 °C. To determine the fluorescence intensity, measurements were taken at a wavelength of 450 nm using a Varioskan from Thermo Fisher Scientific.

A2780 and SKOV-3 cells were treated with 50 μM of 33 for 72 h before RNA and protein extraction and expression analyses. A2780 and A2780 CIS were treated with 33 (30 μM) and/or cisplatin (1 μM, Sigma-Aldrich) for 48 hours while MCF-7, A549 and HCT-116 were treated with 33 (30 μM) for 72 hours. Total cell number and viability have been assessed using Trypan Blue Solution (Thermo Fisher Scientific) and Countess™ 3 Automated Cell Counter (Thermo Fisher Scientific).

### Equation to evaluate GI_50_ and CC_50_

The formula suggested by GraphPad titled ‘Absolute IC_50_, X is the concentration’ was used to assess GI_50_ in SKOV-3, OVCA433 and A2780 cell lines and CC_50_ in Wi-38 cell lines.

In detail, the formula is as follows:Fifty = (Top + Baseline)/2*Y* = Bottom + (Top − Bottom)/(1 + ((Top − Bottom)/(Fifty − Bottom) − 1) × (Absolute IC_50_/*X*)^HillSlope)Considering: *X* = Compound concentration; *Y* = number of cellsEach compound was initially tested in a wide range of concentrations, up to 200 μM (in SKOV-3, OVCA433 and A2780) and up to 250 μM (in Wi-38). Afterwards, the concentration range was narrowed to the relative value obtained in the first evaluation in order to obtain an accurate value.

The confidence intervals (CI) of the parameters were calculated with a 95% confidence level. The goodness-of-fit was quantified by means of *R* squared and sum-of-squares.

### Surface plasmon resonance (SPR) assay

Following the manufacturer's instructions, the BIAcore Sensor Chip (CM5) underwent initial activation using a mixture of NHS and EDC. Subsequently, recombinant human TRBP proteins at a concentration of 20 μg mL^−1^ (in 10 mM sodium acetate, pH 4.0) were injected over the CM5 chip. After reaching a coupling level of 12 000 RU, a 1 M ethanolamine hydrochloride was injected to block the chip, preventing nonspecific binding. All the compounds were diluted in HBS-EP buffer (10 mM HEPES, pH 7.4, 3 mM EDTA, 150 mM NaCl, and 0.005% surfactant P-20) with 1% DMSO. Samples were injected at a rate of 10 μL min^−1^ for 120 seconds, followed by a dissociation phase of 90 seconds. After each cycle, a 50% DMSO solution was used to regenerate the chip surface and restore the sensorgram baseline. Binding analysis was performed using BIA evaluation version 3.2 software, determining the dissociation rate constant (*K*_D_).

### Plasmid construction

The binding site for miR-21 was cloned into psiCHECK™-2 Vector (Promega), downstream the Renilla cDNA, by inverted PCR using the following oligonucleotides: 21MRE_FW CTGATAAGCTA AACCTAGAGCGGCCGCTGGC; 21MRE_REV ACTGATGTTGA GGCTCGAGCGATCGCCTAGAA. The resulting construct was named psi-R21.

### Lentivirus packaging and viral transduction

Lentiviral particles were produced by calcium phosphate transient transfection of HEK-293T cells with specific lentiviral plasmids (shGAPDH: TRCN0000445616 and shSCR: TRCN0000015937 from SIGMA-ALDRICH) together with the packaging plasmids (pLP1and pLP2) and the envelope plasmid pLP/VSVG. For each lentiviral plasmid one 150 mm plate of HEK293T cells was used for transfection. After 14–16 hours the medium was replaced with complete medium supplemented with 1 mM sodium butyrate (SIGMA-ALDRICH). The collection of medium containing lentiviral particles occurs 48 h and 72 h after transfection; complete medium supplemented with sodium butyrate was added after the first collection. Collected media were pulled and centrifuged at 1000 rpm for 5 min at room temperature. The supernatant was filtered through 0.45 μm pore nitrocellulose filters and then ultracentrifuged at 20 000 rpm for 2 hours at 4 °C. The pellet containing lentiviral particles was then resuspended in 25 μL HBSS buffer (GIBCO ThermoFisher) and stored at −80 °C.

1 × 10^6^ of HEK-293T cells were resuspended in 1 ml of serum-free and antibiotic-free medium supplemented with polybrene at 4 μg ml^−1^ and infected with 5 μL of the lentiviral particles in HBSS buffer. After 6 h one volume of medium with FBS 2% and penicillin/streptomycin 2× was added and after additional 24 h the medium was replaced with complete medium. 48 h after viral transduction, HEK-293T_shSCR and HEK-293T_shGAPDH transduced cells were selected using 3 μg mL^−1^ of puromycin (SIGMA-ALDRICH).

### RNA extraction and analysis

Total RNA was extracted using the Direct-zol RNA MiniPrep kit (Zymo Research) with on-column DNase treatment, according to the manufacturer's instructions. Reverse transcription was carried out with SuperScript VILO cDNA Synthesis Kit (Life Technologies) and the cDNA samples were analyzed by qRT-PCR using PowerUp SYBR Green Master Mix (Thermo Fisher Scientific). For miR-21 expression analysis total RNA was retrotranscribed using miRCURY® LNA® RT Kit (Qiagen) and the cDNA was analyzed by using miRCURY® LNA® miRNA PCR Assay (Qiagen). The oligonucleotides used are the following: GAPDH_FW ACCCACTCCTCCACCTTTGA; GAPDH_REV TCCACCACCCTGTTGCTGTA; ATP5O_FW ACTCGGGTTTGACCTACAGC; ATP5O_REV GGTACTGAAGCATCGCACCT; hsa-miR-21-5p miRCURY LNA miRNA PCR Assay (YP00204230); U6 snRNA (v2) miRCURY LNA miRNA PCR Assay (YP02119464).

### Cell thermal shift assay

HEK-293T cells were harvested in PBS supplemented with protease inhibitor cocktail (complete, EDTA-free, Roche). Cell suspensions were freeze-thawed three times in liquid nitrogen and then centrifuged (20 000*g*) for 10 min at 4 °C. The lysates were diluted at concentration of 2 μg μL^−1^ and then treated with DMSO and 50 μM of 33 or with increasing concentration of 33 for 30 min at 37 °C. After this incubation the lysates were divided into equal parts (50 μL) and heated individually at designated temperatures for 3 min. The heated samples were cooled at room temperature for 3 min and centrifuged (20 000*g*) for 10 min at 4 °C. The supernatants were transferred to new tubes and analyzed by use for western blotting.

### Western blotting

Lysates were loaded on 4–12% bis–tris-acrylamide gel (Life Technologies) and transferred to a nitrocellulose membrane. The membrane was blocked in 5% milk and hybridized with α-TRBP (15753-1-AP Proteintech), α-GAPDH (sc-25778, Santa Cruz Biotechnology), α-c-PARP (Bioss Antibodies, BSM-52408R), α-BCLX_L_ (mAb #2764, Cell Signaling Technology) and α-β-actin (A3854, Sigma) antibodies. All the images were captured using the ChemiDocTM Touch Imaging System (Bio-Rad), and the densitometric analyses were performed using the associated Image Lab software (Bio-Rad).

### Detection of hTOP1 and hTOP2α cleavage complexes (TOP1ccs and TOP2ccs) *in vivo*

After compound treatments, 1 × 10^6^ human cells in 35 mm dish per sample were washed with 1× phosphate-buffered saline (PBS) and lysed with 600 μL of DNAzol (Invitrogen), followed by precipitation with 300 μL of 200 proof ethanol. The nucleic acids were collected, washed with 75% ethanol, resuspended in 200 μL of TE buffer, and then heated at 65 °C for 15 min, followed by shearing with sonication (40% output for 10 s pulse and 10 s rest for four times). The samples were centrifuged at 15 000 rpm for 5 min at 4 °C, and the supernatant were collected. 1 μL of each sample was removed for spectrophotometric measurement of absorbance at 260 nm to quantitate DNA content (NanoDrop). 2 μg of each sample was subjected to slot-blot for immunoblotting using anti-hTOP1 antibody (BD Pharmingen, 556597) and anti-hTOP2α antibody (Santa Cruz biotechnology, 365916).^51^

## Abbreviation list

GI_50_: 50% growth inhibition
HGSC: High-grade serous ovarian carcinoma
KO: Knock out
OC: Ovarian cancer
SMER: Small-molecule enhancer of miRNA
SPR: Surface plasmon resonance
Tops: Topoisomerases
TRBP: TAR RNA-binding protein
WT: Wild type

## Author contributions

Conceptualization: T. F., N. D. I., M. M., G. S., G. M.; validation: T. F., N. D. I., M. A. D. F., S. P., J. Y., Y. S.; formal analysis and investigation: T. F., N. D. I., D. P.; S. P., M. P., J. Y., Y. S., S. M., M. L. B., S. S., O. T., V. C., M. M.; resources: M. M., G. S., G. M.; writing – original draft: T. F., N. D. I.; writing – review & editing: T. F., N. D. I., M. A. D. F., V. C., F. W., Y. P., M. M., G. S., G. M.; supervision: V. C., F. W., Y. P., M. M., G. S., G. M.; project administration: M. M., G. S., G. M.

## Conflicts of interest

The authors declare that they have no known competing financial interests or personal relationships that could have appeared to influence the work reported in this paper.

## Supplementary Material

MD-016-D4MD00649F-s001

## Data Availability

The data supporting this article have been included as part of the ESI.†
