# Supplementary material for: New anti-ovarian cancer quinolone derivatives acting by modulating microRNA processing machinery
Source: RSC Med Chem. 2024 Sep 27;16(1):98–124. doi: 10.1039/d4md00649f (PMC11467779; doi:10.1039/d4md00649f)

Supplementary Information (SI) for RSC Medicinal Chemistry.
This journal is © The Royal Society of Chemistry 2024

Supplementary Information

New anti-ovarian cancer quinolone derivatives acting by modulating microRNA processing machinery

Tommaso Felicetti,[$,a] Nicola Di Iacovo,[$,b] Maria Agnese Della-Fazia,[b] Danilo Piobbico,[b] Stefania Pieroni,[b] Martina Pacetti,[c] Jialing Yu,[d] Yilun Sun,[e] Serena Massari,[a] Maria Letizia Barreca,[a] Stefano Sabatini,[a] Oriana Tabarrini,[a] Violetta Cecchetti,[a] Fei Wang,[d] Yves Pommier,[e] Mariangela Morlando,*[,a,c] Giuseppe Servillo,*[,b] Giuseppe Manfroni.*[,a]

[a]Department of Pharmaceutical Sciences, Section of Chemistry and Pharmaceutical Technology, University of Perugia, Via Del Liceo, 1-06123, Perugia, Italy

[b]Department of Medicine and Surgery, University of Perugia, Piazza L. Severi, 1/8-06132, Perugia, Italy

[c]Current address: Department of Biology and Biotechnologies "C. Darwin", Sapienza University of Rome, P.le Aldo Moro 5, 00185, Rome, Italy

[d]Center for Natural Products Research, Chengdu Institute of Biology, Chinese Academy of Sciences, Chengdu 610041, China

[e]Center for Cancer Research, Developmental Therapeutics Branch & Laboratory of Molecular Pharmacology, NCI, 31 Center Drive, Bethesda, MD 20892-4255, United States

[$]Authors contributed equally to this work

*Corresponding authors

M.M.: mariangela.morlando@uniroma1.it, 0039 06 4991 2341; G.S.: giuseppe.servillo@unipg.it, 0039 075 585 8110; G.M.: giuseppe.manfroni@unipg.it, 0039 075 585 5126.

Table of Contents

Figure S1. hTop1 and hTop2α cleavage assay of compounds **5** and **33** Pag. S3

Figure S2. **33** inhibits the proliferation of MCF-7, A549 and HCT-116 cell lines Pag. S3

Figures S3-S48. ^{1}H NMR and ^{13}C NMR spectra of compounds **11-33**. Pag. S4-S26

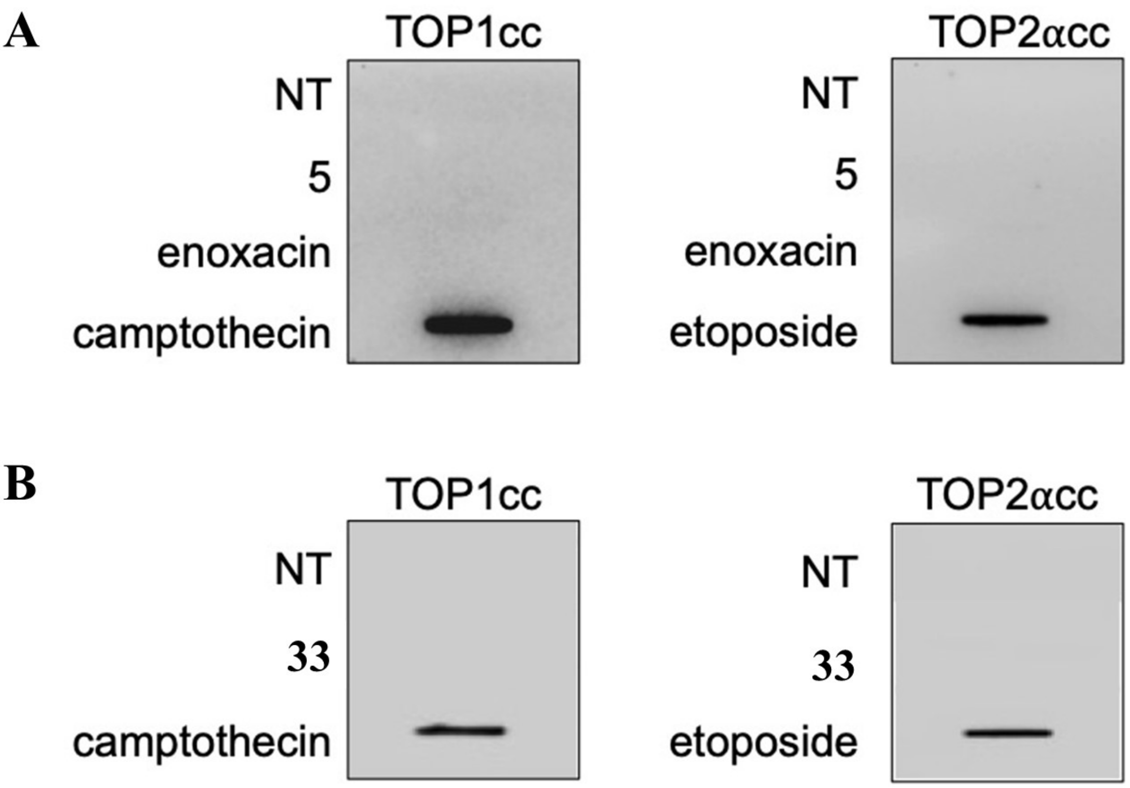

Figure S1. hTop1 and hTop2α cleavage assay of compounds **5** and **33**

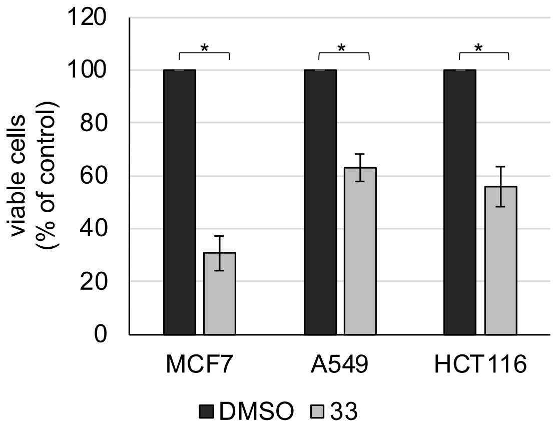

Figure S2. 33 inhibits the proliferation of MCF-7, A549 and HCT-116 cell lines. Graph showing the number of viable cells of a culture of MCF-7, A549 and HCT-116 cell lines treated with **33** (30 μM) for 72 hours. The number of viable cells is expressed as percentage respect to DMSO condition set to a value of 100%. Error Bars represent SE of at least three independent experiments. p-values were calculated using paired two-tailed Student's t-test (* $p < 0.05$).

Figure S3. ^{1}H NMR spectrum of compound **11**.

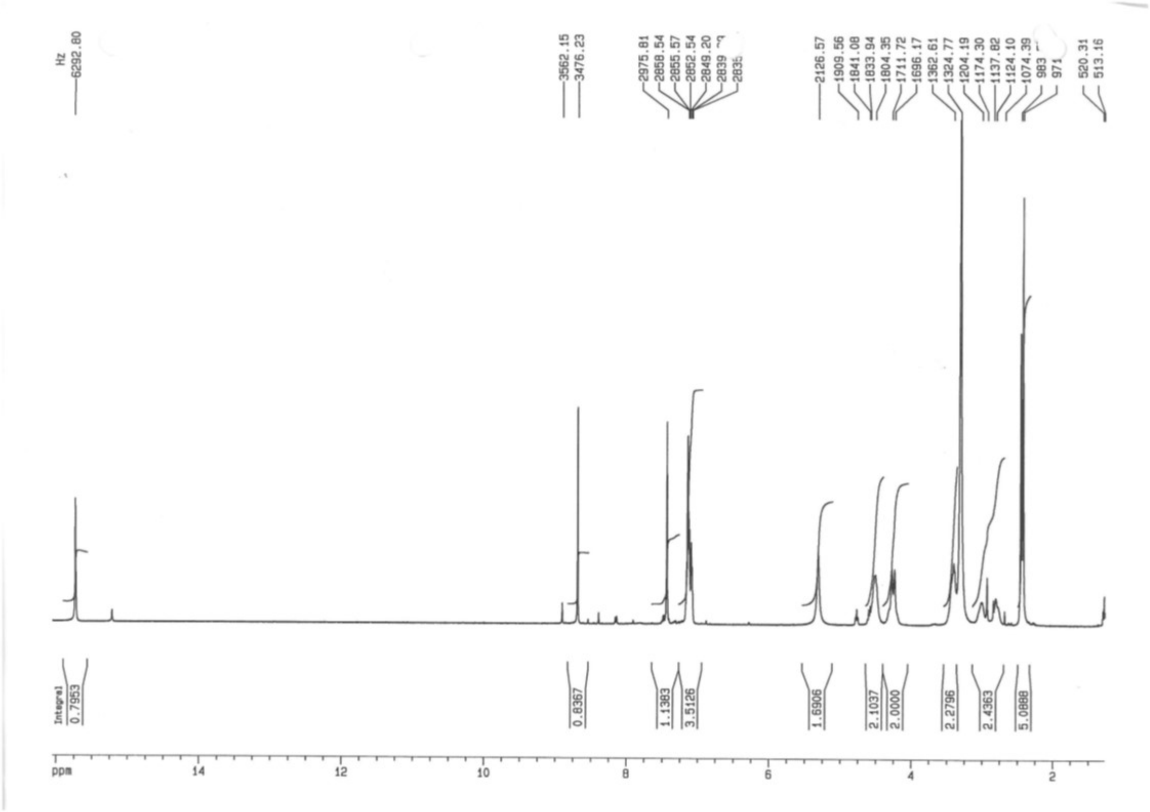

Figure S4. ^{13}C NMR spectrum of compound **11**

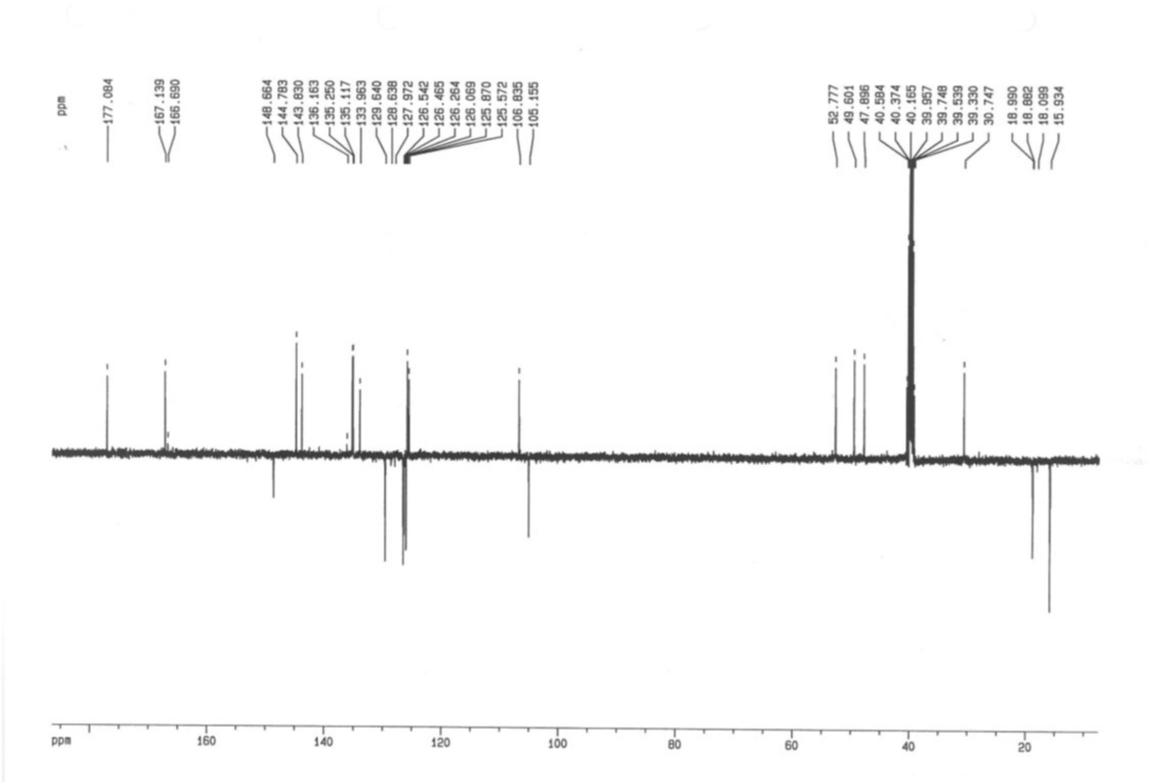

Figure S5. ^{1}H NMR spectrum of compound **12**.

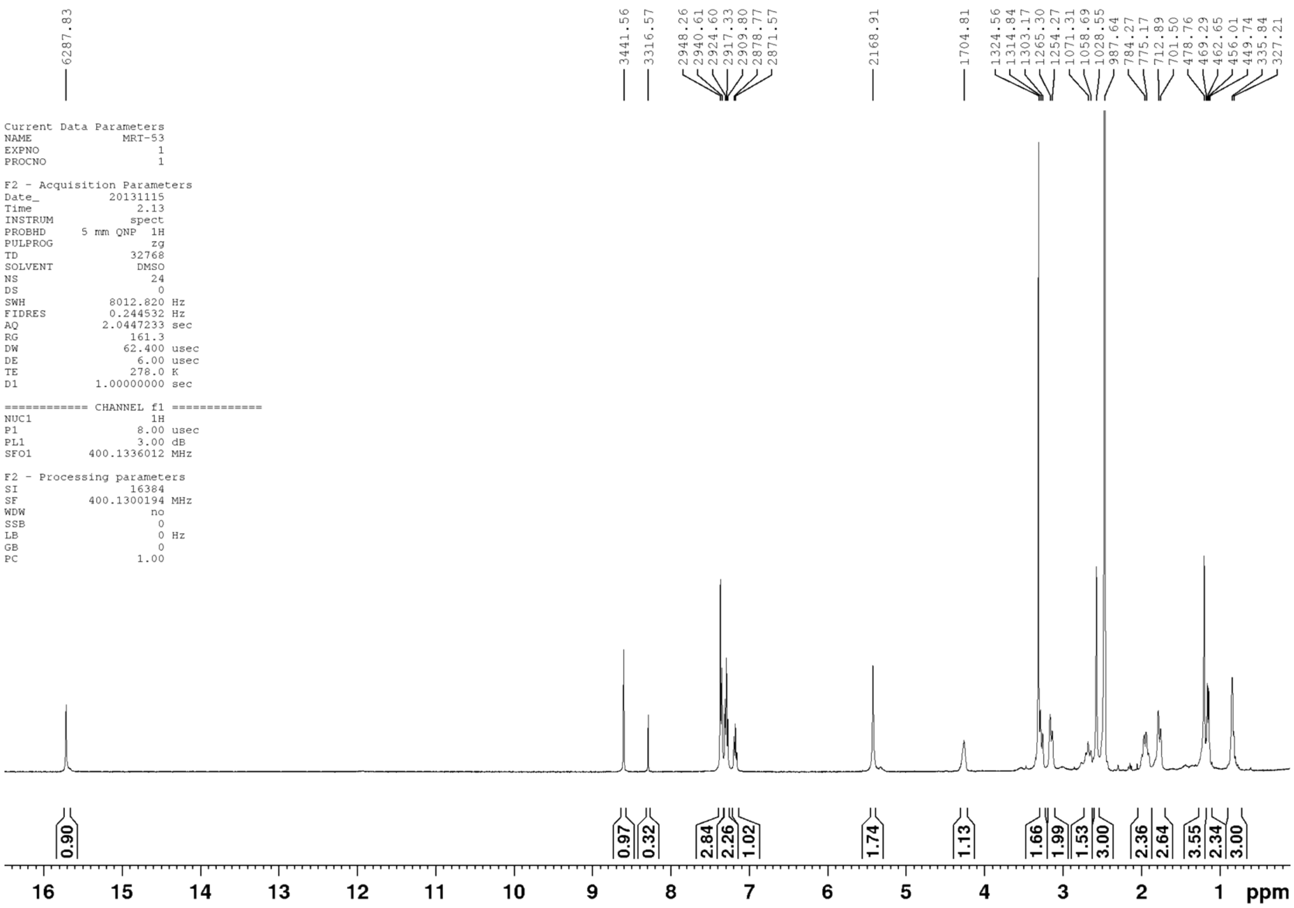

Figure S6. ^{13}C NMR spectrum of compound **12**.

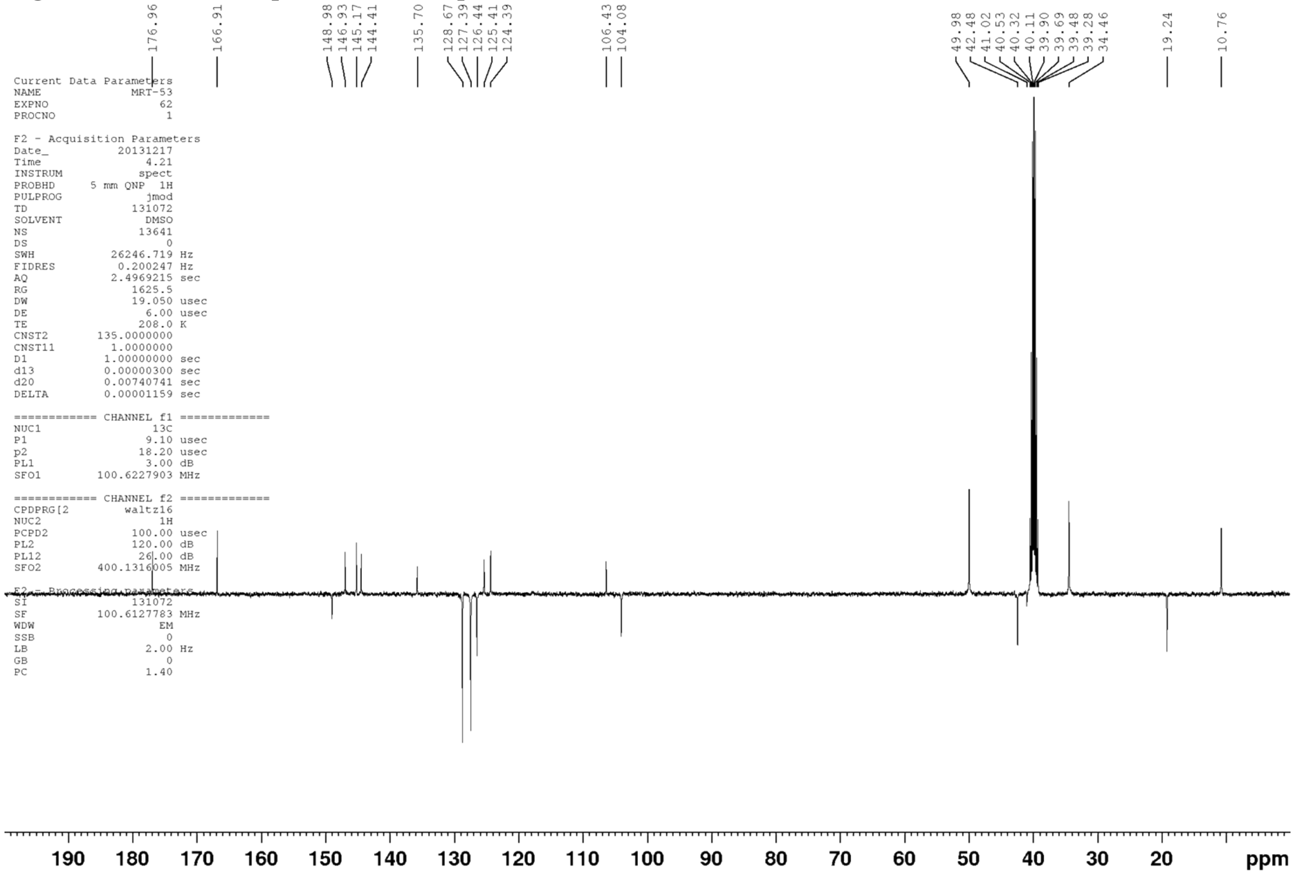

Figure S7. ^{1}H NMR spectrum of compound **13**.

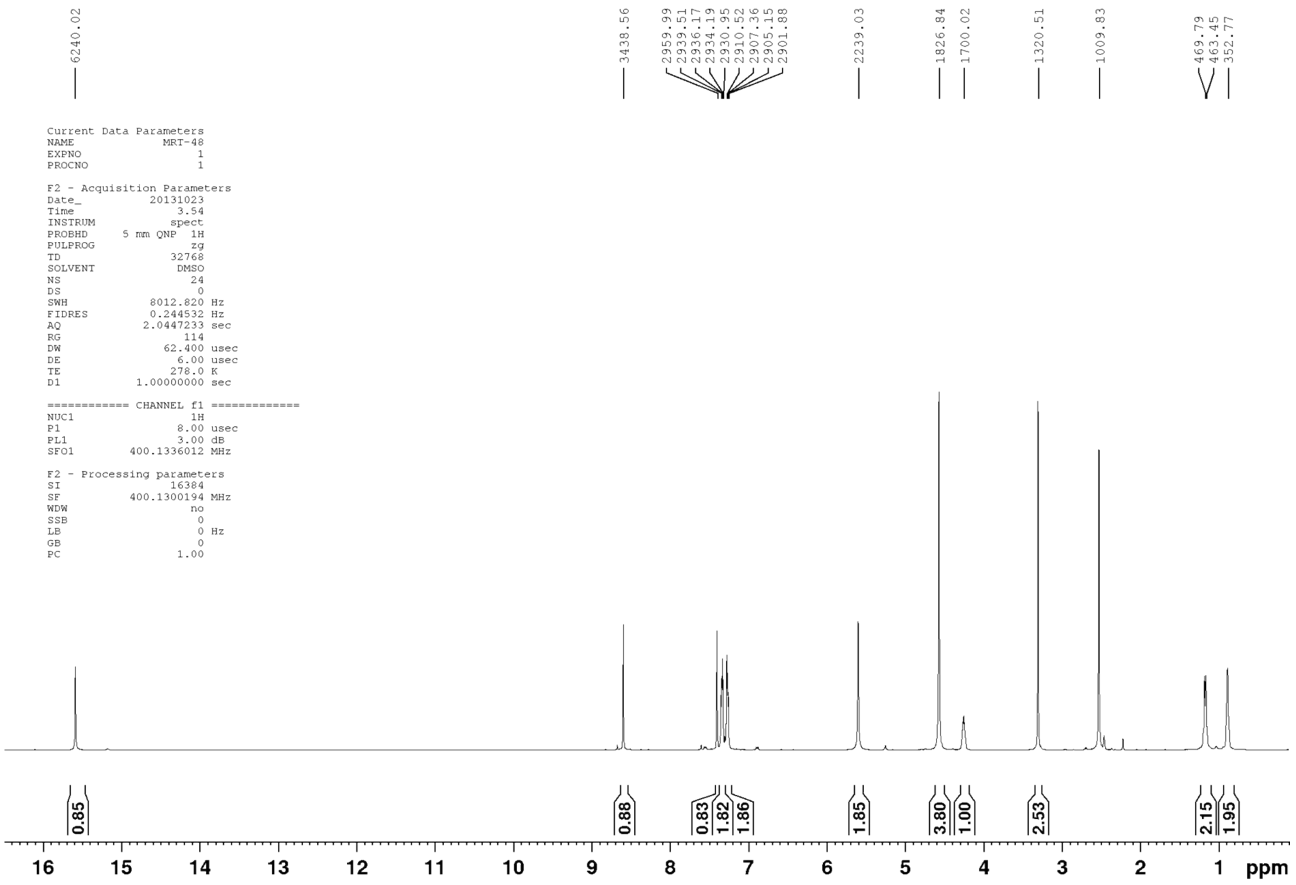

Figure S8. ^{13}C NMR spectrum of compound **13**.

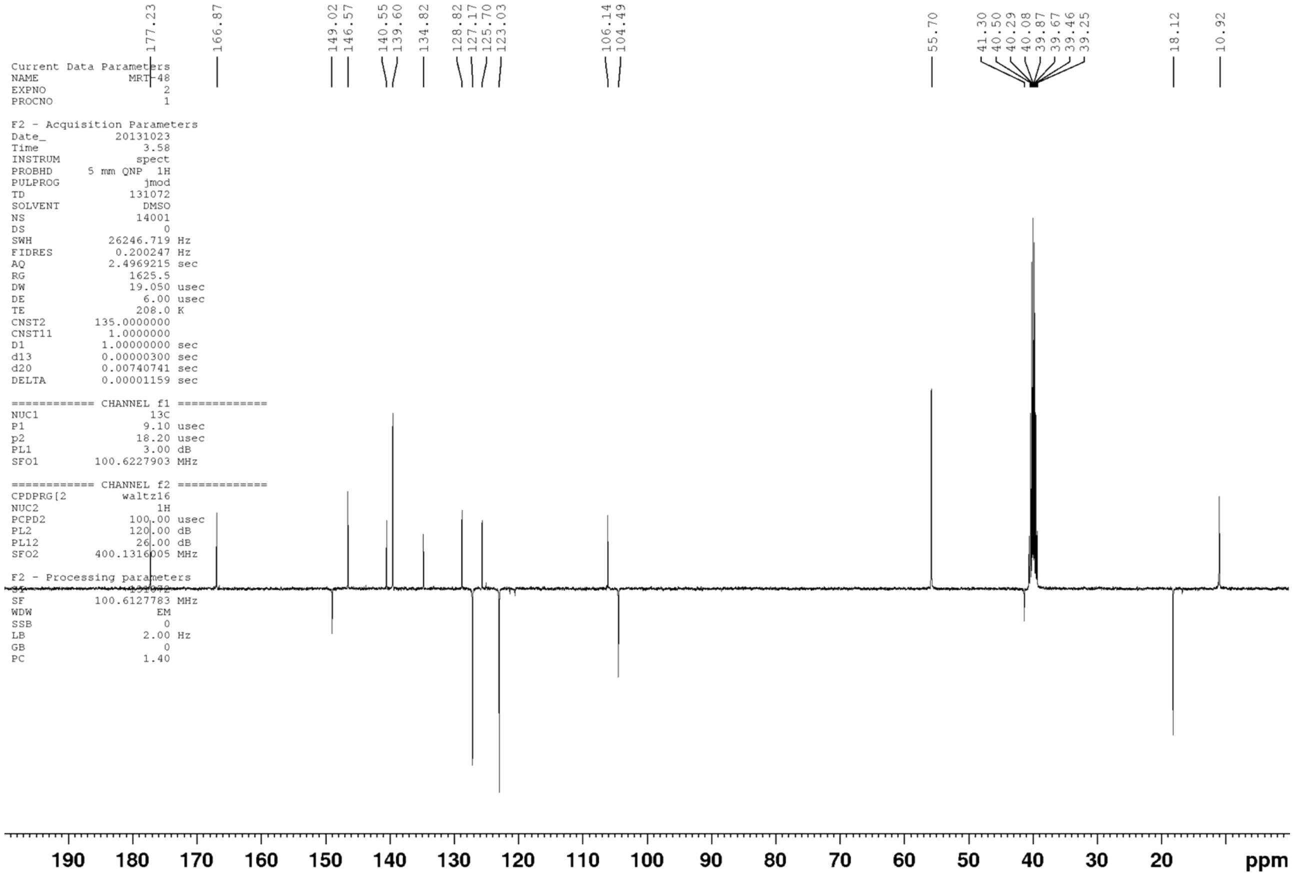

Figure S9. ^{1}H NMR spectrum of compound **14**.

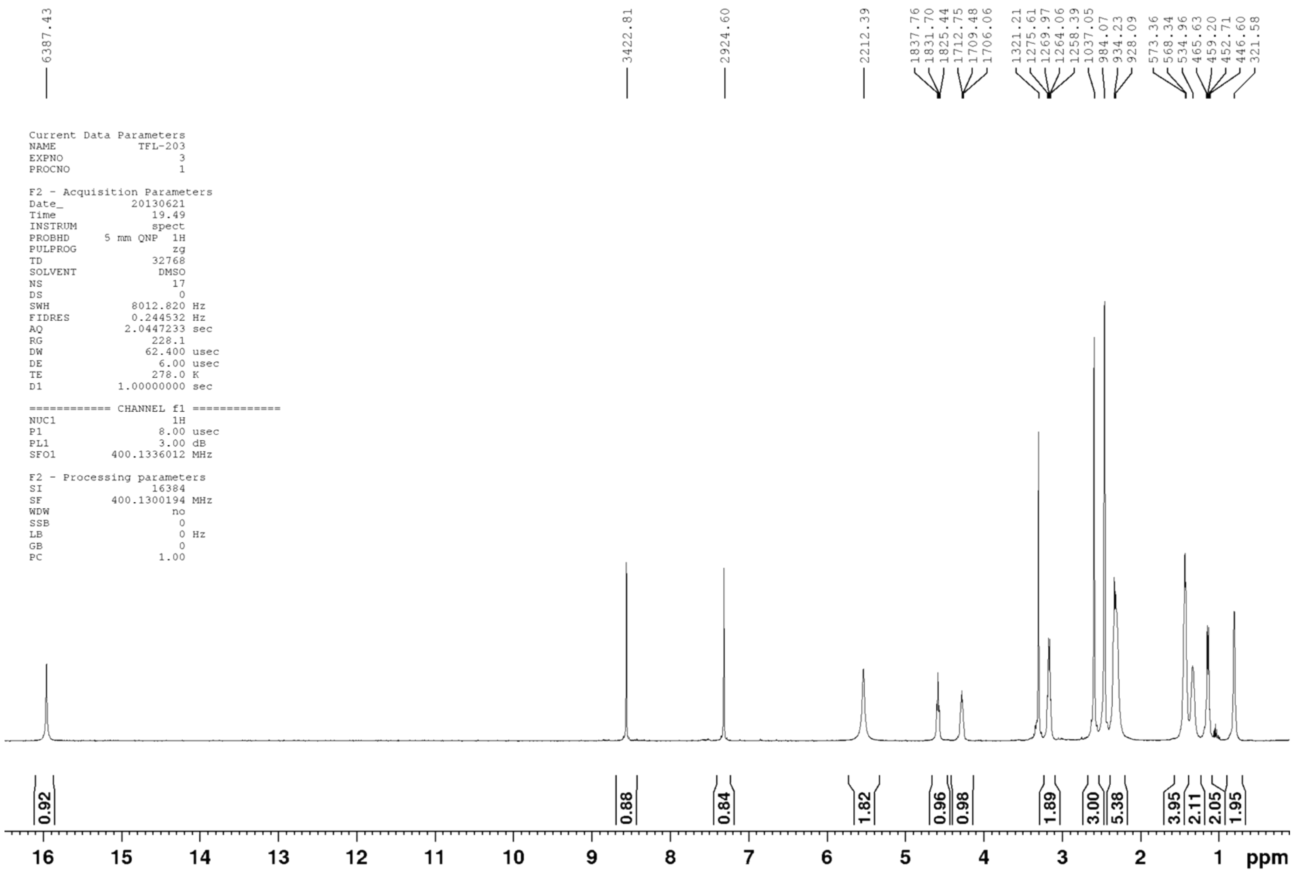

Figure S10. ^{13}C NMR spectrum of compound **14**.

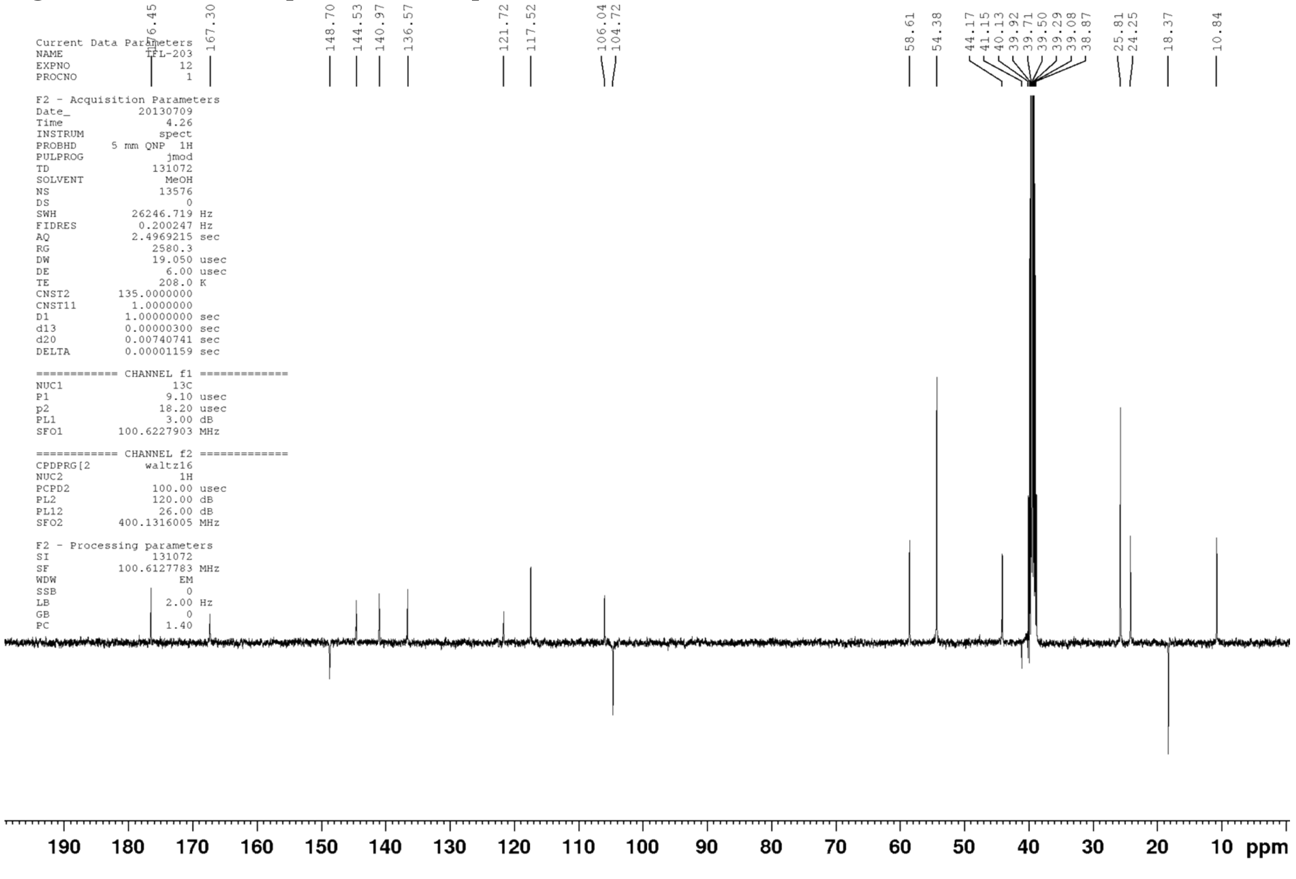

Figure S11. ^{1}H NMR spectrum of compound **15**.

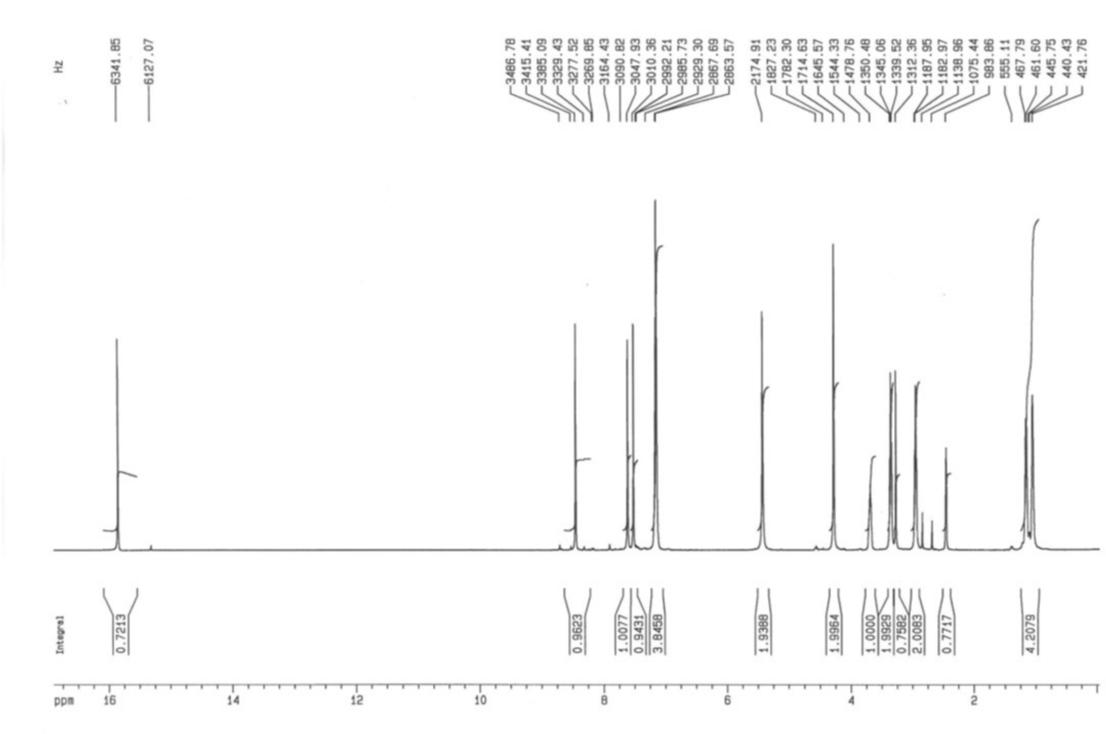

Figure S12. ^{13}C NMR spectrum of compound **15**.

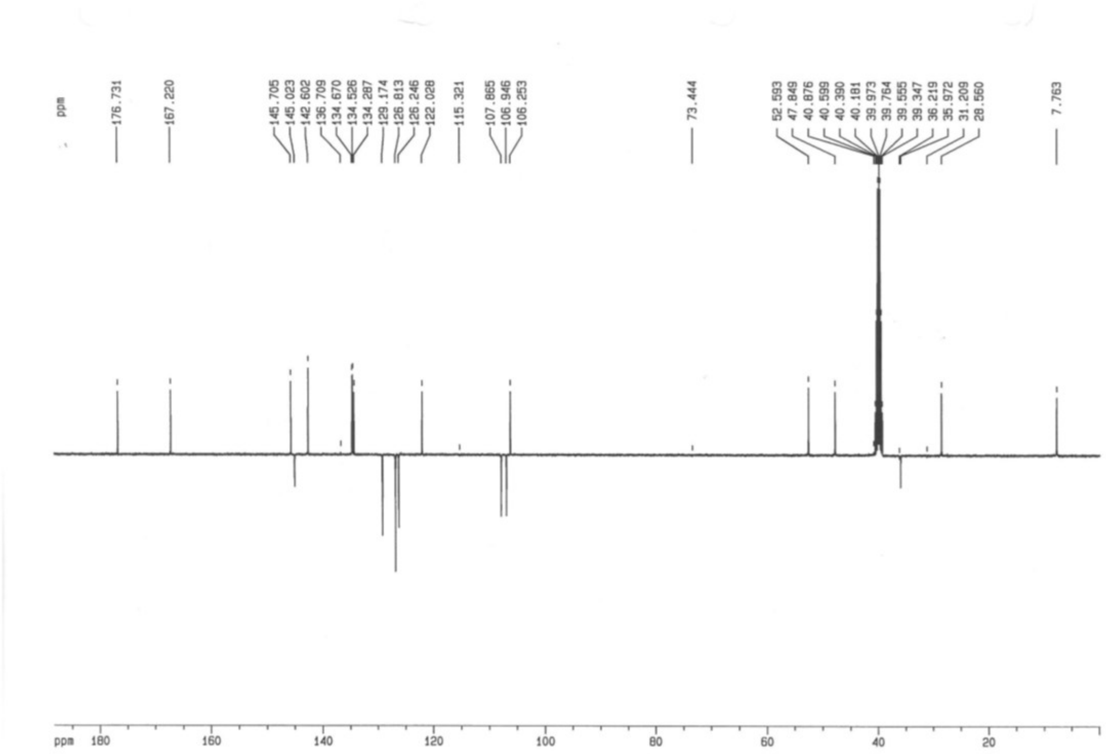

Figure S13. ^{1}H NMR spectrum of compound **16**.

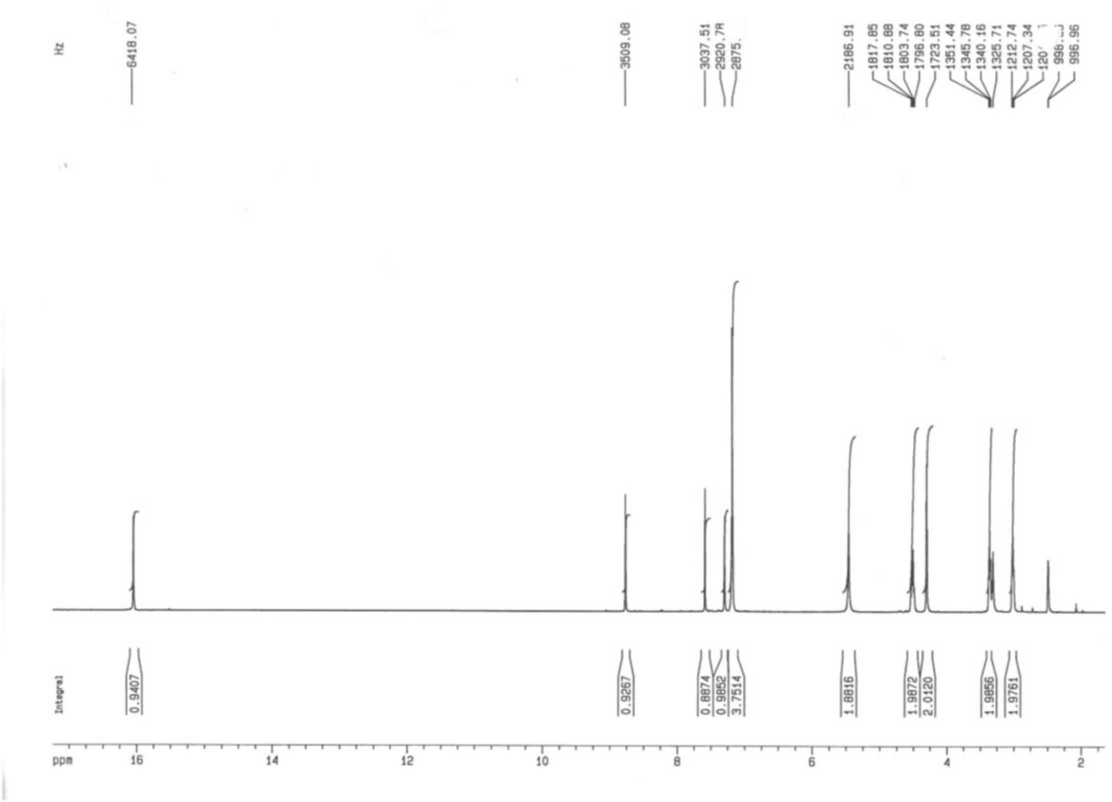

Figure S14. ^{13}C NMR spectrum of compound **16**.

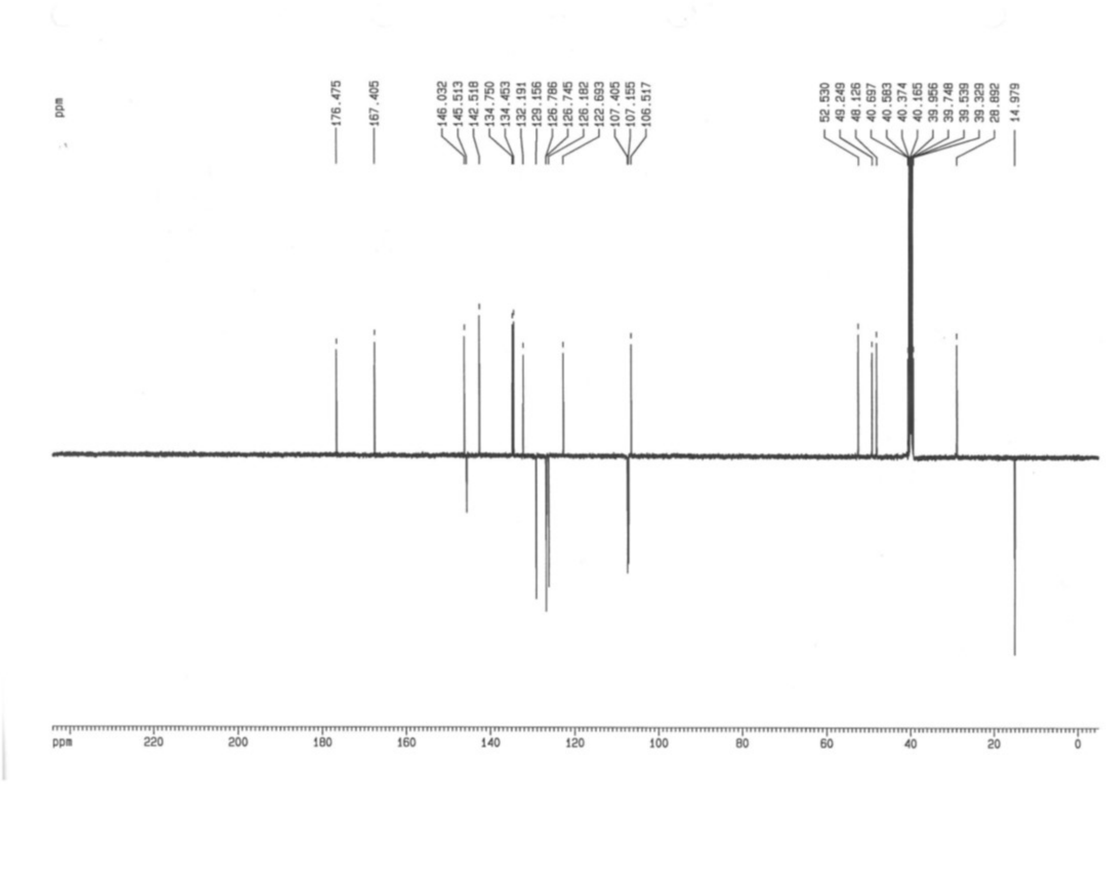

Figure S15. ^{1}H NMR spectrum of compound **17**.

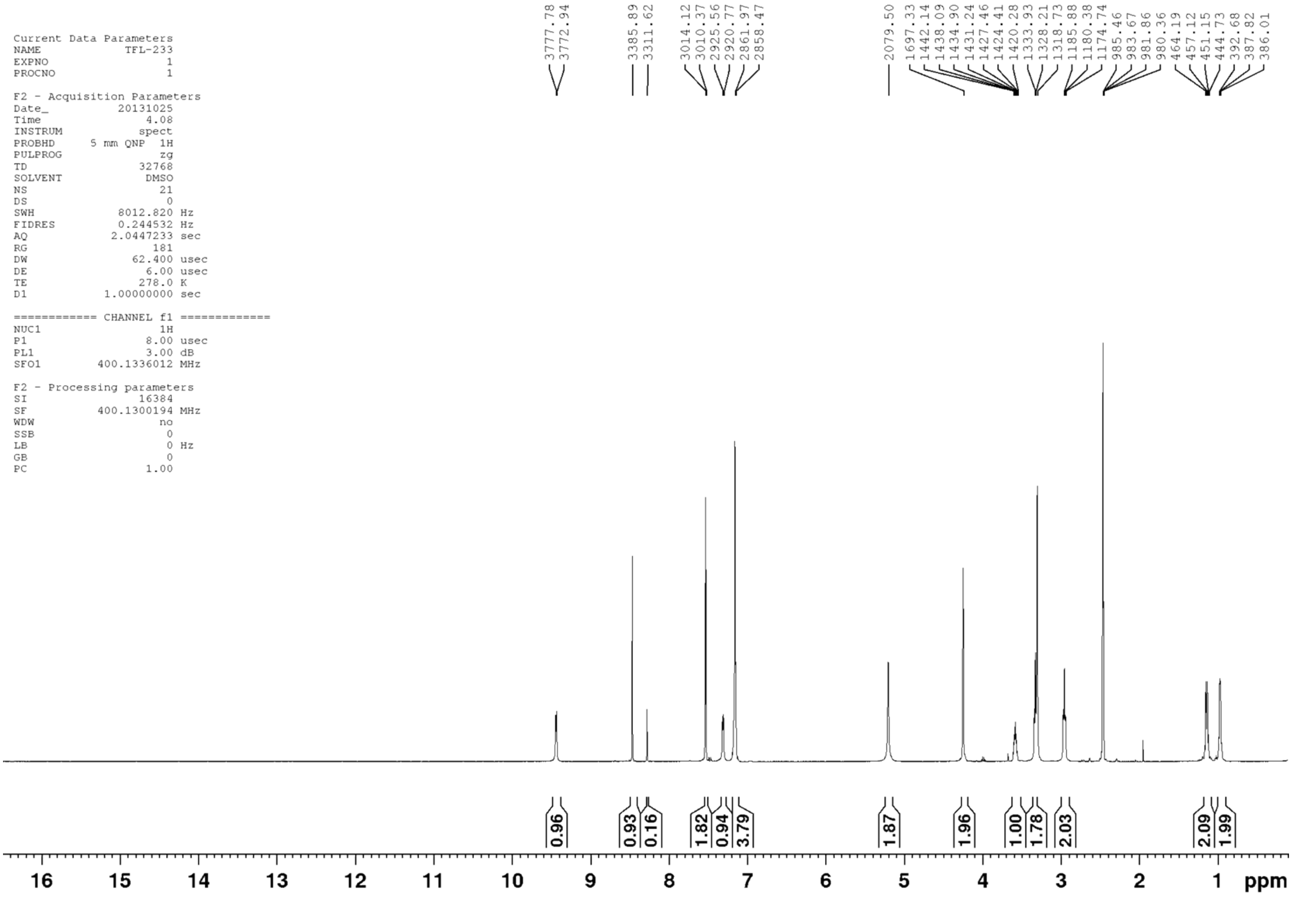

Figure S16. ^{13}C NMR spectrum of compound **17**.

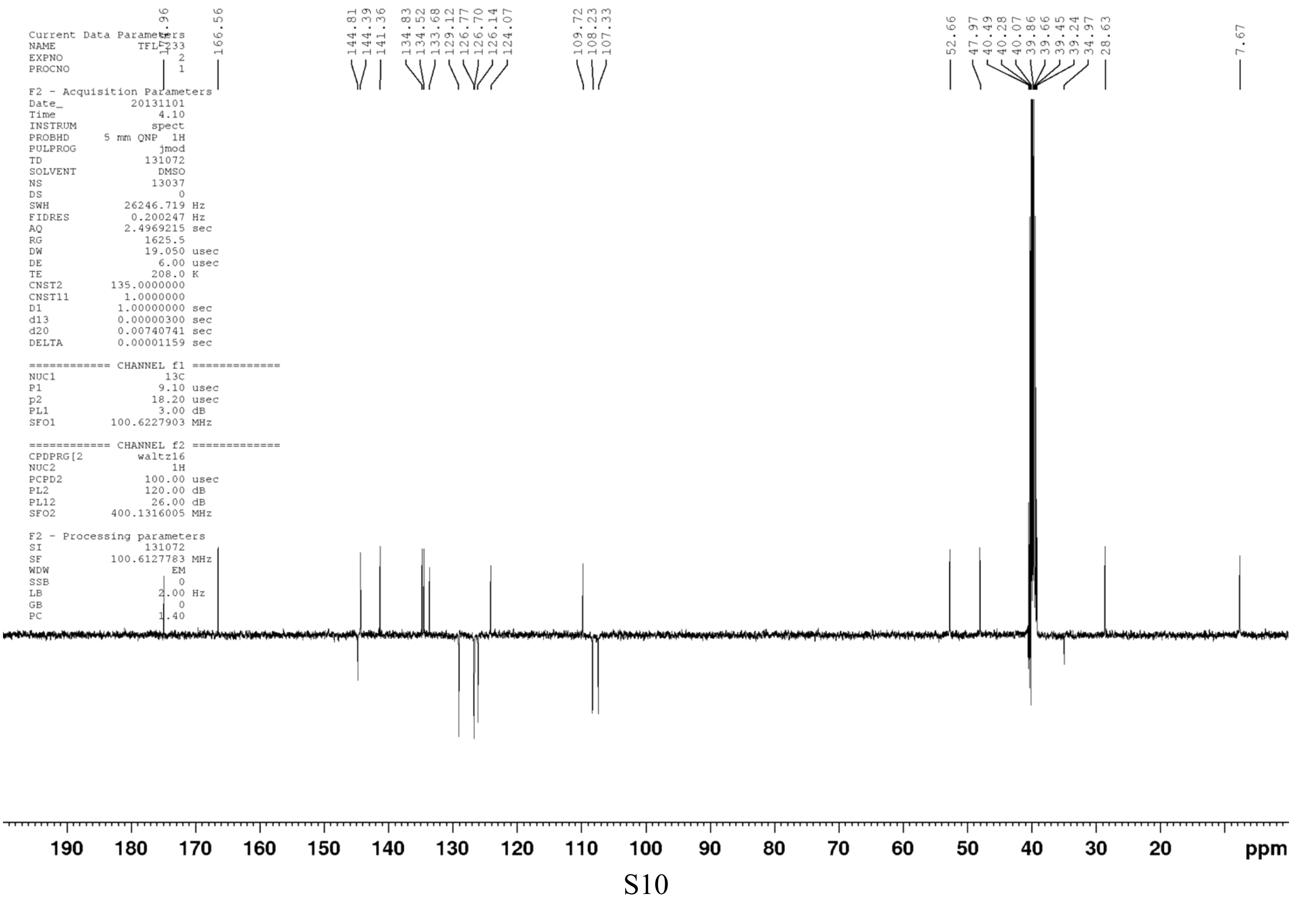

Figure S17. ^{1}H NMR spectrum of compound **18**.

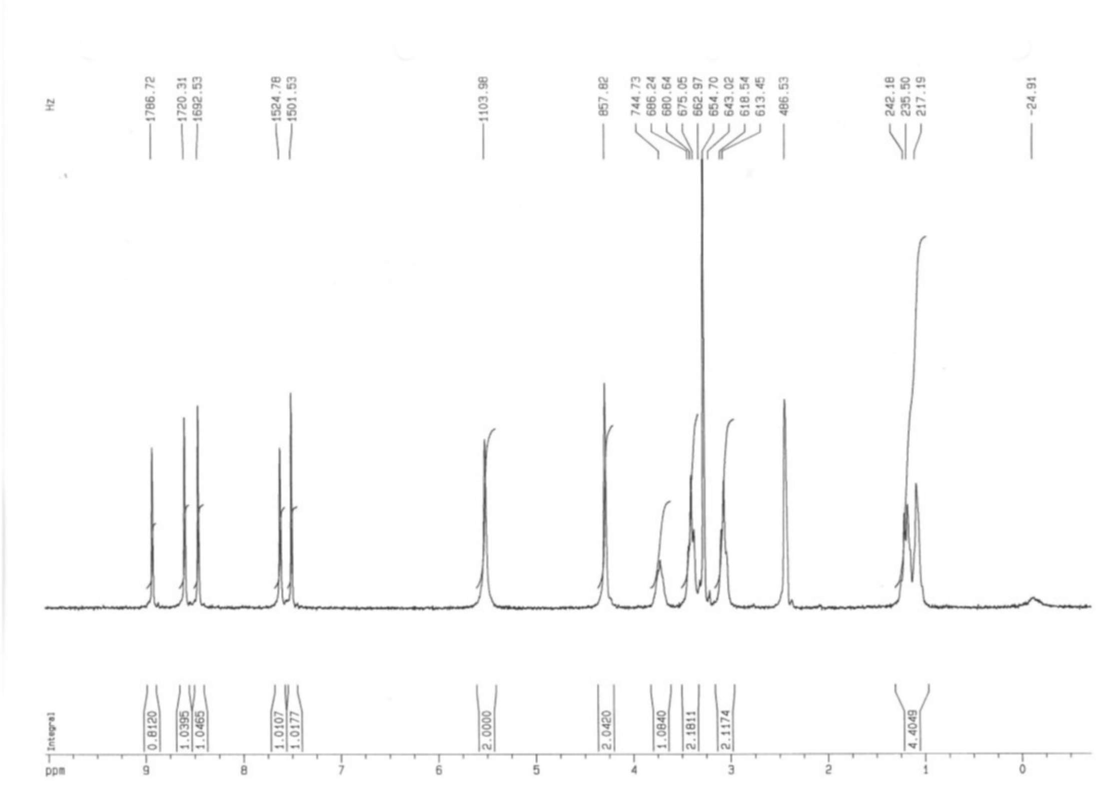

Figure S18. ^{13}C NMR spectrum of compound **18**.

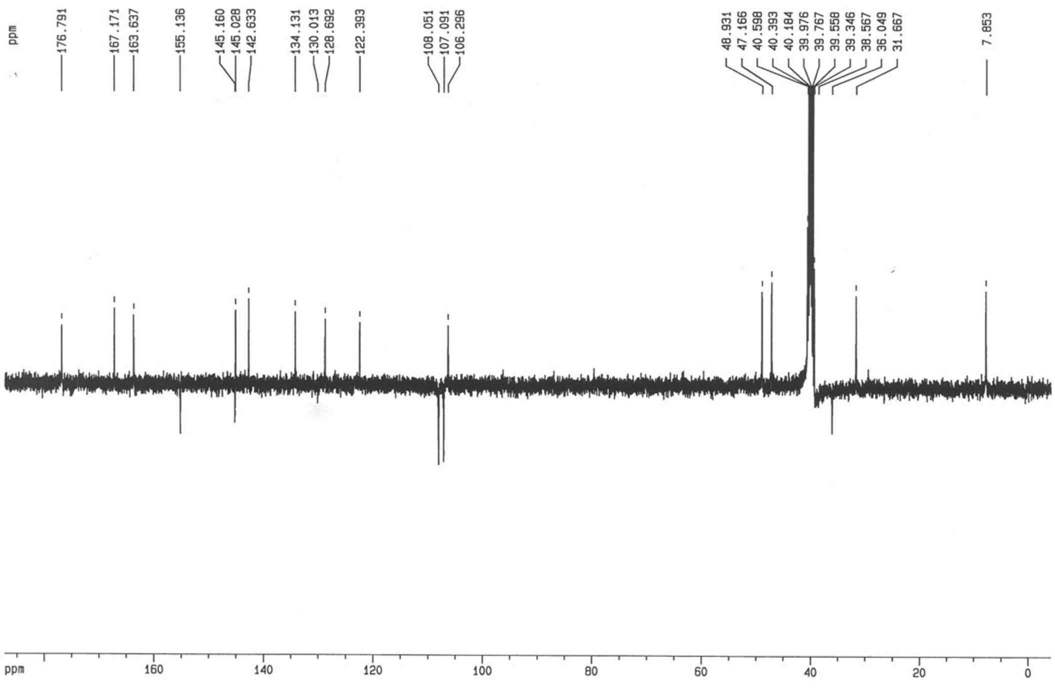
ppm
176.791
167.171
163.637
155.136
145.160
145.028
142.633
134.131
130.013
128.692
122.393
108.051
107.091
106.296
48.931
47.166
40.598
40.393
40.184
39.976
39.767
39.558
39.346
38.567
36.049
31.667
7.853
ppm
160
140
120
100
80
60
40
20
0

Figure S19. ^{1}H NMR spectrum of compound **19**.

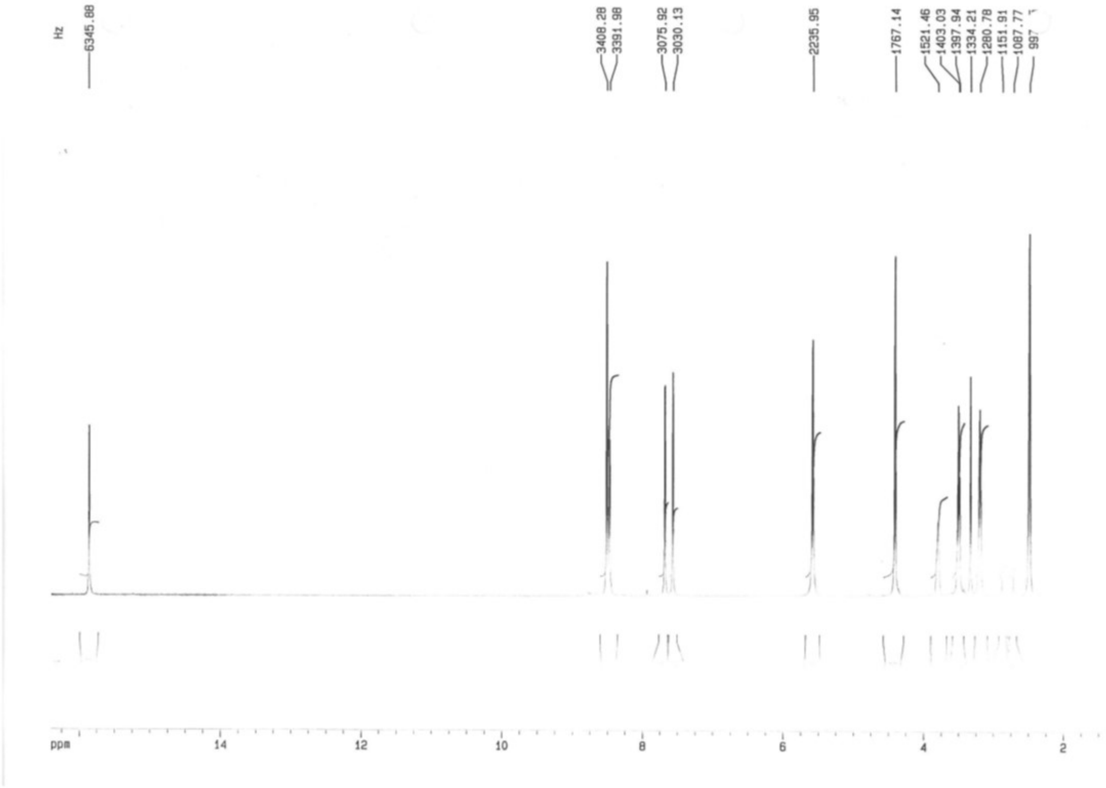

Figure S20. ^{13}C NMR spectrum of compound **19**.

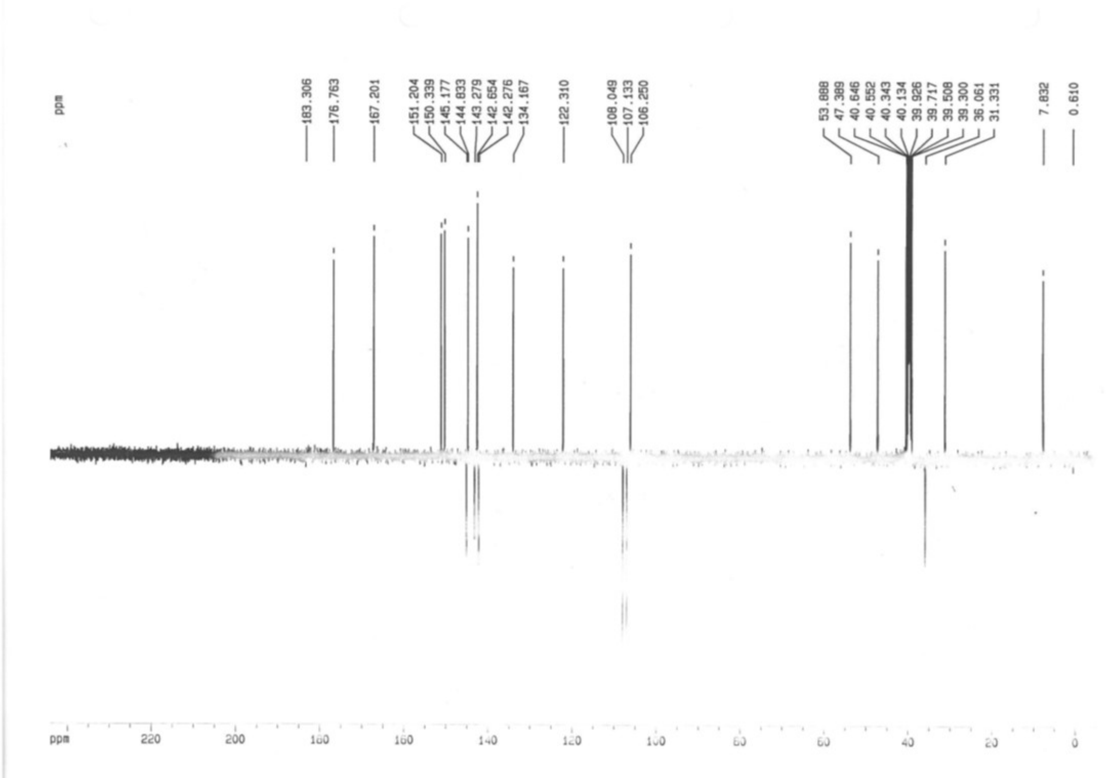

Figure S21. ^{1}H NMR spectrum of compound **20**.

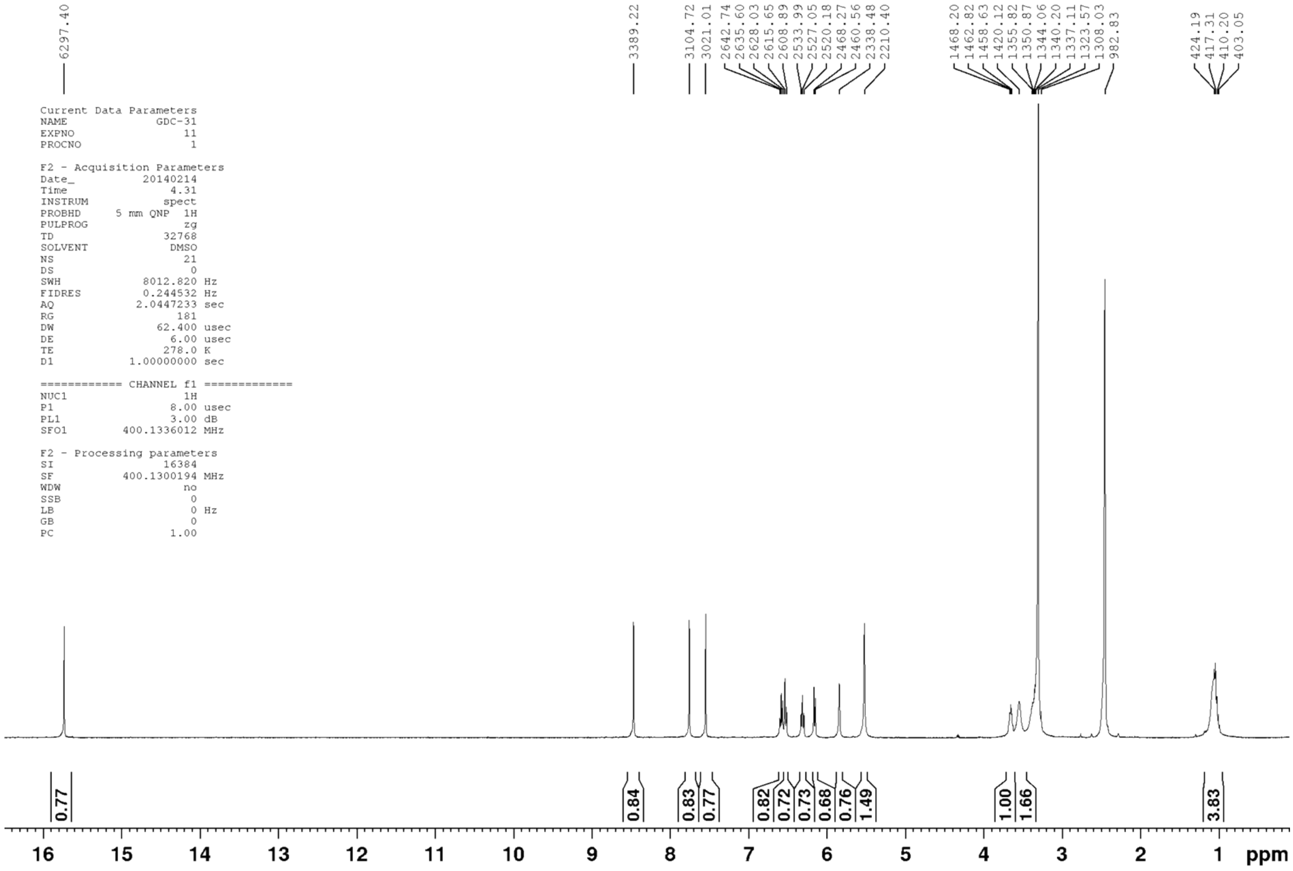

Figure S22. ^{13}C NMR spectrum of compound **20**.

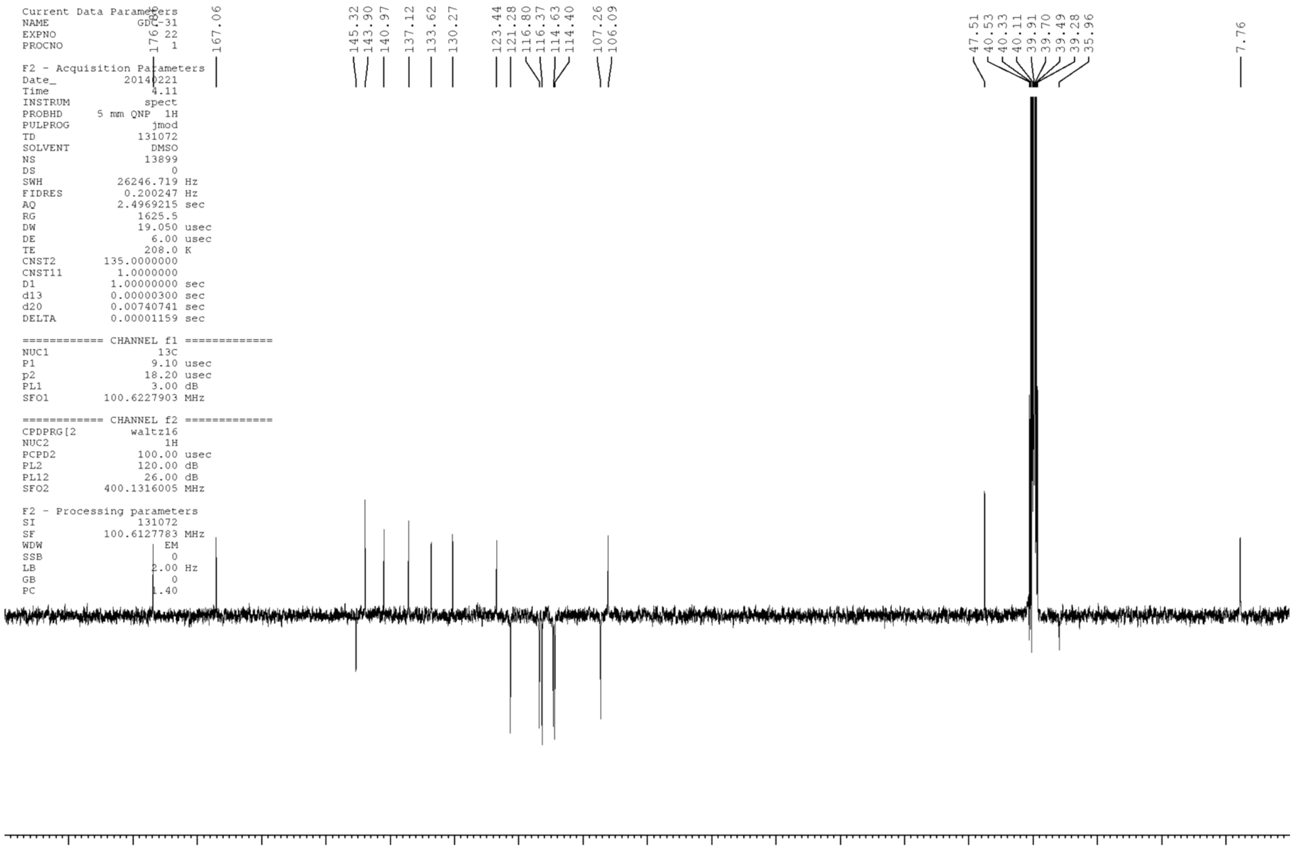

Figure S23. ^{1}H NMR spectrum of compound **21**.

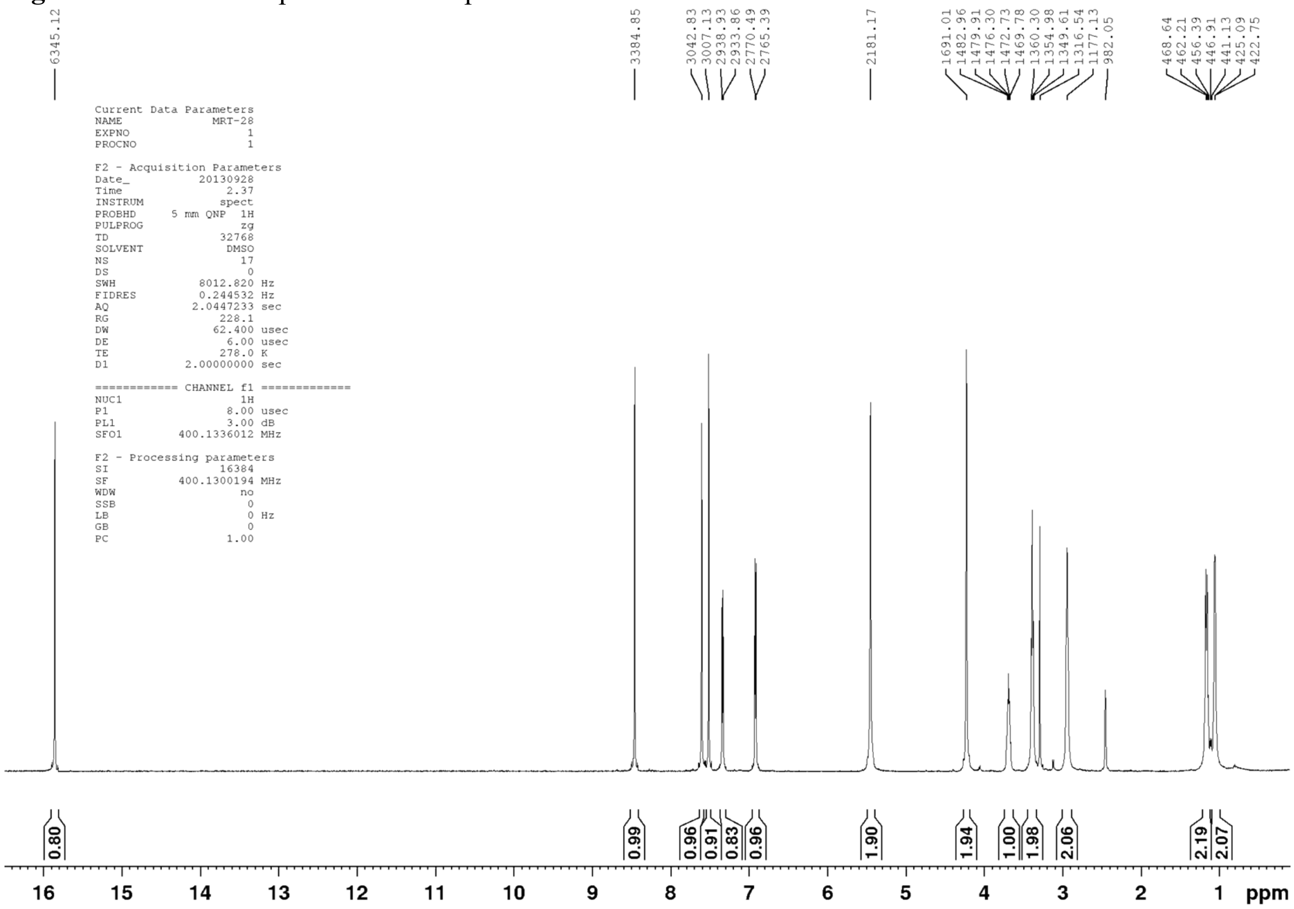

Figure S24. ^{13}C NMR spectrum of compound **21**.

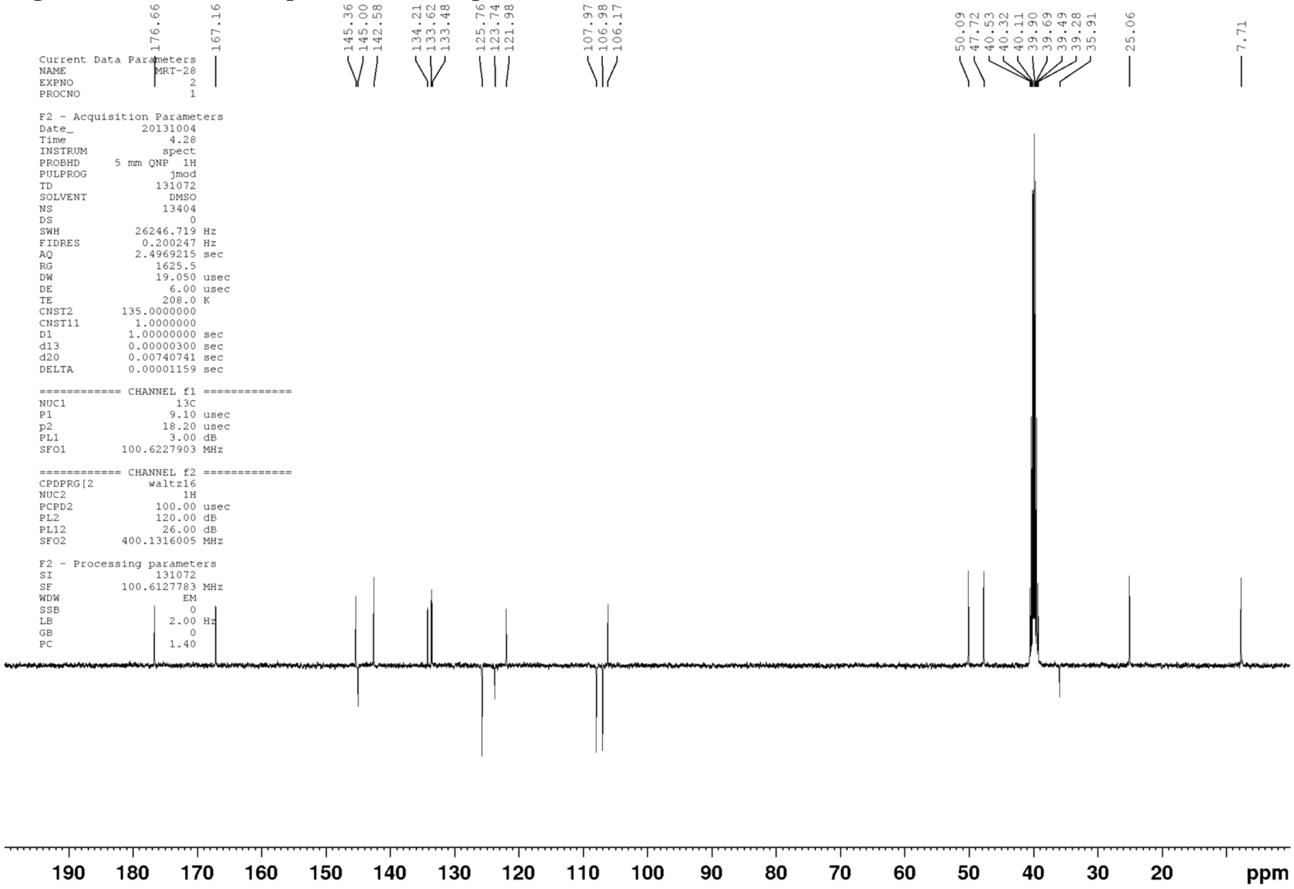

Figure S25. ^{1}H NMR spectrum of compound **22**.

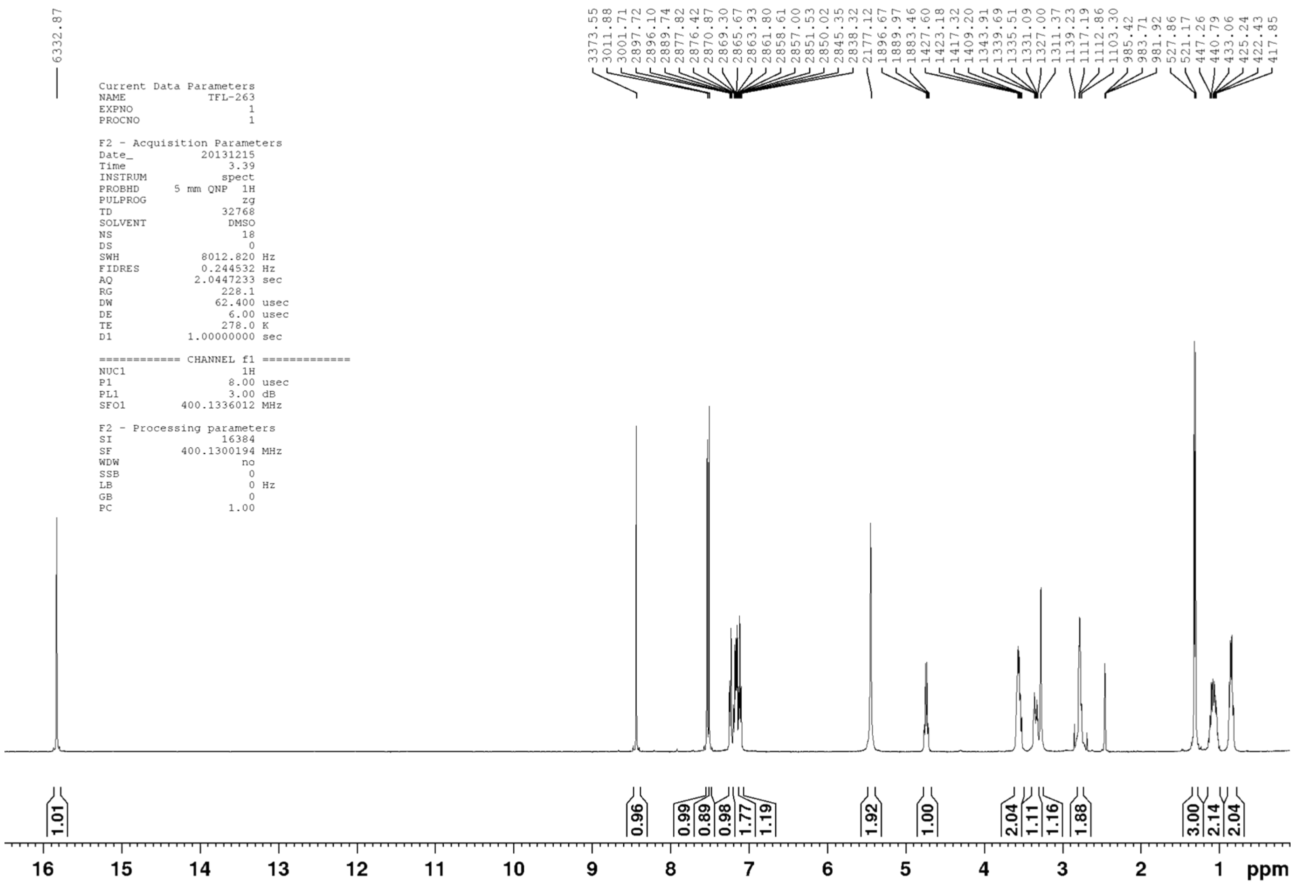

Figure S26. ^{13}C NMR spectrum of compound **22**.

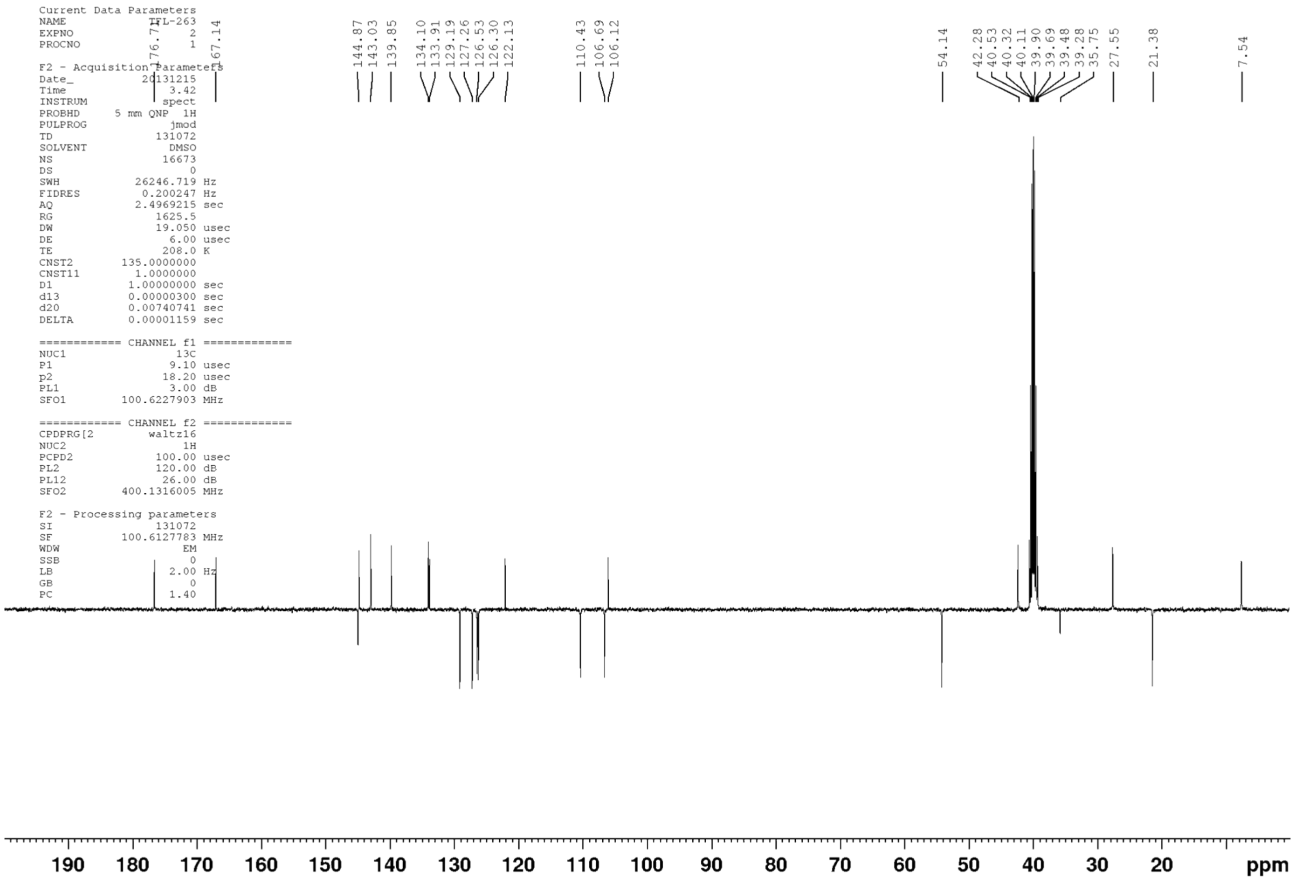

Figure S27. ^{1}H NMR spectrum of compound **23**.

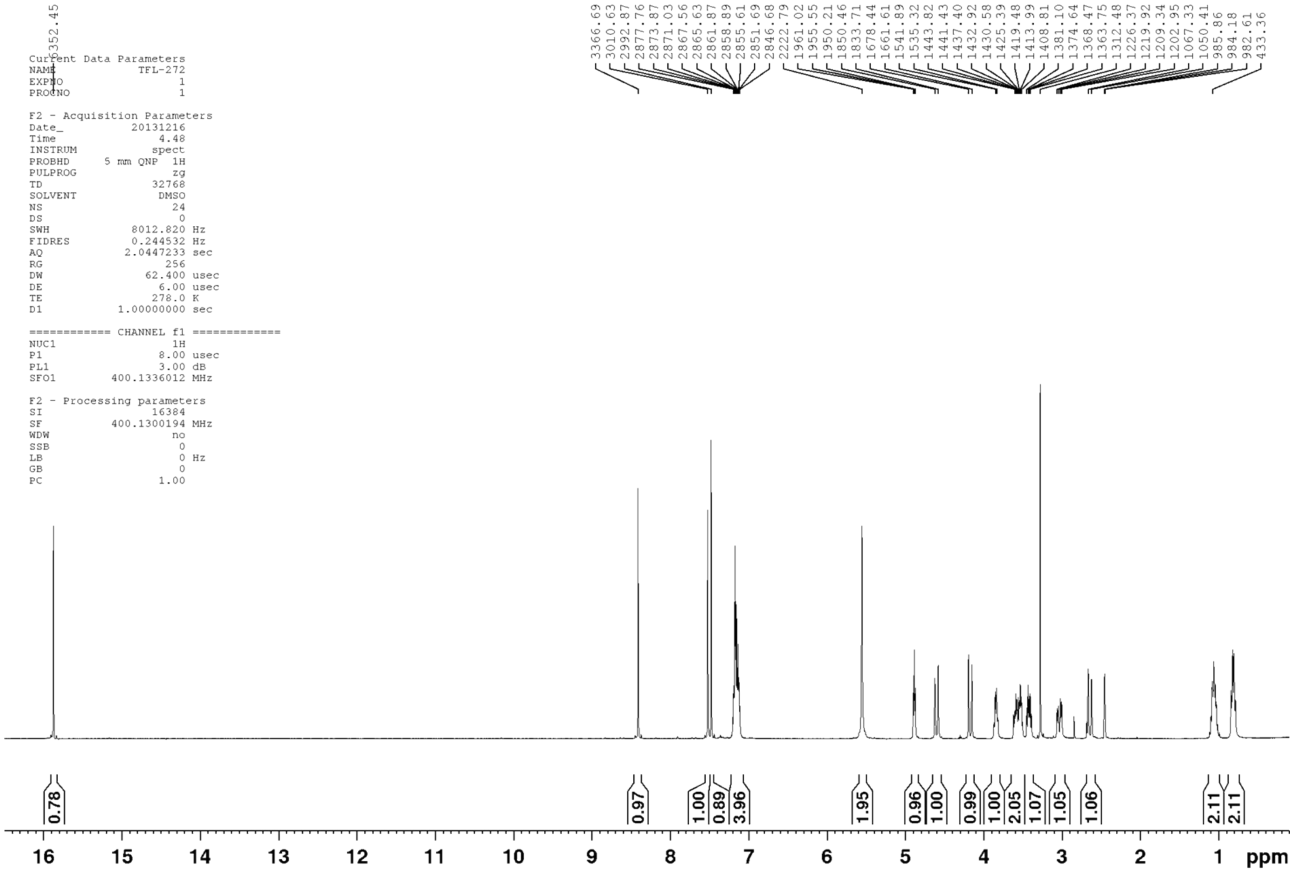

Figure S28. ^{13}C NMR spectrum of compound **23**.

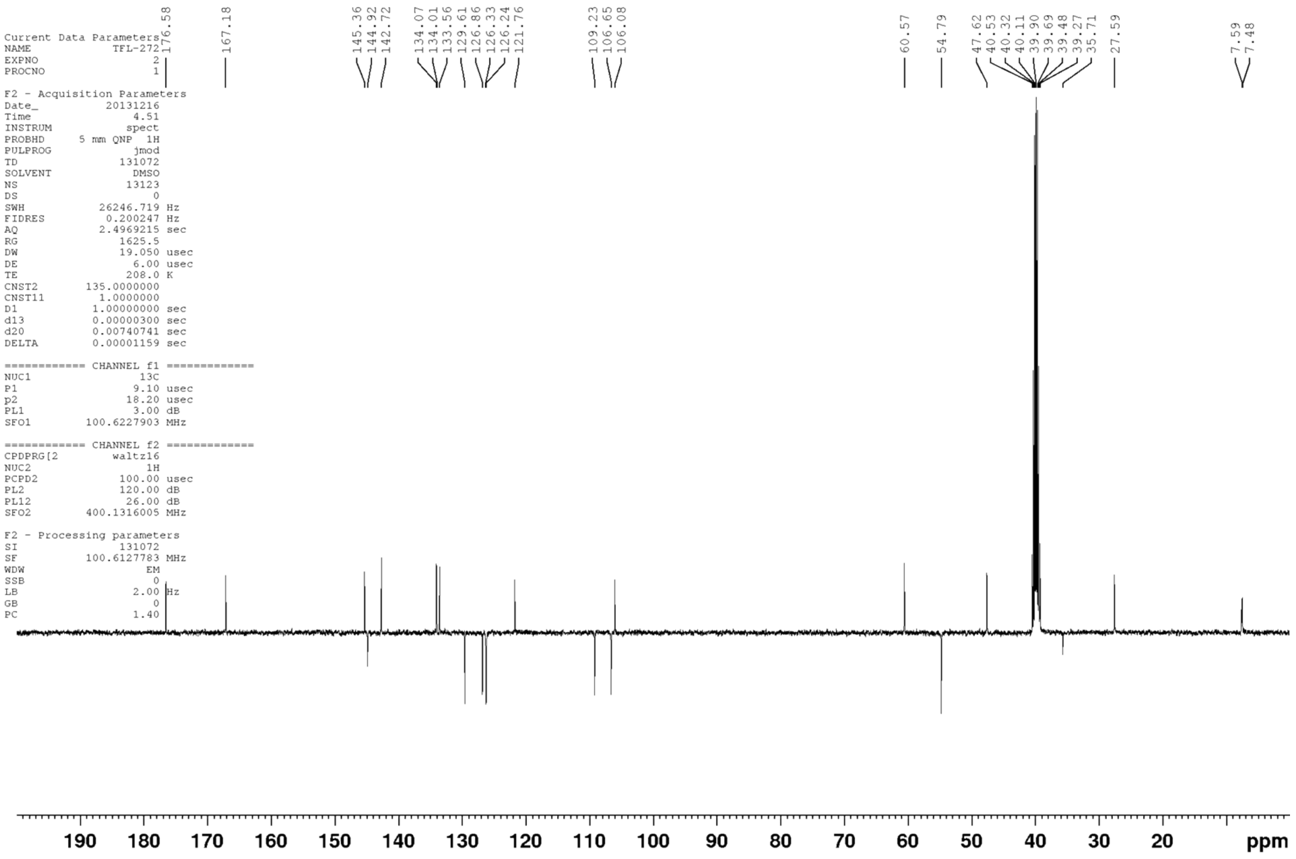

Figure S29. ^{1}H NMR spectrum of compound **24**.

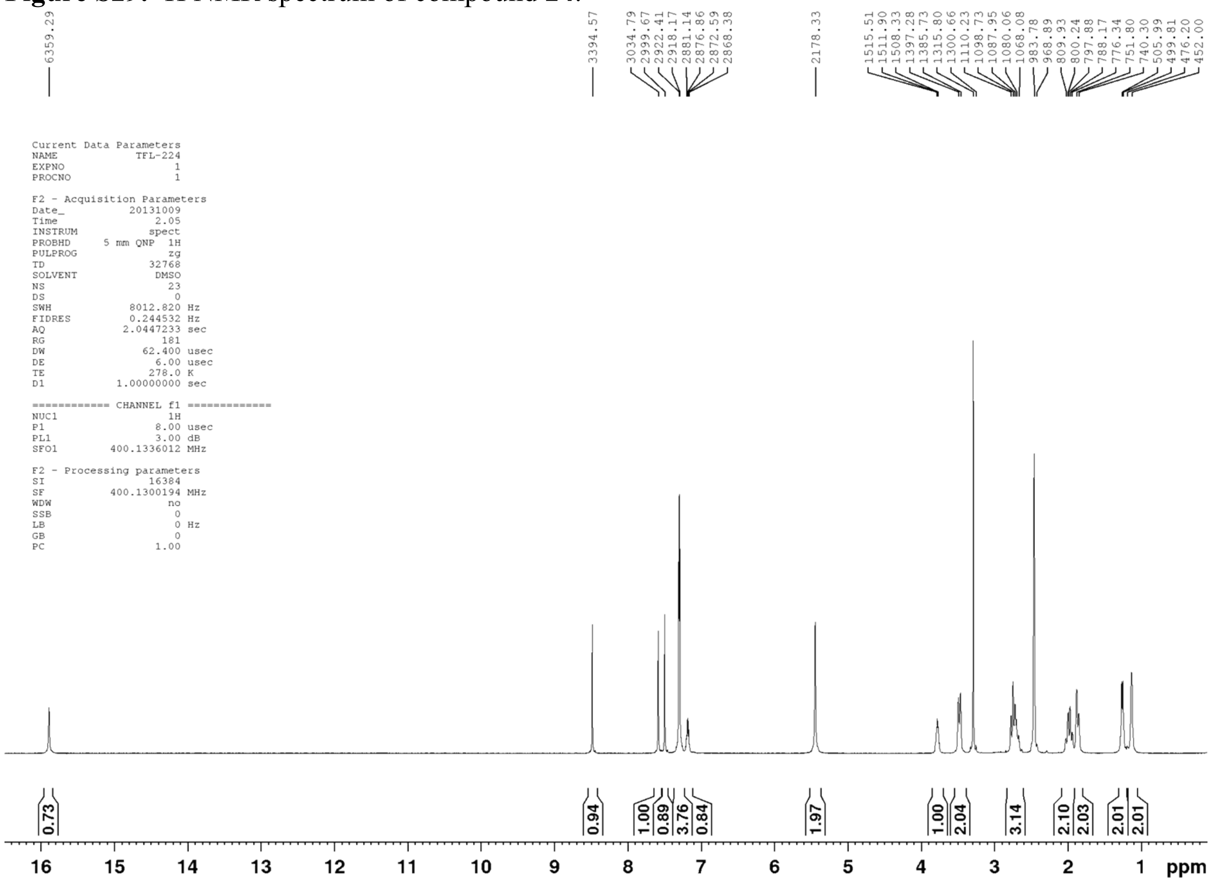

Figure S30. ^{13}C NMR spectrum of compound **24**.

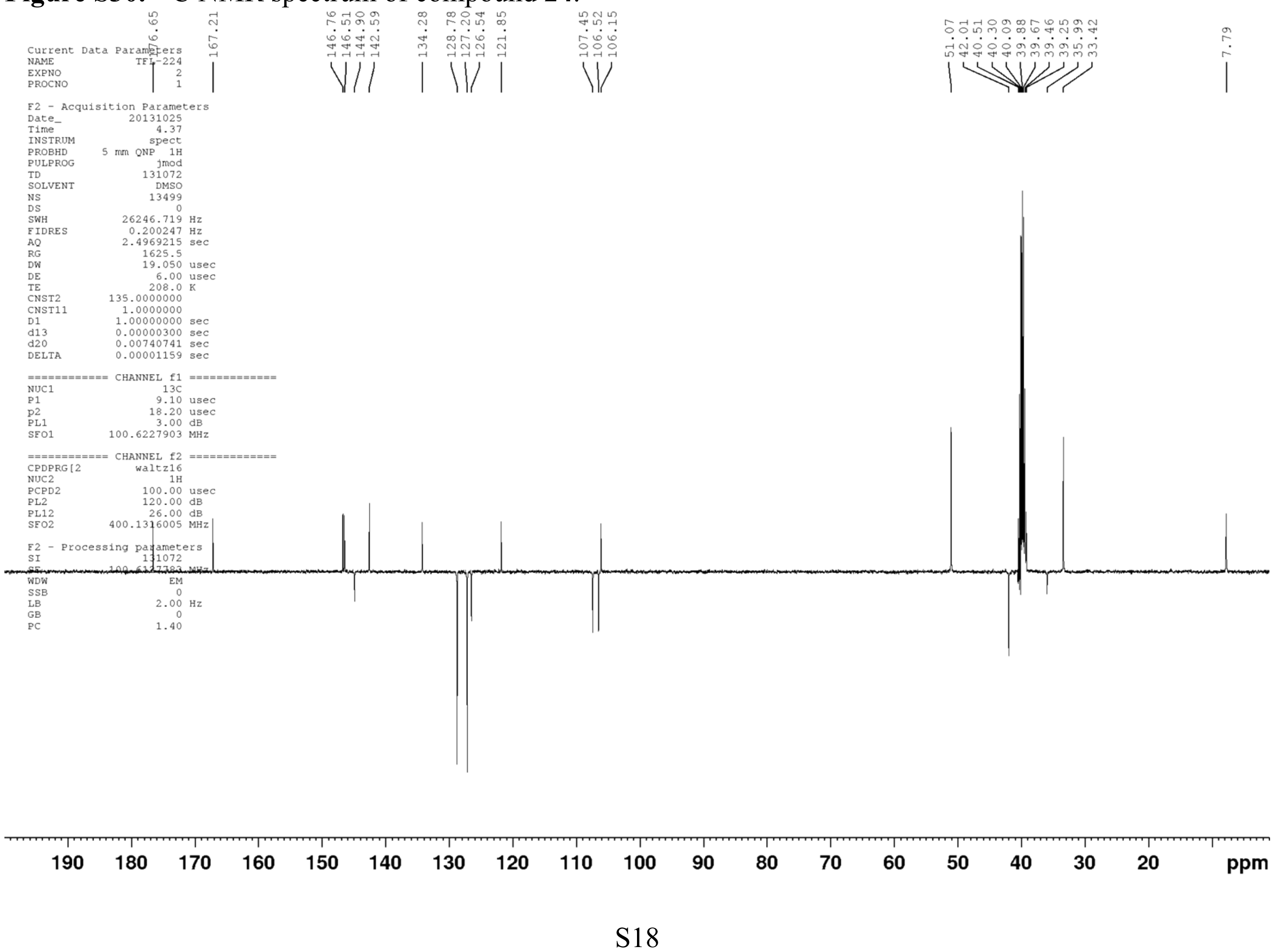

Figure S31. ^{1}H NMR spectrum of compound **25**.

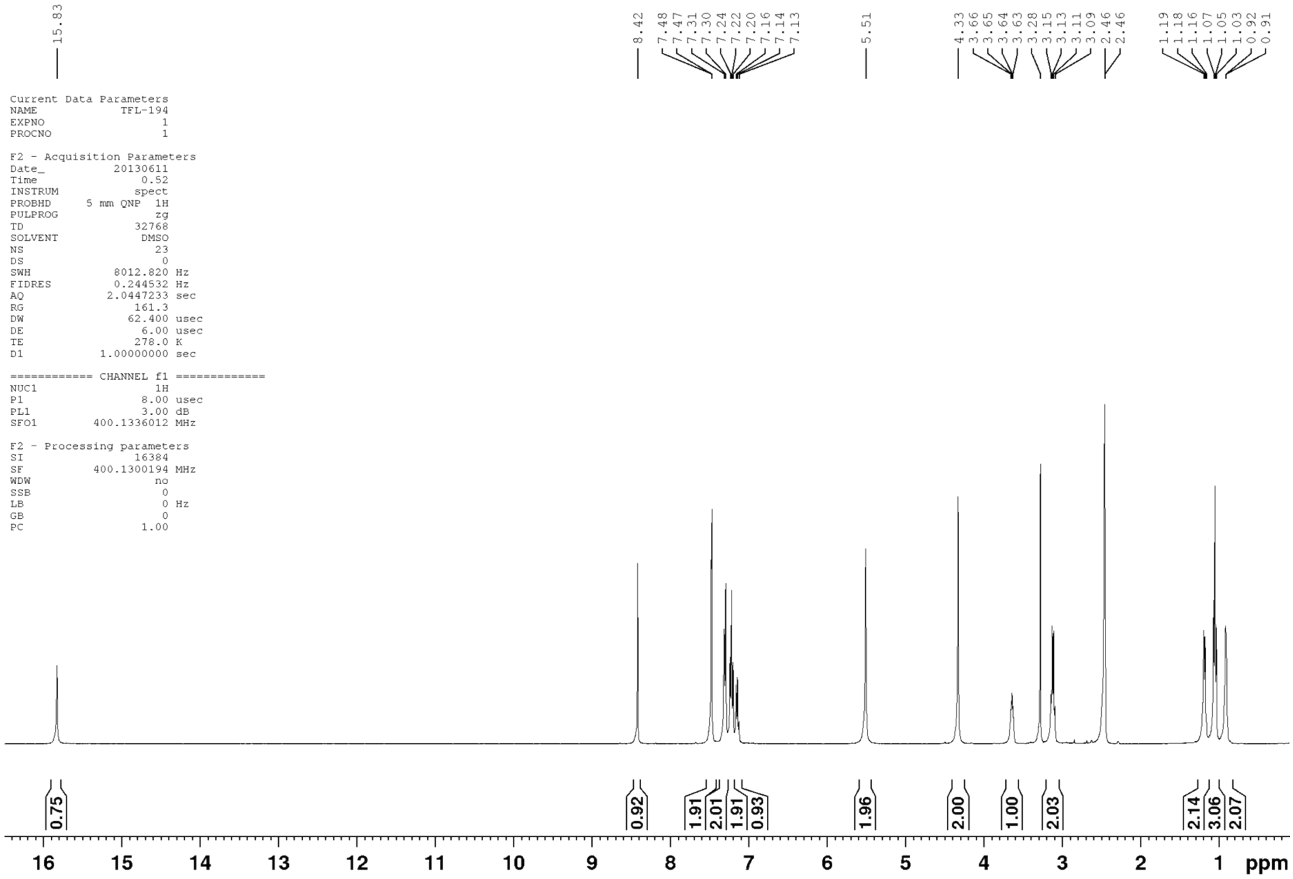

Figure S32. ^{13}C NMR spectrum of compound **25**.

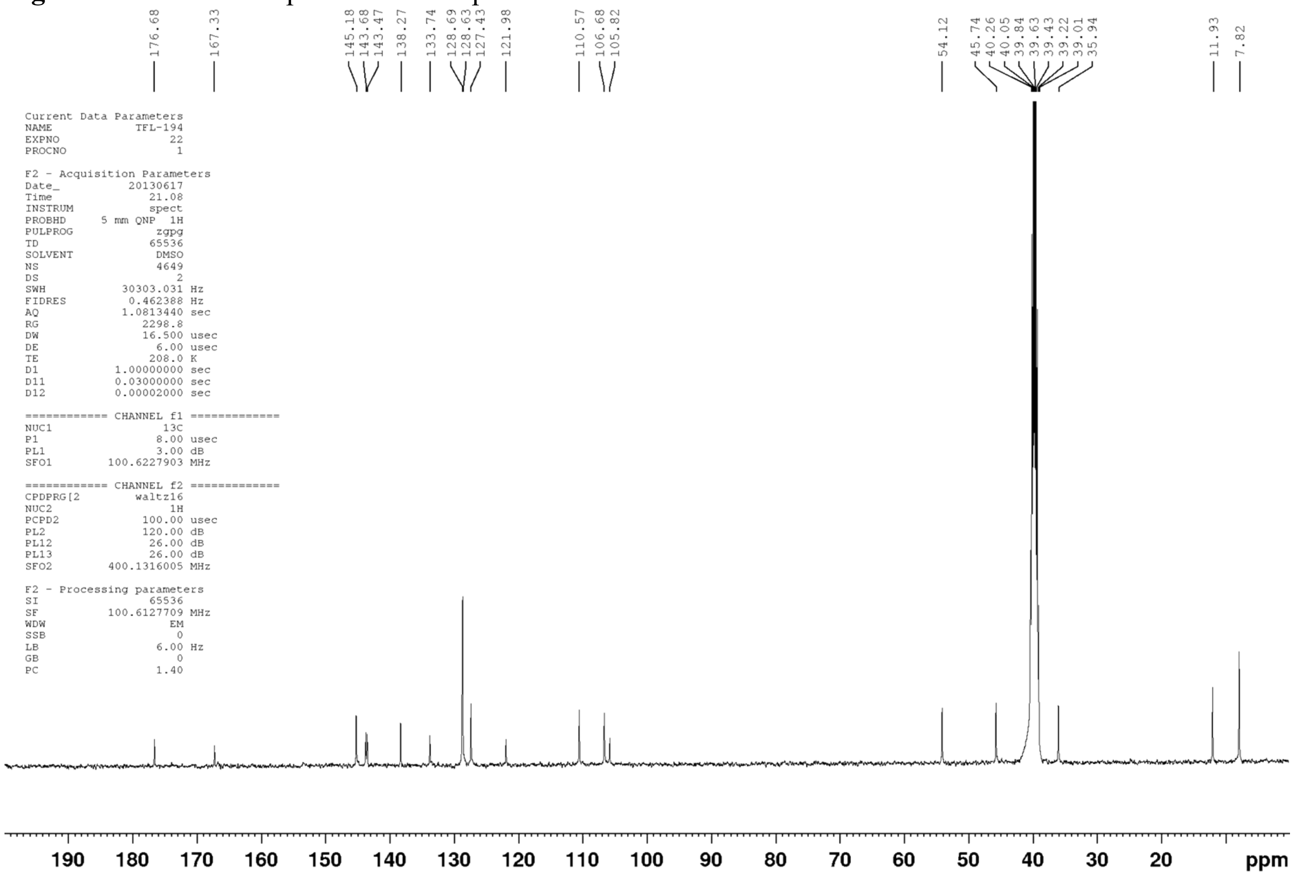

Figure S33. ^{1}H NMR spectrum of compound **26**.

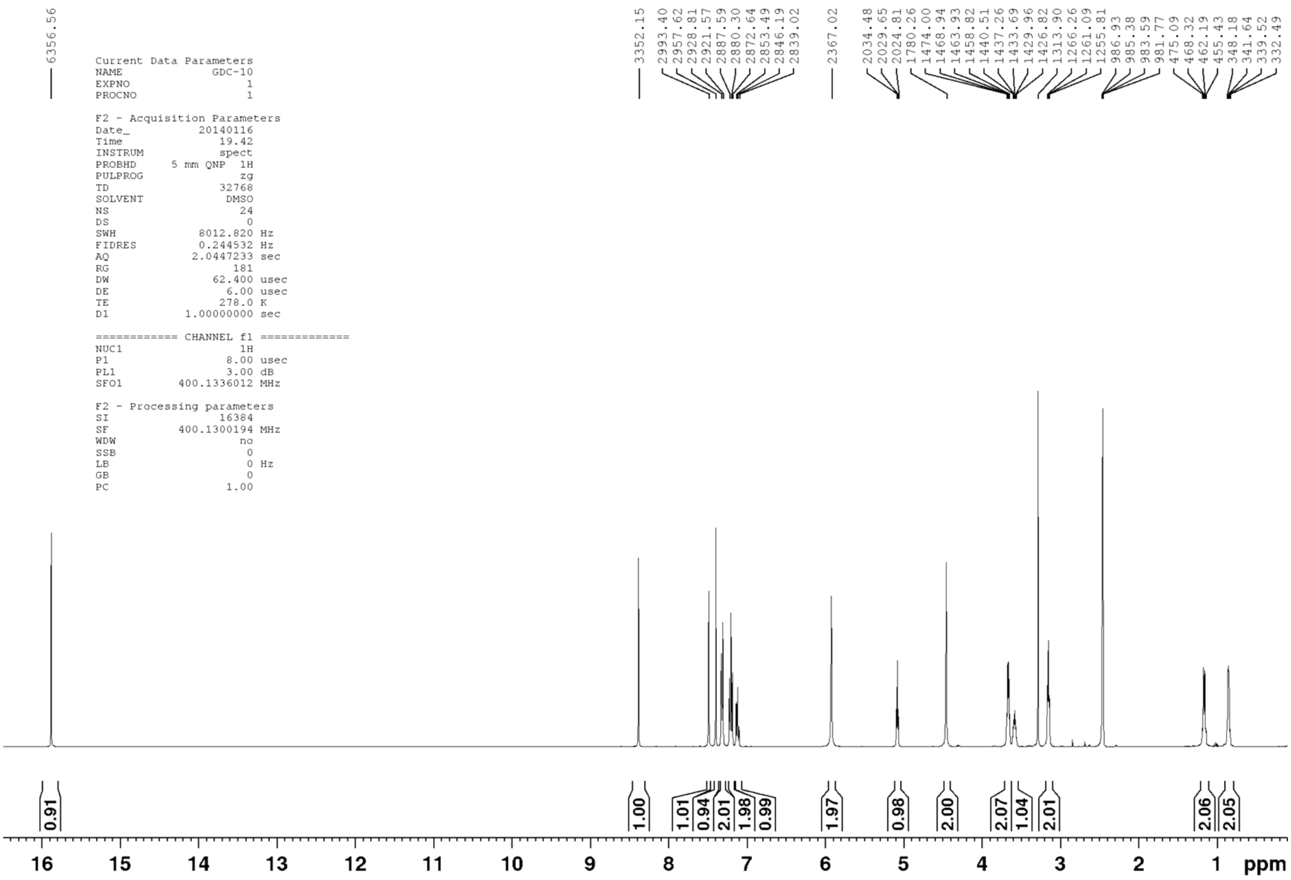

Figure S34. ^{13}C NMR spectrum of compound **26**.

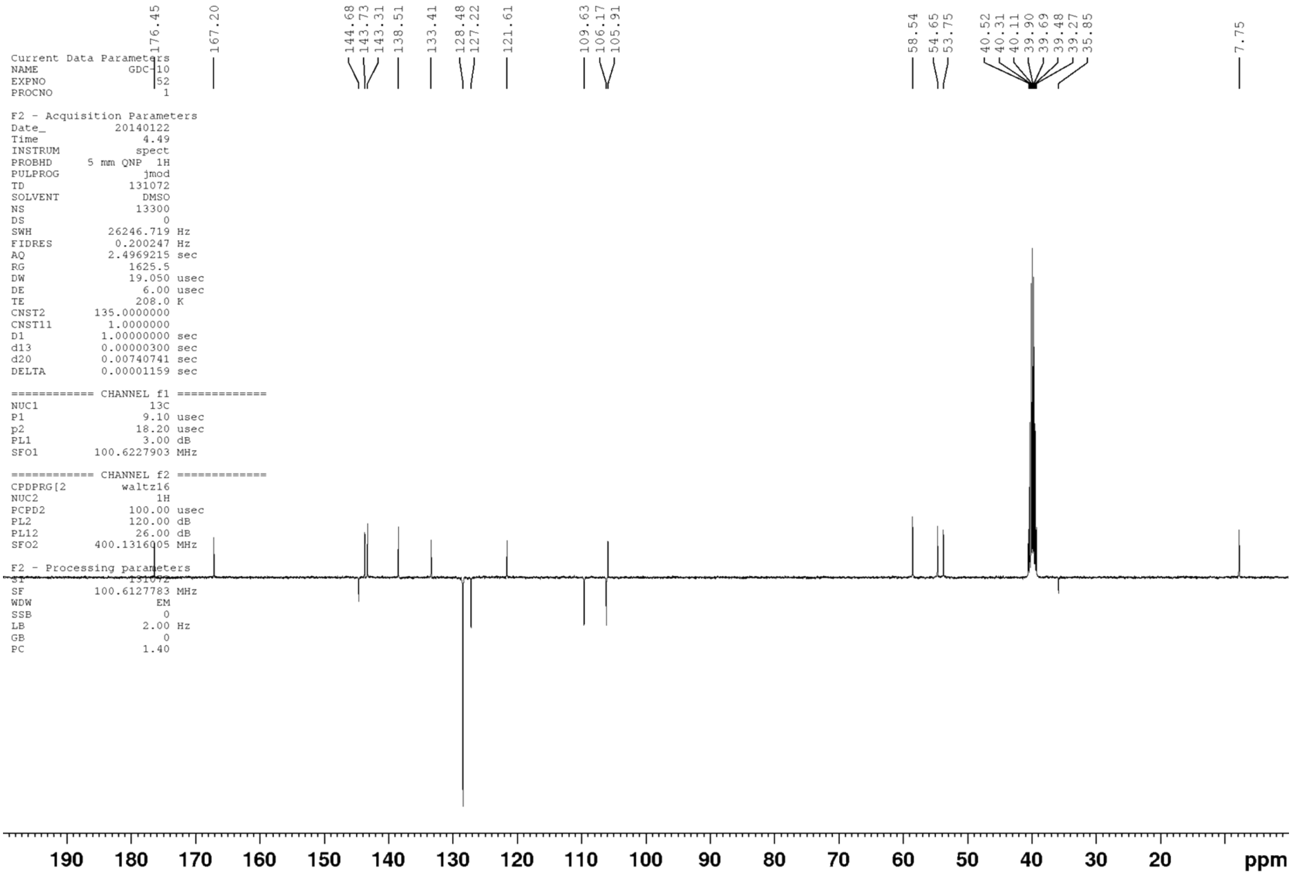

Figure S35. ^{1}H NMR spectrum of compound **27**.

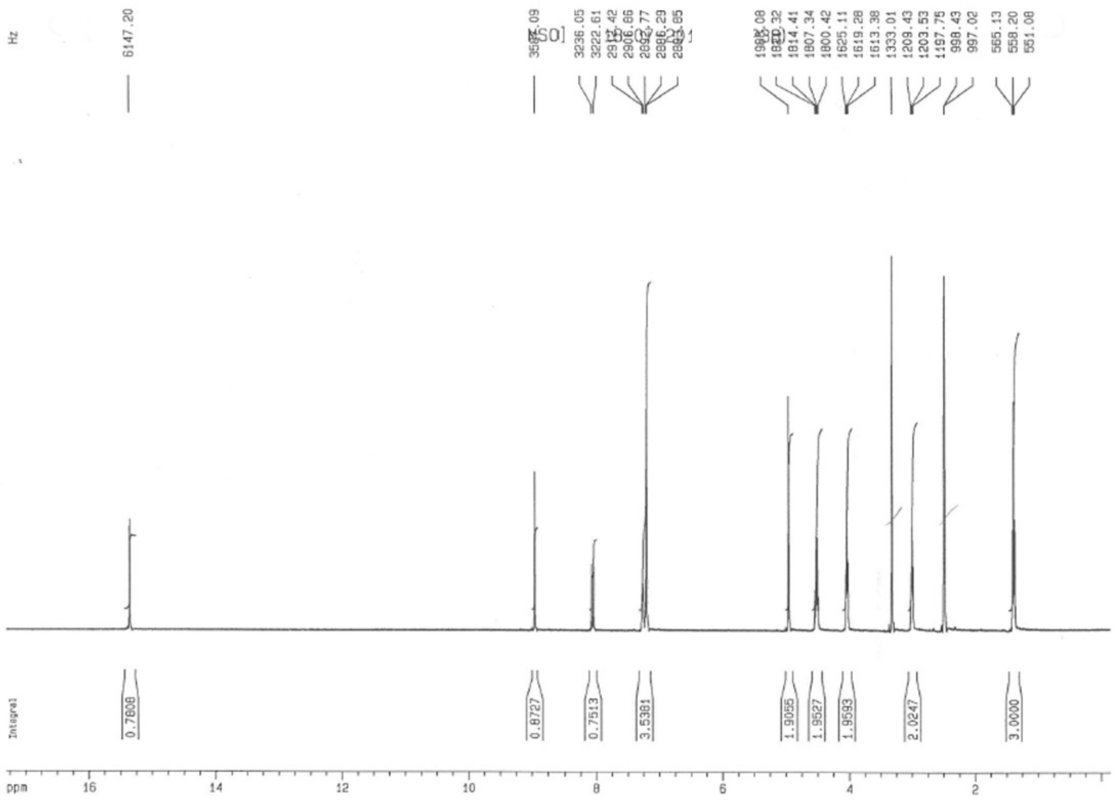

Figure S36. ^{13}C NMR spectrum of compound **27**.

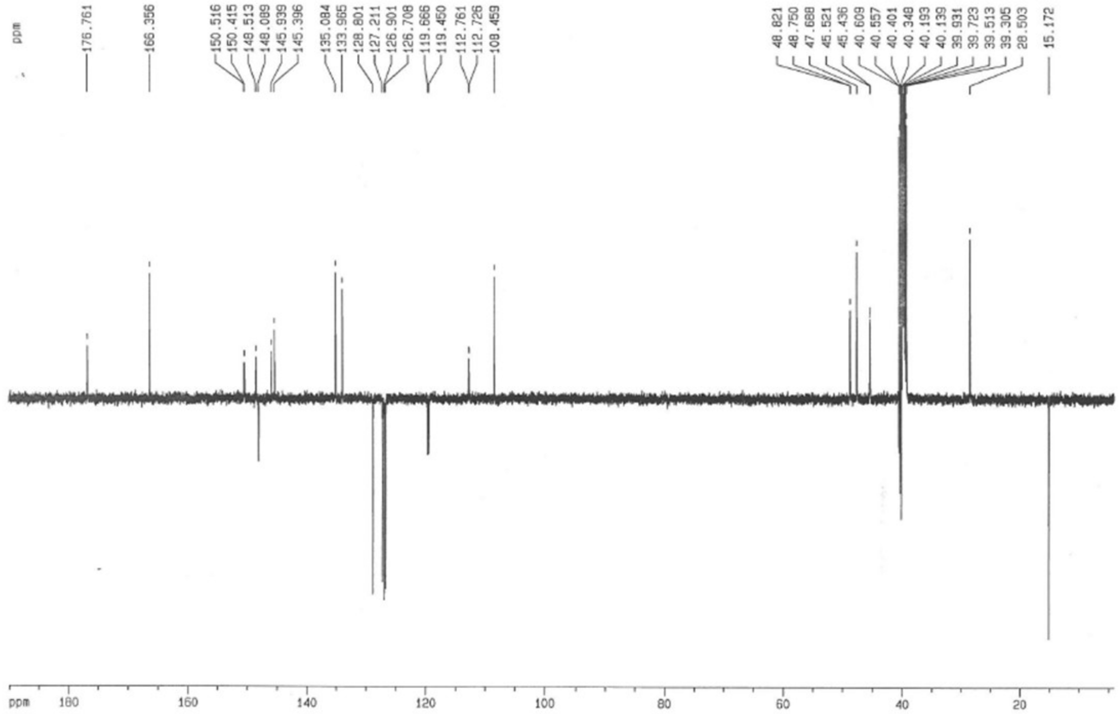

Figure S37. ^{1}H NMR spectrum of compound **28**.

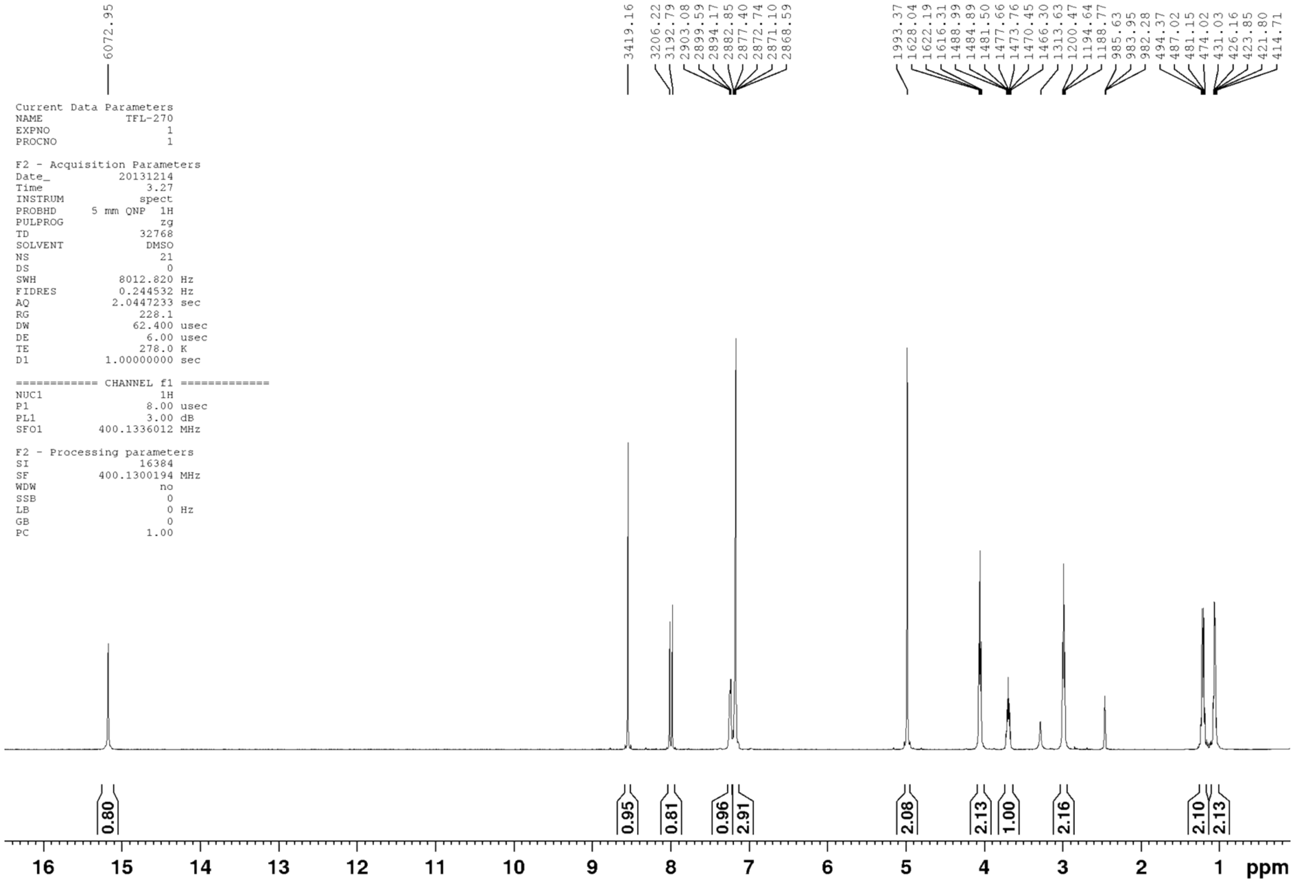

Figure S38. ^{13}C NMR spectrum of compound **28**.

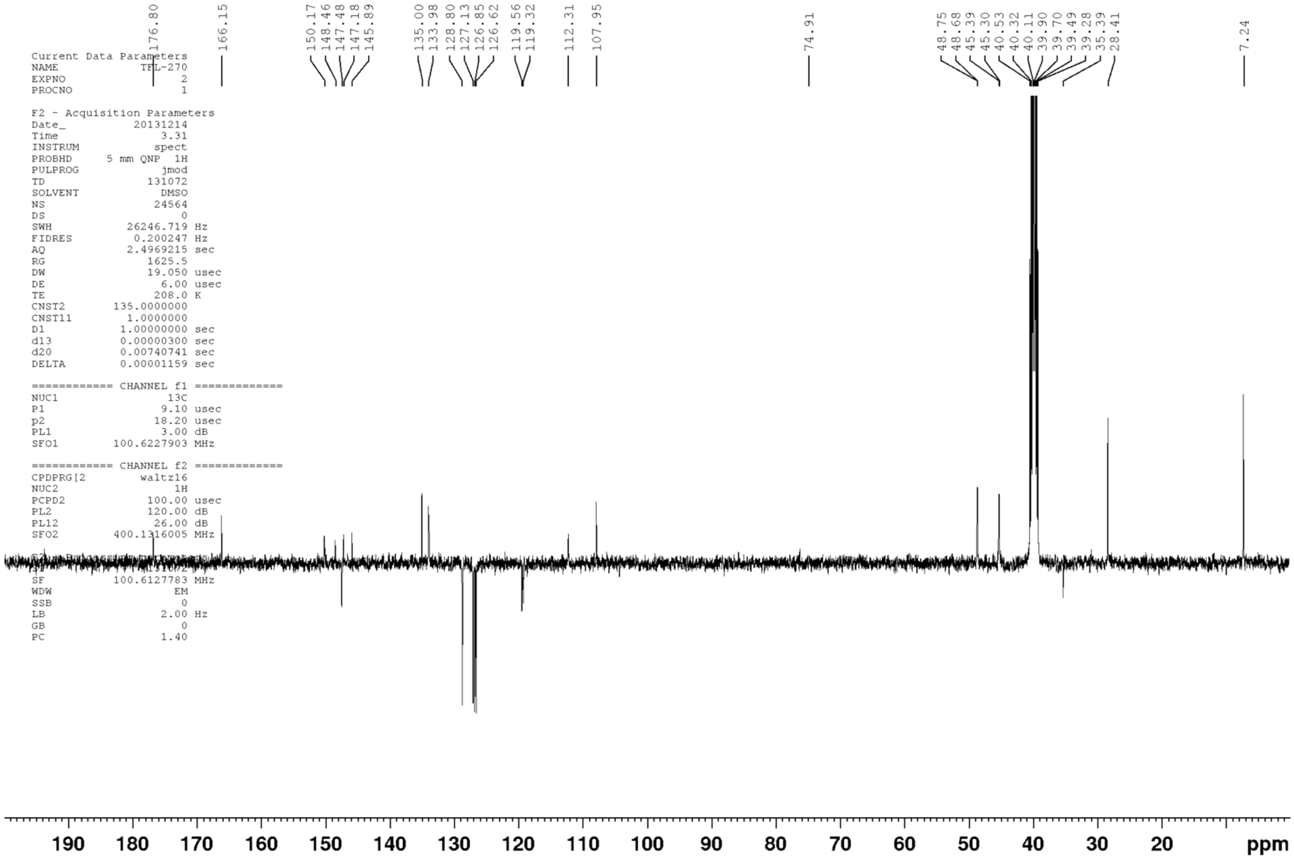

Figure S39. ^{1}H NMR spectrum of compound **29**.

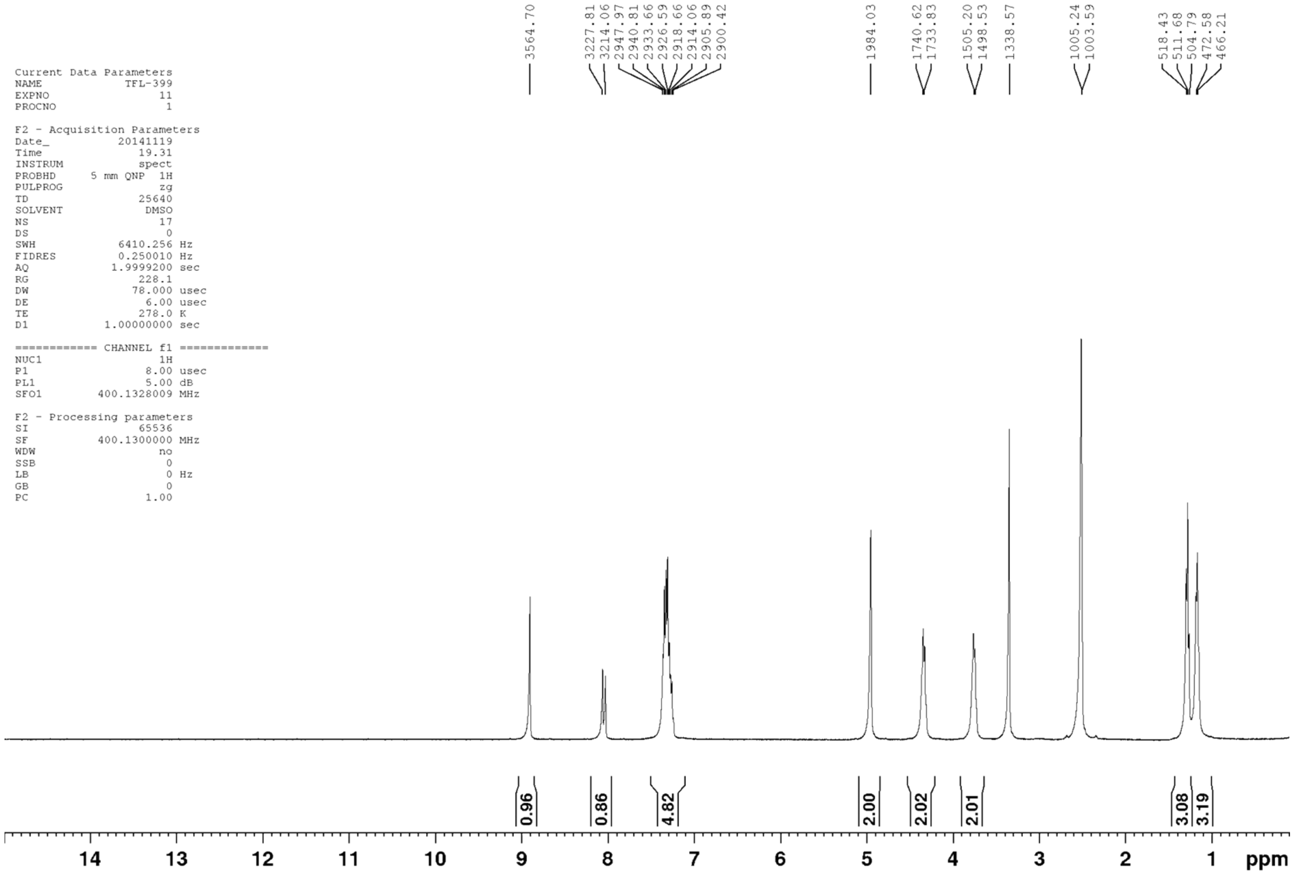

Figure S40. ^{13}C NMR spectrum of compound **29**.

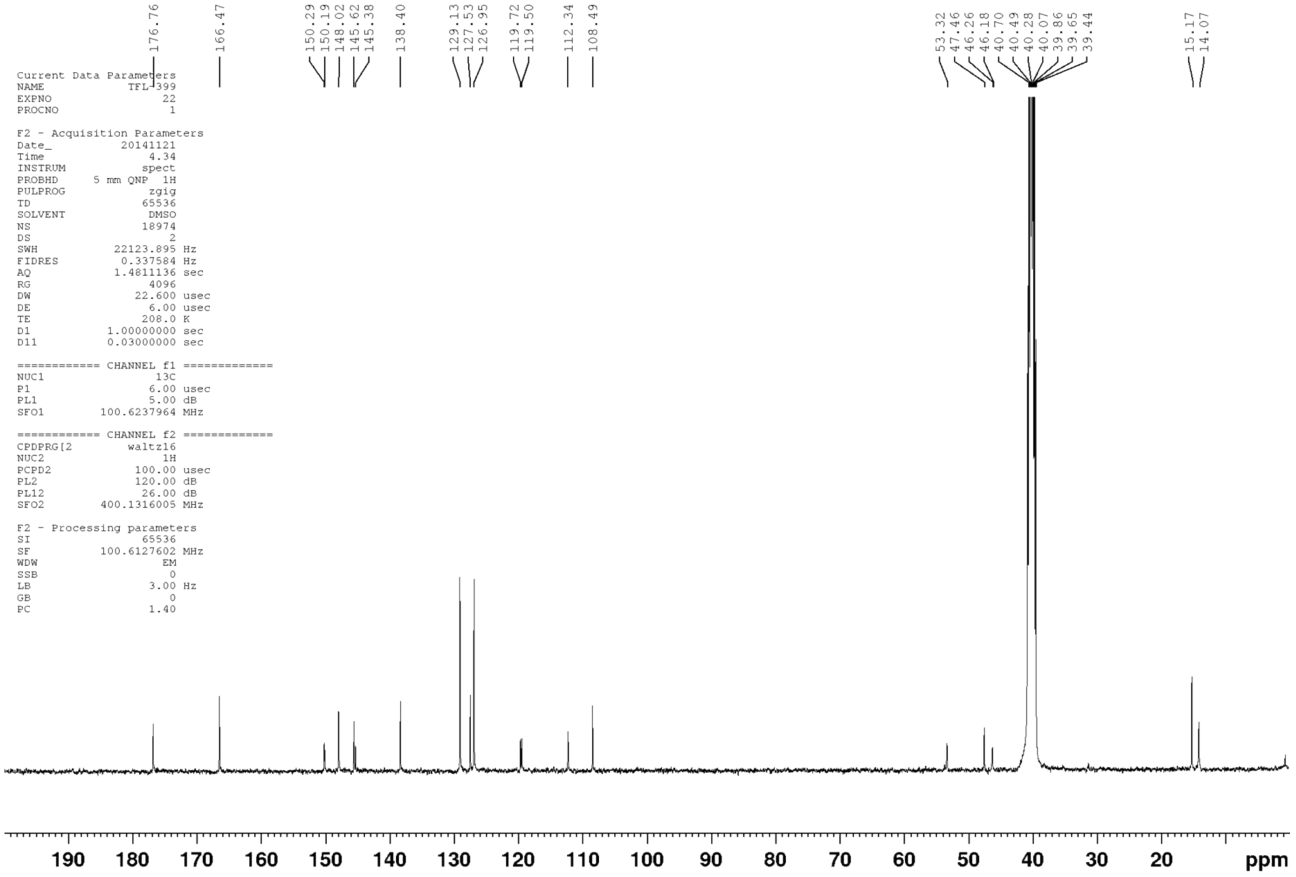

Figure S41. ^{1}H NMR spectrum of compound **30**.

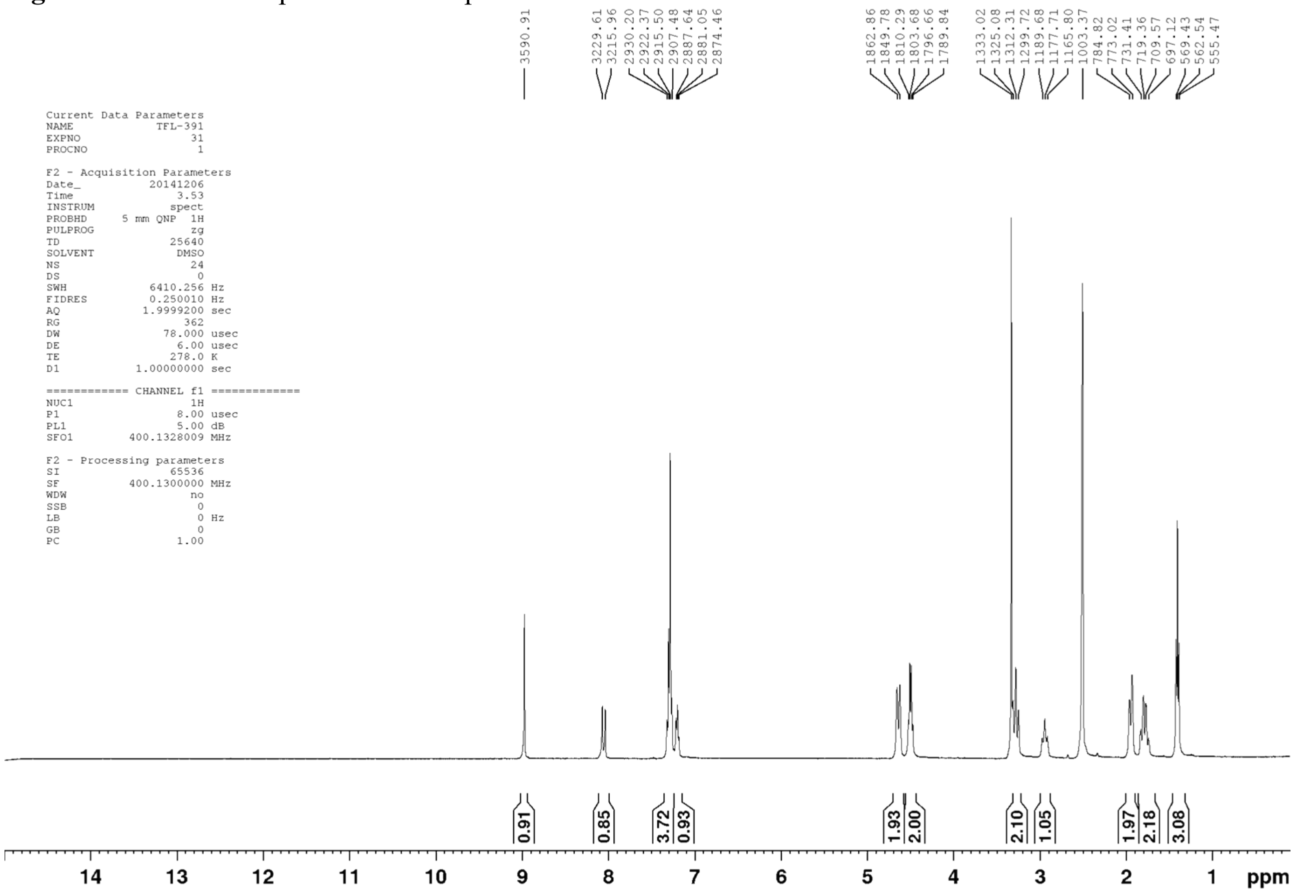

Figure S42. ^{13}C NMR spectrum of compound **30**.

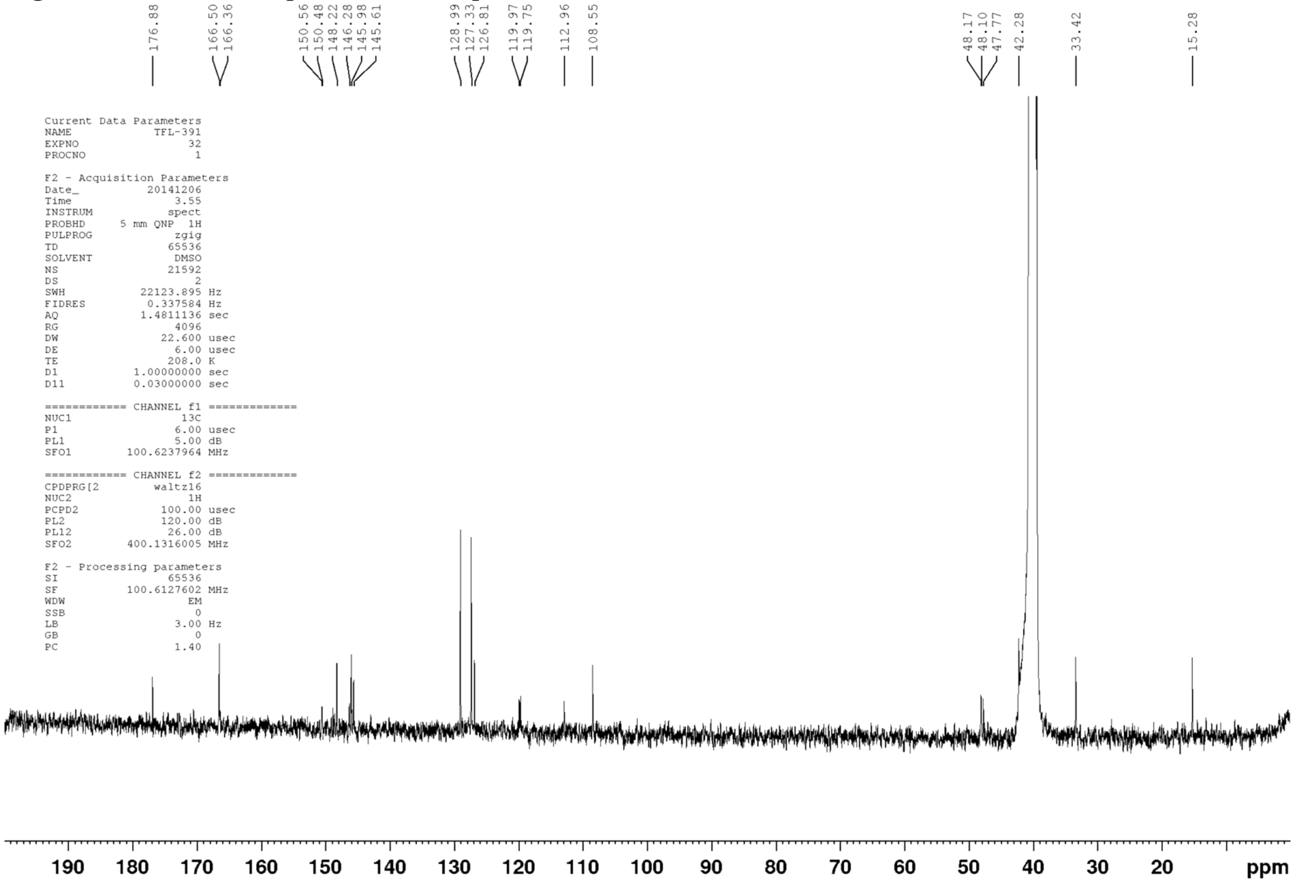

Figure S43. ^{1}H NMR spectrum of compound **31**.

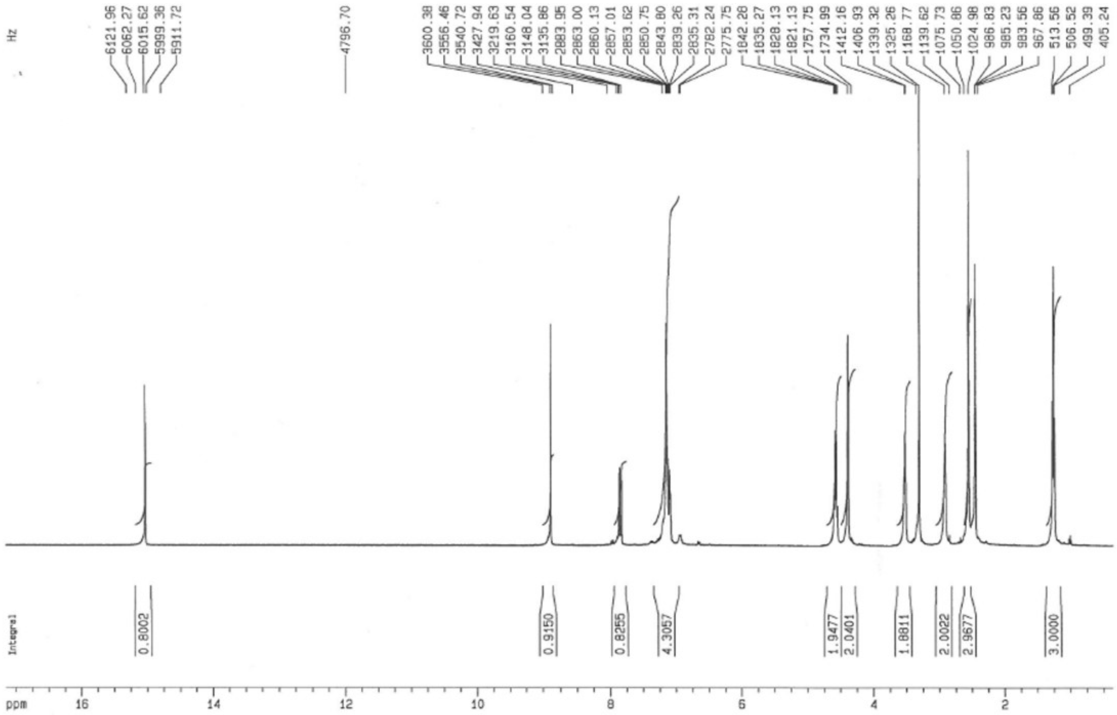

Figure S44. ^{13}C NMR spectrum of compound **31**.

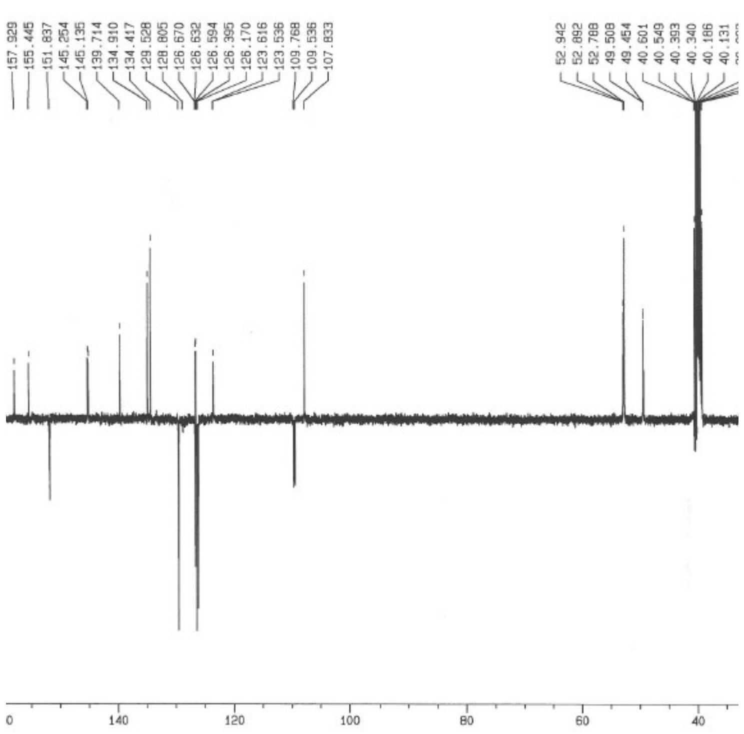
157.929
155.445
151.837
145.254
145.135
139.714
134.910
134.417
129.528
128.805
126.670
126.632
126.594
126.395
126.170
123.616
123.536
109.768
109.536
107.833
52.942
52.892
52.788
49.508
49.454
40.601
40.549
40.393
40.340
40.186
40.131
140
120
100
80
60
40

Figure S45. ^{1}H NMR spectrum of compound **32**.

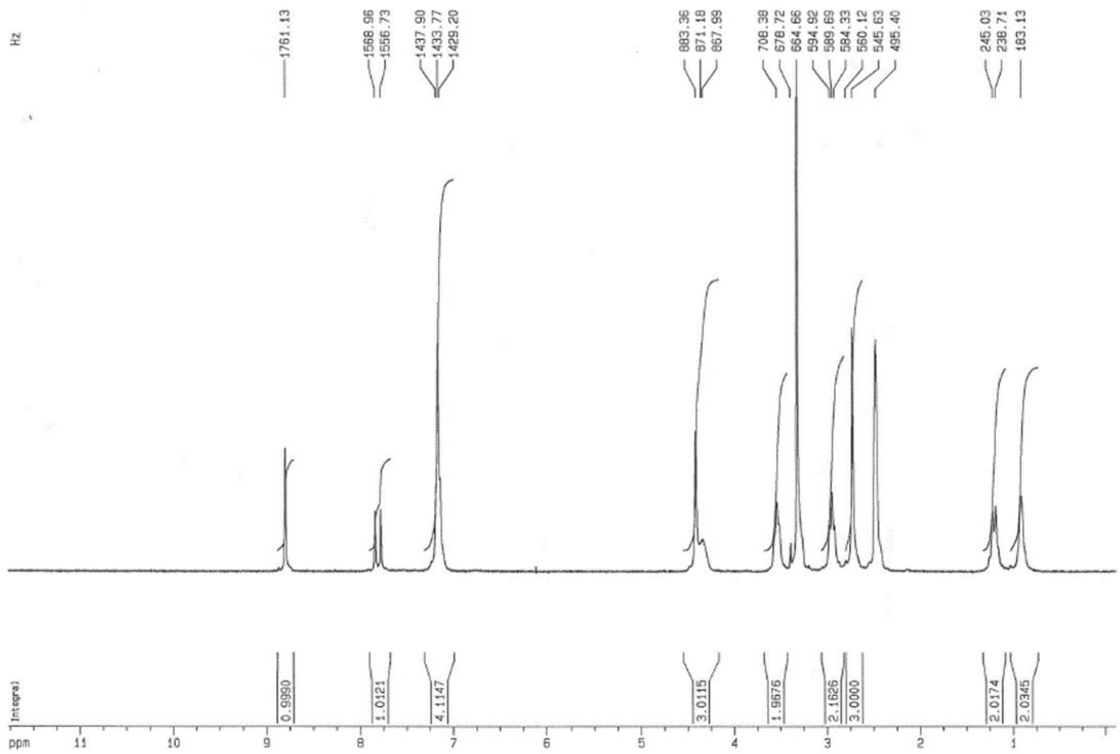

Figure S46. ^{13}C NMR spectrum of compound **32**.

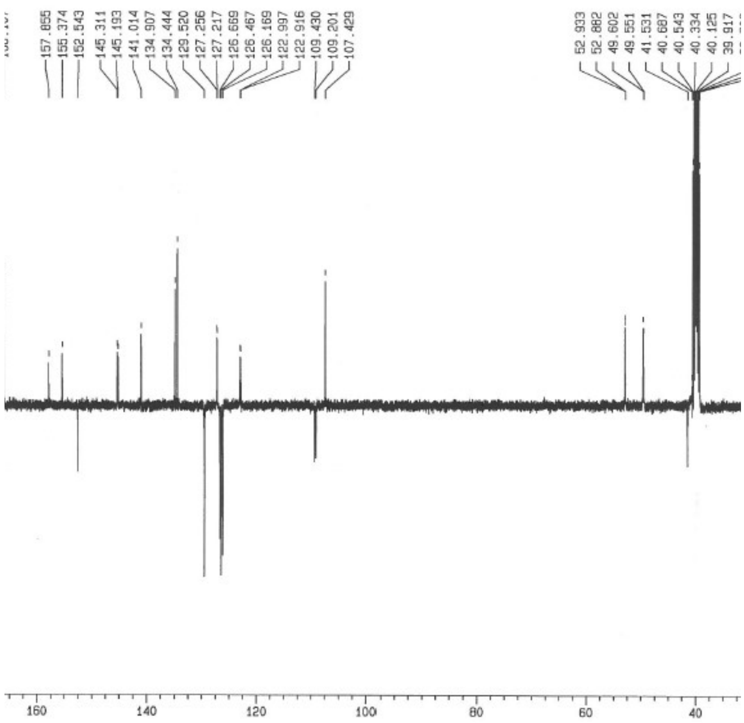
157.855
155.374
152.543
145.311
145.193
141.014
134.907
134.444
129.520
127.256
127.217
126.669
126.467
126.169
122.997
122.916
109.430
109.201
107.429
52.933
52.882
49.602
49.551
41.531
40.687
40.543
40.334
40.125
39.917
160
140
120
100
80
60
40

Figure S47. ^{1}H NMR spectrum of compound **33**.

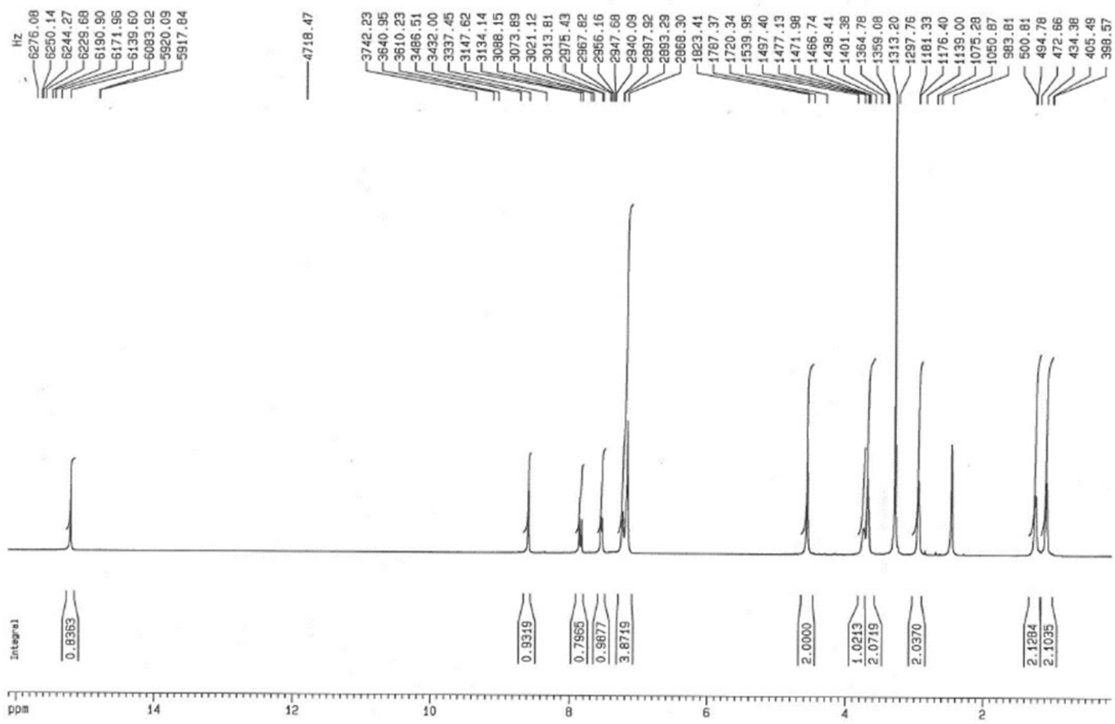

Figure S48. ^{13}C NMR spectrum of compound **33**.

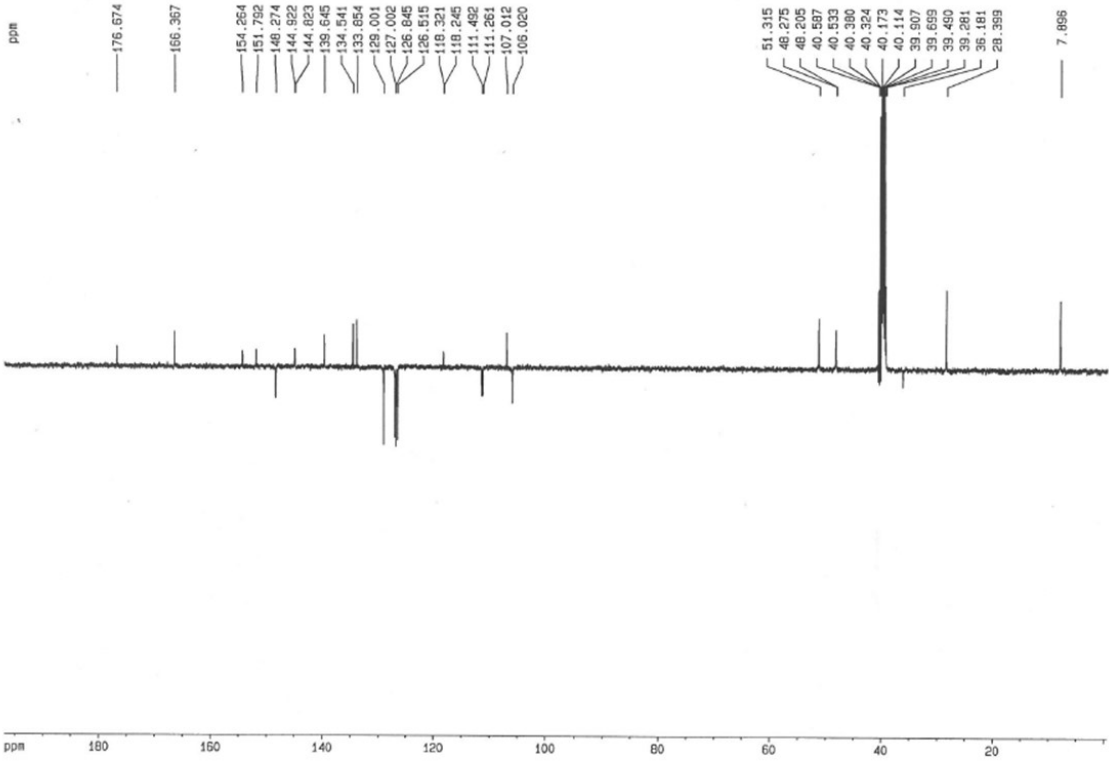

Supplement: MD-016-D4MD00649F-s001 [file MD-016-D4MD00649F-s001.pdf]
